# Supplementary material for: A phase 1/2 clinical trial of invariant natural killer T cell therapy in moderate-severe acute respiratory distress syndrome
Source: Nat Commun. 2024 Feb 6;15:974. doi: 10.1038/s41467-024-44905-z (PMC10847411; doi:10.1038/s41467-024-44905-z)
Supplement: Supplementary file 1 — Supplementary Information [file 41467_2024_44905_MOESM1_ESM.pdf]

# SUPPLEMENTARY INFORMATION

## A phase 1/2 clinical trial of invariant natural killer T cell therapy in moderate-severe acute respiratory distress syndrome

Terese C. Hammond[1,2ᵀ], Marco A. Purbhoo[3ᵀ], Sapana Kadel[3], Jerome Ritz[4], Sarah Nikiforow[4], Heather Daley[4], Kit Shaw[4], Koen Van Besien[5], Alexandra Gomez-Arteaga[6], Don Stevens[7], Waldo Ortuzar[8], Xavier Michelet[3], Rachel Smith[3], Darrian Moskowitz[3], Reed Masakayan[3], Burcu Yigit[3], Shannon Boi[3], Kah Teong Soh[8], John Chamberland[3,8], Xin Song[8], Yu Qin[3,8], Ilya Mishchenko[8], Maurice Kirby[8], Valeriia Nasonenko[8], Alexa Buffa[3,8], Jennifer S. Buell[3], Dhan Chand[8], Marc van Dijk[3], Justin Stebbing[9*¥], Mark A. Exley[10¥]

1. Pulmonary Critical Care Sleep Medicine, Providence Saint John's Health Center, Santa Monica, CA, USA
2. David Geffen School of Medicine at UCLA, Los Angeles, CA, USA
3. MiNK Therapeutics, Lexington, MA, USA
4. Dana Farber Cancer Institute, Boston, MA, USA
5. UH Seidman Cancer Center, Cleveland, OH, USA
6. Weil Cornell Medicine, NY, NY, USA
7. Norton Cancer Center, Louisville, KY, USA
8. Agenus, Lexington, MA, USA
9. Anglia Ruskin University, Cambridge, UK
10. Brigham & Women's Hospital, Boston, USA

[ᵀ] These authors contributed equally. [¥] These authors jointly supervised this work.

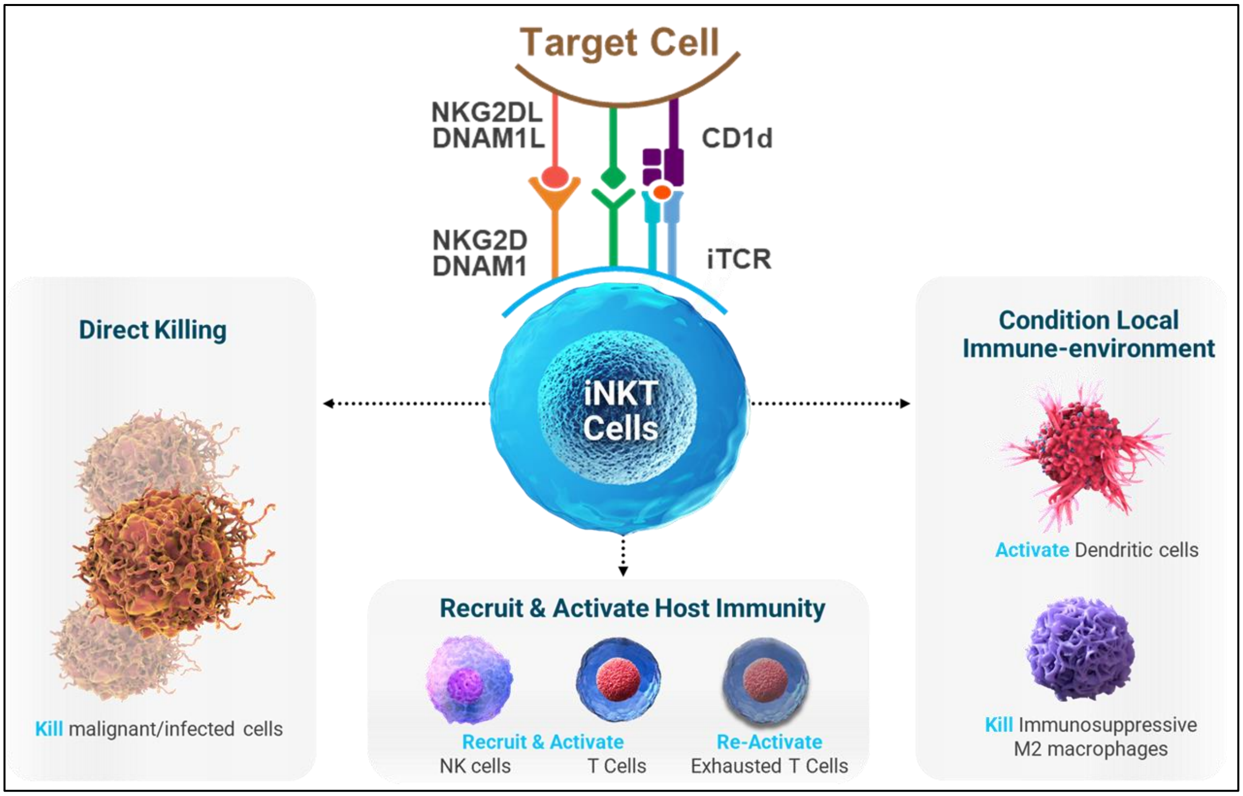


**Figure S1. Schematic mechanism of iNKT cell-induced functions.** iNKT cells recognize stressed and dying cells via the T cell antigen receptor (endogenous antigens presented on CD1d to the iTCR), NKG2D ligands (NKG2DL) and DNAM-1 ligands (DNAM-1L). iNKT cells can kill such target cells and release copious amounts of cytokines in response. Partly through such cytokines, stimulated iNKT cells also recruit and activate other immune cells, amplifying anti-pathogen effects. iNKT cells can also inhibit immuno-suppressive myeloid cells such as macrophages and myeloid-derived suppressor cells (MDSC), further enhancing anti-pathogen immunity.

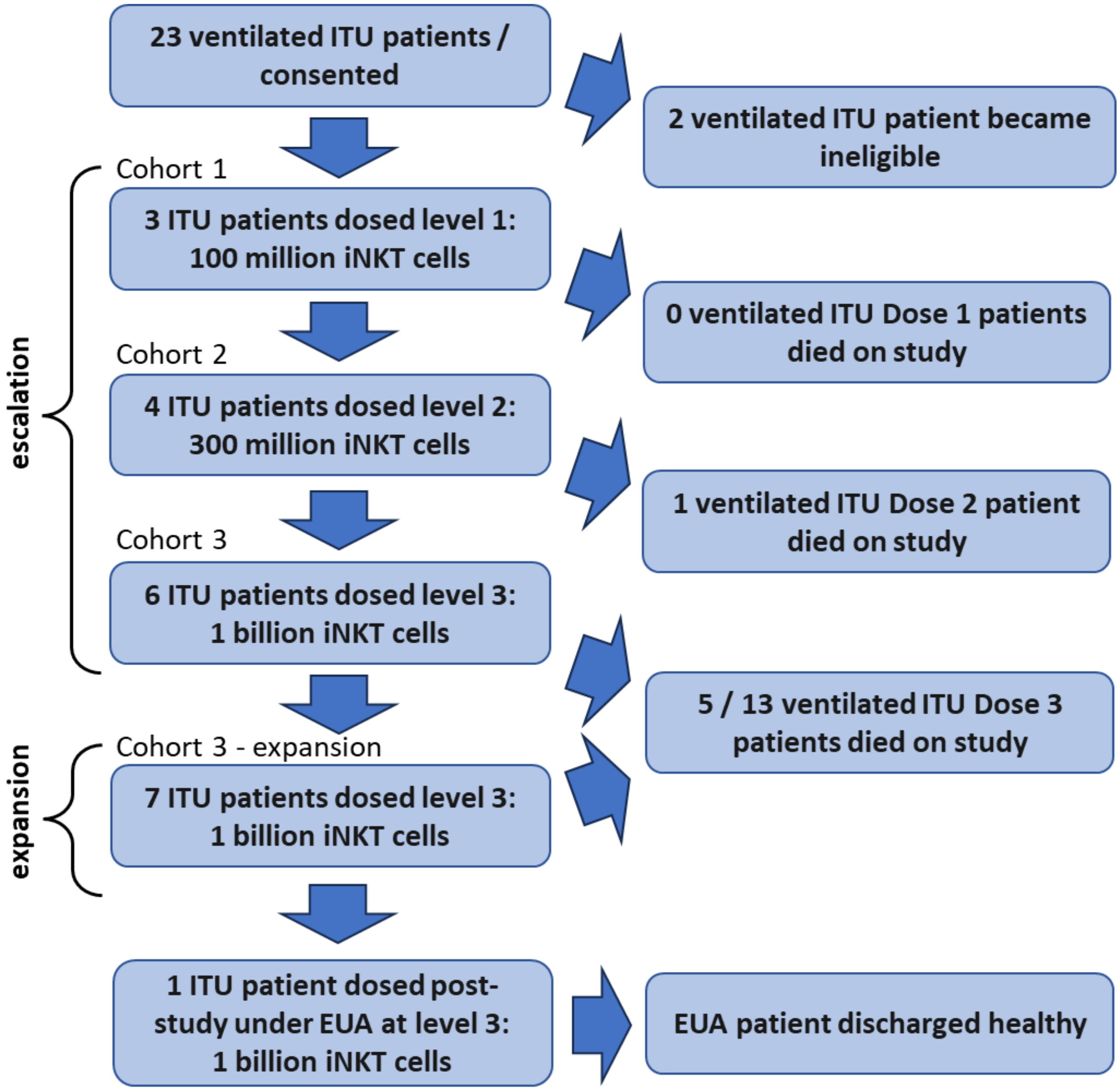


**Figure S2.** CONSORT Flow Diagram. 22 patients were initially enrolled, of which 2 patient became ineligible. 3 patients received Dose 1 of 100 million iNKT cells. Since only 1 grade 1-2 TRAE was noted, 3 patients received Dose 2 of 300 million. Since 1 of these had dyspnea of grade 3, a 4th patient received Dose 2 of 300 million with no grade 3+ TRAE. Subsequent patients received the maximum planned dose (Dose 3) of 1 billion iNKT cells. Since none of the first 3 patients enrolled at Dose 3 experienced DLT, an additional 3 patients were enrolled for a total of 6 patients at the maximum planned dose, with no DLT determined. All 7 remaining patients therefore received Dose 3 as dose-level expansion, with no further grade 3+ TRAEs observed. One additional patient was dosed outside the study under EUA at Dose 3. No ventilated ITU patients at any dose came off study post-treatment.

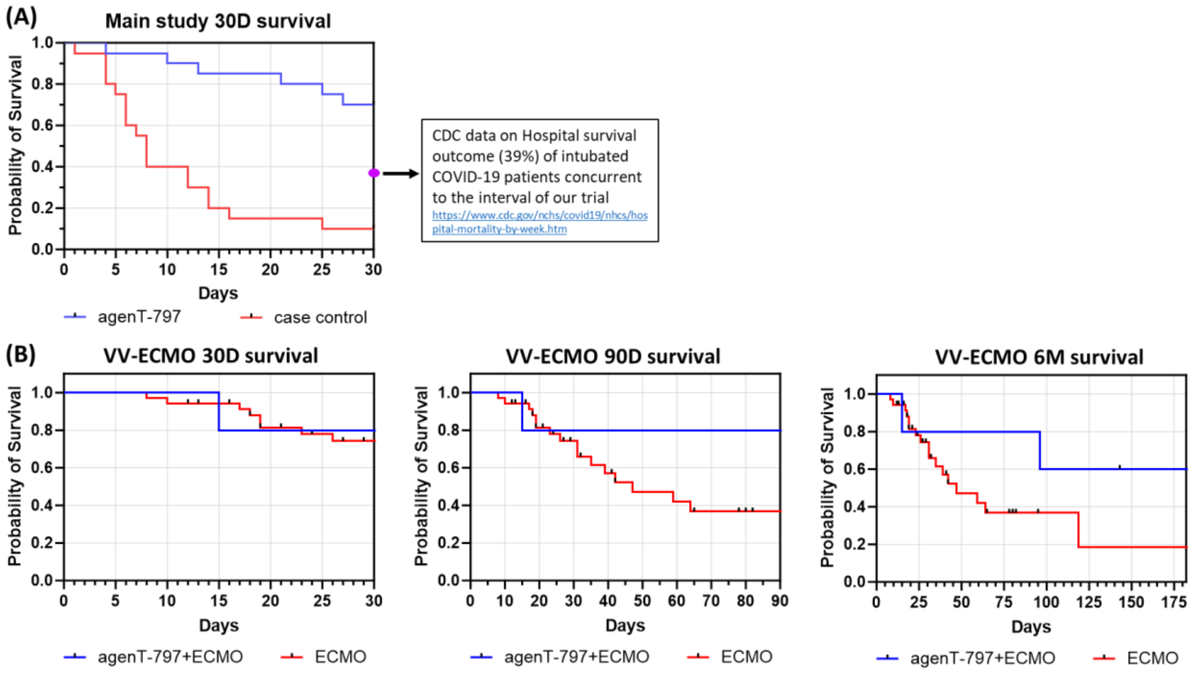


**Figure S3. On-study survival main study cohort and VV-ECMO subgroup. (S3A)** On-study (n=20) 30-day survival compared to control population survival outcomes. Blue line: Kaplan-Mayer survival curve demonstrating on-study survival of 70%. Red line: Survival outcome from a comparative control (n=20) evaluated at the same institution. **(S3B)** Survival of 4 main trial patients and EUA patient on vein-to-vein extracorporeal membrane oxygenation (VV ECMO) treated with agenT-797 (n=5; blue line). 30-day, 90-day, and 6-month survival of patients treated with agenT-797 (n=5). Control case data (n=36; red line) in ECMO-treated patients at the same investigator site.

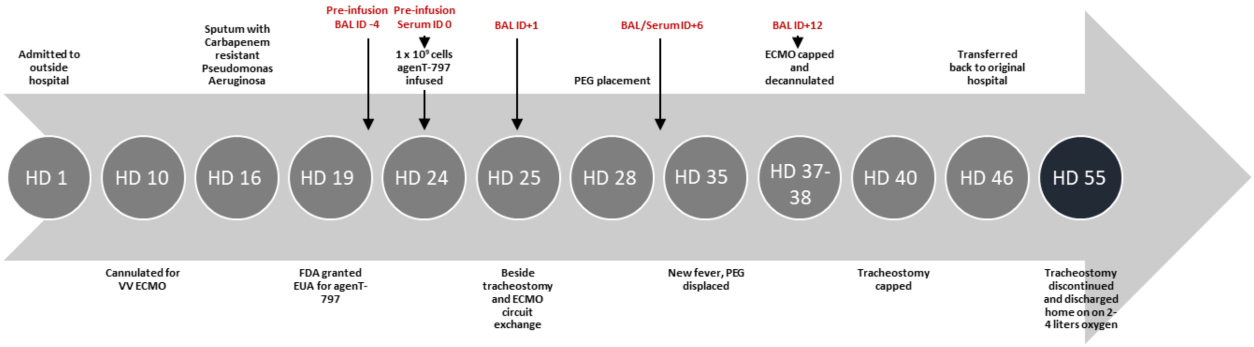


**Figure S4. Schematic of treatment for the individual EUA patient (IND 29183).** Time course of key events in the clinical course of the patient treated with agent-797 under EUA, from day of hospitalization (hospital day 1: HD 1) to discharge (HD 55). Timepoints of BAL and serum sample collection for cytokine analysis indicated in red text.

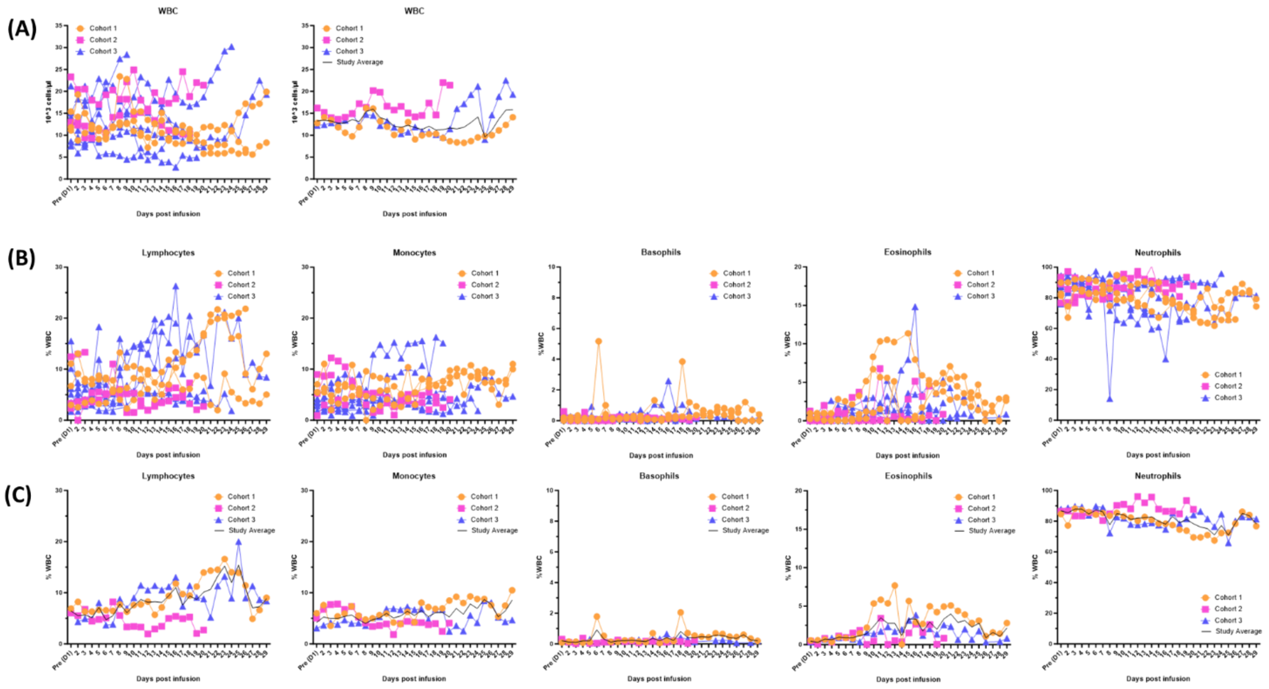


**Figure S5. Blood differential cell counts for dose level cohorts and individual patients. (S5A)** Absolute leukocyte counts (ALC) of white blood cells (WBC) for individual patients (left panel) and cohort/study averages (right panel). WBC subpopulations normalized as % of total WBC across individual patients **(S5B)** or averaged across cohorts/overall study **(S5C)** from Day 1 (measured pre-infusion on day of infusion as 'baseline' onwards). Note changes of scales between different WBC populations. Results are typical of the range for COVID-19 ITU patients with a variety of secondary infections (Table S3), as found in general in ITUs and particularly at this time of the pandemic29. Measurements from 13 patients (Cohort 1: n=3; Cohort 2: n=3; Cohort 3: n=7)

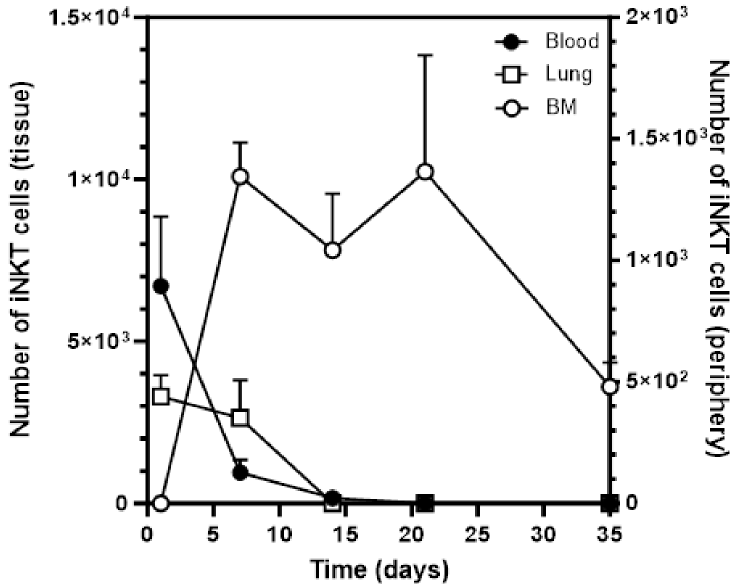


**Figure S6. In vivo tissue distribution of agenT-797.** Dynamics of tissue distribution of agenT-797 in a murine xenograft model showing translocation of agenT-797 to tissues. NOG mice expressing human IL-15 (NOG-hIL15) for the maintenance of human T lymphocytes were injected with 10 million agenT-797 cells and harvested at indicted timepoints (n=3 at each timepoint) to determine tissue prevalence of agenT-797. Graph shows absolute numbers of human iNKT cells detected in the periphery (solid symbol, right Y-axis) and within tissues (open symbol, left Y-axis). Data points represent mean with standard error (SEM).

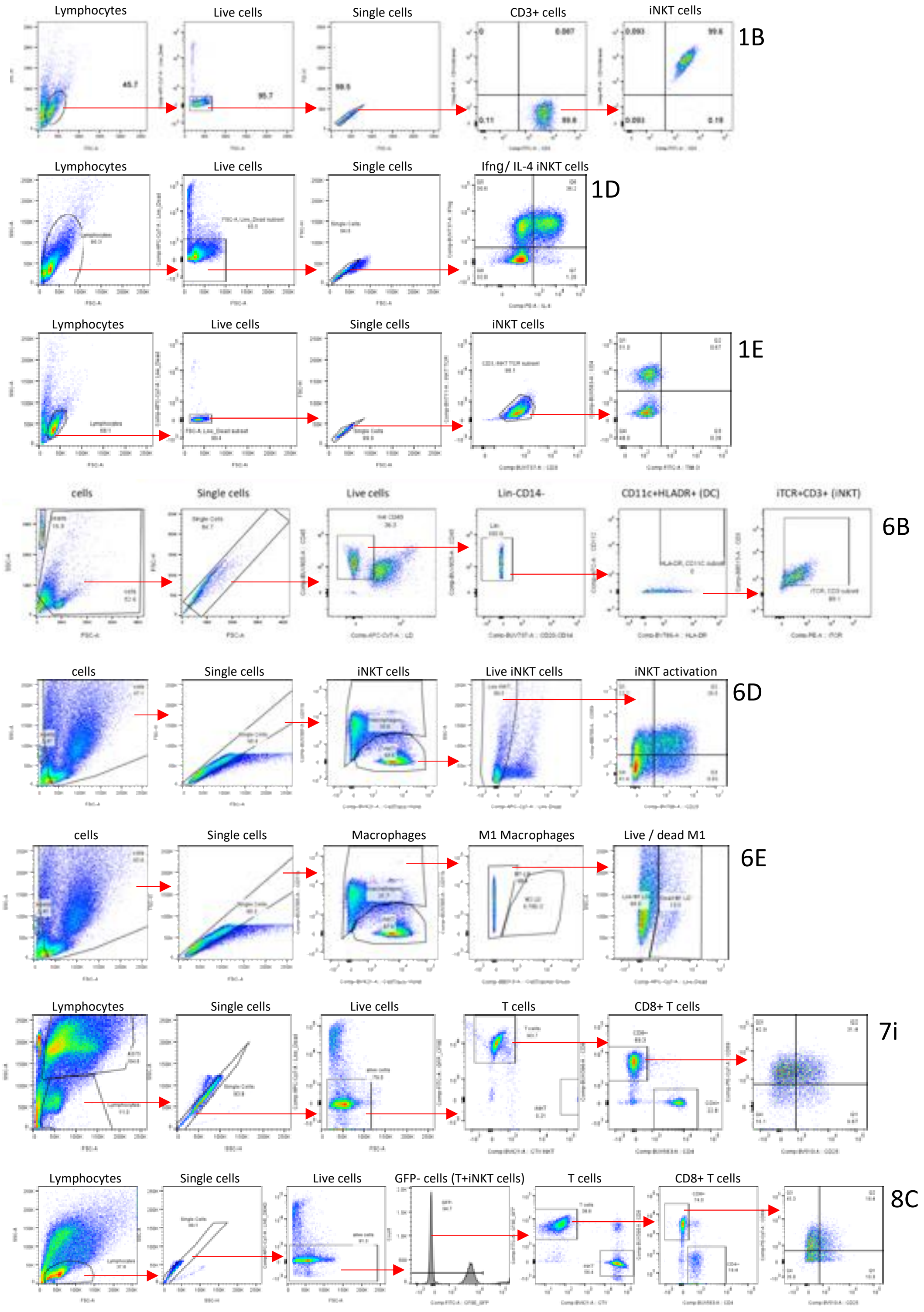


**Figure S7: FACS Gating strategies.** Graphic depiction of FACS gating strategies for each FACS data panel. The corresponding FACS data panels in the figures in the main manuscript are indicated to the left of each gating strategy graphic.

**Table S1.** Demographics and clinical overview of patients on VV-ECMO.

| **Variable** | **VV-ECMO + agenT-797** | **VV-ECMO control set*** |
|---|---|---|
| agenT-797 dose level (cells) | 1000 X $10^6$ | -- |
| Patients (n) | 5 | 35 |
| Age | | |
| *Median (range)* | 49 (21-69) | 43 (24-62) |
| Sex, n (%) | | |
| *Male* | 5 (100.0) | 30 (85.7%) |
| *Female* | 0 (0) | 5 (16.6%) |
| BMI, average (range) | 32.0 (23.7-39.6) | 30.8 (21.2-37.4) |
| Prior and concomitant COVID-19 medication, n (%) | | |
| *Received steroids (dexamethasone)* | 4 (80.0) | 35 (100%) |
| *Received immunomodulatory agents (tocilizumab, baricitinib)* | 2 (40.0) | 20 (57.1%) |
| *Received Remdesivir* | 4 (80.0) | 30 (85.7%) |
| Patient disposition, n (%) | | |
| *Death* | 2 (40.0) | 17 (48.6%) |

*Control set of COVID-19 patients on VV-ECMO from single site treated between 01/2021 and 01/2022

**Table S2.** Cohort level incidence of secondary infections.

| | | **Cohort 1 (n=3)** | **Cohort 2 (n=4)** | **Cohort 3 (n=13)** | **Overall (n=20)** |
|---|---|---|---|---|---|
| | Grade | n (%) | n (%) | n (%) | n (%) |
| Infections and infestations | | 3 (100.0) | 4 (100.0) | 6 (46.2) | 13 (65.0) |
| *Pneumonia* | 1-4 | 2 (66.7) | 3 (75.0) | 2 (15.4) | 7 (35.0) |
| *Bacteraemia* | 2-3 | 2 (66.7) | 0 | 1 (7.7) | 3 (15.0) |
| *Urinary tract infection* | 3 | 0 | 2 (50.0) | 1 (7.7) | 3 (15.0) |
| *Fungaemia* | 2-3 | 0 | 1 (25.0) | 1 (7.7) | 2 (10.0) |
| *Cytomegalovirus viraemia* | 3 | 0 | 0 | 1 (7.7) | 1 (5.0) |
| *Lung abscess* | 2 | 1 (33.3) | 0 | 0 | 1 (5.0) |
| *Pneumonia klebsiella* | 3 | 0 | 1 (25.0) | 0 | 1 (5.0) |
| *Sepsis* | 4 | 1 (33.3) | 0 | 0 | 1 (5.0) |
| *Septic shock* | 5 | 0 | 0 | 1 (7.7) | 1 (5.0) |
| *Upper respiratory tract infection* | 2 | 1 (33.3) | 0 | 0 | 1 (5.0) |

**Supplementary protocol file 1.** Trial protocol.

**Supplementary protocol file 2.** Statistical analysis plan.

**Supplementary protocol file 1.** Trial protocol.

**AGENT-797**

**C-1300-01**

# A PHASE 1/2 STUDY OF AGENT-797 TO TREAT MODERATE TO SEVERE ACUTE RESPIRATORY DISTRESS SYNDROME SECONDARY TO SARS-COV-2 OR INFLUENZA

| | |
|---|---|
| **Protocol number:** | C-1300-01 |
| **Phase:** | 1/2 |
| **Version, date:** | Version 9.0, 27 Apr 2022<br>incorporating Amendment 8 |
| **IND:** | 20757 |
| **Sponsor:** | MiNK Therapeutics, Inc.<br>3 Forbes Road<br>Lexington, MA 02421, USA<br>Telephone: +1 781-674-4686 |

**CONFIDENTIAL DOCUMENT**

This document is the confidential property of MiNK Therapeutics, Inc (MiNK). No part of it may be transmitted, reproduced, published, or used without the permission of MiNK.

*This study is to be conducted according to the protocol and in compliance with International Council for Harmonisation Good Clinical Practice (ICH GCP E6 [R2]) and other applicable regulatory requirements.*

# INVESTIGATOR'S AGREEMENT

No party involved in the conduct of this study will modify this protocol without obtaining MiNK's agreement. The party initiating a modification will confirm it in writing. MiNK will submit protocol amendments to the appropriate worldwide regulatory authorities. This study will be performed in accordance with appropriate federal or national regulations and International Council for Harmonisation Good Clinical Practice (ICH GCP E6 [R2]) guidelines.

Contact information will be provided to sites in the Investigator's Binder and site-specific documents. If necessary, contact the telephone numbers for specific adverse event reporting information in your country. The event must also be reported in writing within 24 hours of occurrence, within protocol-described timelines.

Institutional Review Board and/or Independent Ethics Committee must be notified of drug-related events according to the federal or national regulations.

I agree to conduct this study in accordance with this protocol and comply with all regulatory requirements as set forth in this protocol, appropriate federal or national regulations, and ICH GCP E6 (R2) guidelines. I also verify that I am the person responsible for the medical decisions at the site.

_______________________________________________

Printed Name of Investigator

_______________________________________________

Signature of Investigator

____________________________

Date

# PROCEDURES IN CASE OF EMERGENCY

**Table 1: Emergency Contact Information**

| Role in Study | Name | Email |
|---|---|---|
| Clinical Study Leader | Waldo Ortuzar Feliu, MD | Waldo.Ortuzar@Agenusbio.com |
| Responsible Physician | Koen Van Besien, MD, PhD | kov9001@med.cornell.edu |
| Drug Safety Representative | Anitha Swarna, PharmD | Anitha.Swarna@Agenusbio.com |
| 24-Hour Emergency Contact | Waldo Ortuzar Feliu, MD | Waldo.Ortuzar@Agenusbio.com |

# 1. SYNOPSIS

| | |
|---|---|
| **Name of Sponsor/Company:** MiNK Therapeutics, Inc. | |
| **Name of Investigational Product:** agenT-797 | |
| **Name of Active Ingredient:** agenT-797 | |
| **Title of Study:** A Phase 1/2 Study of agenT-797 to Treat Moderate to Severe Acute Respiratory Distress Syndrome Secondary to SARS-CoV-2 or Influenza | |
| **Study Centers:** Approximately 5 centers in the United States and Brazil | |
| **Principal Investigator:** Koen Van Besien, MD, PhD | |
| **Study Period:** Up to 12 weeks (intervention period) and up to 6 months of follow-up<br>Date of first patient enrolled: September 2020<br>Estimated date last patient completed: 2nd Quarter 2022 | |
| **Phase of Development:** 1/2 | |
| **Objectives and Endpoints for agenT-797 Assessment:** | |
| **Primary Objectives** | **Primary Endpoints** |
| • To assess safety and tolerability of agenT-797 infusion | • Number and severity of adverse events (AEs) and dose-limiting toxicities (DLTs) at evaluated dose levels |
| **Secondary Objectives** | **Secondary Endpoints** |
| • To assess evidence of improvement and resolution of acute respiratory distress syndrome (ARDS) following infusion with agenT-797<br>• To assess avoidance of multiorgan dysfunction syndrome | • Assessment of ventilator-free days<br>• Time to extubation<br>• Time to intubation in patients with moderate ARDS<br>• Vital signs and physical examination findings<br>• Lung injury score (LIS): The LIS is a composite 4-point scoring system including the $PaO_2/FiO_2$, positive end-expiratory pressure (PEEP), quasi-static respiratory compliance, and the extent of infiltrates determined by imaging.<br>• Intensive care unit–free days<br>• Mean daily sequential organ failure assessment score(s)<br>• Supportive interventions used (e.g., use of prone ventilation, paralytics, pulmonary vasodilators, and other interventions affecting oxygenation)<br>• All-cause mortality rates within 30 days and at 6 months |

| | |
|---|---|
| • To assess evolution of cytokine release syndrome (CRS) | • Change in clinical parameters and biomarkers relevant to CRS (D-dimers, ferritin, C-reactive protein, interleukin-1 (IL-1), and IL-6 |
| • To assess decay in quantitative (as feasible, or positive/negative) viral burden from upper and lower respiratory tract samples collected sequentially, as appropriate<br>• To assess prevention of viral reactivation | • Decay in quantitative (as feasible, or positive/negative) viral burden from upper and lower respiratory tract samples collected sequentially under treatment<br>• Time from dosing to viral clearance and determining if invariant natural killer T (iNKT) cells prevent re-activation of other viruses (cytomegalovirus, human papillomavirus, herpes simplex virus, Epstein-Barr virus) and fungal infections |
| **Exploratory Objectives** | **Exploratory Endpoints** |
| • To identify biomarkers that could predict clinical activity against severe acute respiratory syndrome-coronavirus 2 (SARS-CoV-2) | • Additional cytokine signatures and laboratory values including but not limited to levels of interferon gamma, lactate dehydrogenase, tumor necrosis factor alpha, prothrombin time, partial thromboplastin time, and creatine phosphokinase, change with treatment, and if change is correlated with clinical improvement |
| • To evaluate persistence and longevity of allogeneic iNKT cells agenT-797 with respect to tissue localization in target organs | • Presence and number of iNKT cells and other immune cells in bronchoalveolar lavage and/or microlavage fluid<br>• Persistence of allogeneic iNKT cells in circulation through direct measurement in peripheral blood and through cell free DNA as surrogate of general persistence in tissue(s) |
| • To evaluate development of alloimmunity against administered donor cells | • Presence of alloantibodies to major histocompatibility (MHC) Class I and MHC Class II and rate of mismatch |
| • To evaluate whether iNKT cells prevent secondary infections | • Presence of iNKT cells in peripheral blood and risk of secondary infections (e.g., *Pseudomonas aeruginosa*, *Klebsiella pneumoniae*, and fungal infections) |
| • To evaluate whether iNKT cells improve lung fitness through assessments of respiratory function | • Clinical and respiratory function assessments<br>• Change in pulmonary involvement based on X-ray and/or computed tomography (CT) scans, as available<br>• Health of lung epithelial cells (RNA analysis) |

| | |
|---|---|
| • To explore whether iNKT cells promote viral resolution without lung fibrosis in SARS-CoV-2 (COVID-19) patients | • Time from dosing to viral clearance<br>• Fibrosis signs based on X-ray and/or CT scans and respiratory function assessments |

**Methodology:**

This is a Phase 1/2 study to evaluate the safety and potential efficacy of agenT-797, an unmodified, allogeneic iNKT cell therapy, in patients with moderate to severe ARDS secondary to SARS-CoV-2 or influenza, either with intubation or at high risk to be intubated, as determined using Berlin definition(s) (ARDS 2012).

**Completed:** Part 1 will employ a standard 3+3 dose escalation design of agenT-797. All patients will receive a single infusion of agenT-797. Patients will also receive other treatments and supportive care per discretion of the Principal Investigator. Once the maximum tolerated dose of agenT-797 has been cleared in Part 1, an Expansion Cohort will be opened. A Safety Monitoring Committee (SMC) will be established to assess safety and decide on escalation to next cohort and expansion dose, as well as any protocol modification to include less severe cases.

**Part 2 (Expansion)** of the study will evaluate an additional cohort in up to 15 patients:

- Cohort 4 – patients with moderate to severe ARDS secondary to SARS-CoV-2 or influenza, according to Berlin definition, who are on mechanical intubation

The SMC will continue to review safety.

**Screening:**

Prior to any Screening activities, the patient (or legal representative) must sign an informed consent form and will proceed to undergo full screening procedures to determine eligibility. Full human leukocyte antigen-typing (blood) will also be performed.

Eligible patients will be treated with agenT-797 on Day 1. Assessments will be obtained through Day 32 or until discharge from hospital. Safety follow-up will occur 30 days after discharge, the patient to then be contacted regarding serious adverse events (SAEs) and to confirm survival up to 6 months after hospitalization.

**Number of patients (planned):** Up to 43 patients will be enrolled.

**Criteria for Study:**

**Inclusion Criteria:**

For inclusion in this trial, all the following inclusion criteria must be fulfilled. No waivers to inclusion criteria will be permitted.

1. Voluntarily agree to participate and can provide informed consent or have a duly appointed health care proxy established which/who has the authority to consent on behalf of the patient
2. $\geq$ 18 years of age
3. Inpatient hospitalization
4. Evidence of SARS-CoV-2 infection with the diagnosis of moderate to severe ARDS per Berlin definition (ARDS 2012), no more than 2 weeks prior to study enrollment
5. Patients at high risk of intubation or intubated with mechanical ventilation
6. Female patients of childbearing potential must be willing to use highly effective contraception measures starting with the Screening visit through 90 days after last dose of study treatment. Male patients with a female partner(s) of childbearing potential must agree to use highly effective contraceptive measures throughout the trial starting with the Screening visit through

90 days after the last dose of study treatment is received. Males with pregnant partners must agree to use a condom; no additional method of contraception is required for the pregnant partner

Note: Abstinence is acceptable if this is the established and preferred contraception method for the patient

7. Patients, or study participant's duly appointed health care proxy with the authority to consent on behalf of the patient, must consent to placement of a central venous access line for the administration of agenT-797

**Exclusion Criteria:**

For inclusion in the trial, patients must have none of the following exclusionary criteria. No waivers to exclusion criterion will be permitted:

1. Currently participating and receiving study therapy of an investigational agent that is not registered for any other indication
2. Life expectancy of < 1 month
3. Clinically significant cardiomyopathy
4. Pre-existing respiratory disease such as significant chronic obstructive pulmonary disease requiring home oxygen, hospitalization, or systemic steroid use during the past year.
5. "Significant" pulmonary hypertension as mean pulmonary artery pressure ≥ 20 mmHg and evidence of right ventricular dysfunction or enlargement
6. Receipt of vaccines containing live virus within 4 weeks prior to first dose of study treatment
7. Known hypersensitivity to donor-derived cell therapy or their preservation solution
8. Active systemic bacterial or fungal infection or viral co-infection with the exception of:
    a. Patients with rhinoviral infection (common cold) are permitted to participate
    b. Patients on treatment and/or ongoing improvement with minor common infections (e.g., urinary tract infection, minor skin infections) are permitted to participate
    c. Patients with both SARS-CoV-2 and influenza (co-infection) are permitted to participate
9. Pregnant or lactating women
10. **After implementation of Amendment 7**: Presence of multiorgan dysfunction syndrome; no organ failure should be seen other than the organ of interest, which is the lung

**Investigational Product, Dosage, and Mode of Administration:**

agenT-797 is an "off-the shelf" cell therapy consisting of ≥ 95% allogeneic human unmodified iNKT cells isolated from 1 healthy donor mononuclear cell apheresis unit and expanded ex-vivo.

- **Dosage Form:** Freshly thawed viably frozen agenT-797
- **Dosage and Cohorts:**
    - Cohort 1: $100 \times 10^6$ iNKT cells
    - Cohort 2: $300 \times 10^6$ iNKT cells
    - Cohort 3: $1000 \times 10^6$ iNKT cells
    - Cohort 4: $1000 \times 10^6$ iNKT cells

- **Dosage Frequency and Mode of Administration:** agenT-797 will be administered to hospitalized patients as a single intravenous infusion. The infusion time is approximately 10 to 30 minutes. Patients will be monitored for at least 4 hours postinfusion. In the event of reactions/toxicities during the infusion, the infusion rate may be modified, but the total infusion time should not exceed 60 minutes.

**Cohort Enrollment:**

- agenT-797 will be dosed 7 days after the dosing of the prior patient; dosing between cohorts will be initiated 14 days after the last patient has been dosed in the prior cohort (i.e., Cohort 2 will commence dosing 14 days after the last patient has been dosed in Cohort 1).

The following cohort is intended to be evaluated:

  – **Cohort 4:** up to 15 patients with moderate to severe ARDS secondary to SARS-CoV-2 or influenza, according to Berlin definition, who are on mechanical intubation

Note: Additional patients may be added at the discretion of the SMC based on the review of the totality of the data, including safety and efficacy data.

**Duration of Treatment**: One administration (single dose treatment with agenT-797).

**Criteria for Evaluation:**

**Safety:** Safety will be assessed by evaluating AEs graded according to National Cancer Institute Common Terminology Criteria for Adverse Events (NCI-CTCAE) Version 5.0: vital signs (blood pressure, heart rate, and temperature); physical examination, 12-lead electrocardiogram, and clinical laboratory assessments. Further, the SMC will assess safety, decide on dose escalation, and define the recommend dose for Part 2 (Dose Expansion).

**DLTs**: A minimum of 3 patients will be enrolled in each cohort, and the DLT observation period will be 14 days for each patient. If a DLT occurs in ≥ 2 patients in a cohort of 6 patients, the dose will not be escalated. In Amendment 7, SMC will monitor safety on an ongoing basis.

Patients will be examined for presence of alloantibodies to MHC Class I and II proteins and degree of human leukocyte antigen mismatch.

**Efficacy:**

- Intubation/continuation/discontinuation of intubation/mechanical ventilation
- Supportive oxygenation interventions
- Baseline and every 12 hours as feasible: C-reactive protein, D-dimers, ferritin, IL-1, IL-6 to assess CRS evolution
- Mortality
- Decrease in viral burden from upper respiratory tract samples collected sequentially under treatment
- Persistence of allogeneic iNKT cells
- Baseline and every 24 hours as feasible: complete blood count with differential, comprehensive metabolic panel, procalcitonin, B-type natriuretic peptide, fibrinogen, prothrombin time, partial thromboplastin time, magnesium, angiotensin II level
- Available image (X-ray, CT-scans) to evaluate respiratory involvement (ARDS)
- Change in lung fitness through assessment of respiratory function
- Time from dosing to viral clearance
- Prevention of secondary infections

**Duration of Study:**

Duration of the study is expected to be up to 2 years.

**Assessments:**

**Safety assessments**

Safety assessments will consist of monitoring nature, frequency, and severity of AEs as per NCI-CTCAE v5.0, laboratory data, vital signs, electrocardiograms, physical examinations, and clinical assessments.

**Clinical activity assessments**

Daily throughout hospitalization

Clinical evaluations will be recorded daily while the patient is hospitalized at the primary hospital facility. Additionally, selected evaluations at baseline (D1), + 24 hours (D2), + 72 hours (D4), + 5 days (D6), + 7 days (D8), + 10 days (D11), + 14 days (D15), + 21 days (D22), + 28 days (D29), D32 to End-of-Hospitalization, and at other days if significant event(s) occur. Once the patient has been discharged from the primary hospital facility, the patient will be contacted regarding SAEs and to confirm survival up to 6 months after hospitalization.

The following clinical evaluations will be conducted during specified intervals during hospitalization:

- Bronchoalveolar lavage: Evaluate iNKT cells and other immune cells in bronchoalveolar lavage fluid
- Posterior pharynx swabbing to assess inflammatory markers
- Nasal swabs will be performed to assess viral clearance
- Assessment of respiratory function

**Pharmacokinetic assessments**

Persistence of agenT-797 cells will be assessed at different timepoints as provided in the Schedule of Assessments.

**Pharmacodynamic and biomarker assessments**

Peripheral blood will be assessed for alloimmunization analysis and additional peripheral blood assessments may be conducted.

**Statistical Methods:**

**Sample size**

The total sample size is expected to be up to approximately 43 evaluable, treated patients. Since the study uses a 3+3 design and incorporates 3 dose levels, up to 18 patients may be needed in the Dose Escalation study component (Part 1). Up to 15 patients will be enrolled into Cohort 4 to further evaluate safety (Part 2, Expansion).

Refer to the statistical analysis plan (SAP) for details. A matched control may be used to complete the comparative analysis. This will be defined in the SAP.

**Analyses**

The SAP is being prepared and will include a more technical and detailed description of planned statistical summaries. The SAP will be finalized before initiating any statistical analyses. Unless otherwise stated, tabulation of summary statistics and data analysis will be performed using SAS Version 9.4 or later or R Version 6.0 or later.

This study is exploratory in nature; no formal statistical comparisons are planned.

# TABLE OF CONTENTS, LIST OF TABLES, AND LIST OF FIGURES

## TABLE OF CONTENTS

1. SYNOPSIS ....4
TABLE OF CONTENTS, LIST OF TABLES, AND LIST OF FIGURES ....10
LIST OF ABBREVIATIONS AND DEFINITIONS OF TERMS ....15
2. INTRODUCTION ....17
2.1. SARS-CoV-2 Infection ....17
2.2. Influenza ....18
2.3. agenT-797 ....19
2.4. Rationale for agenT-797 in Patients with Active SARS-CoV-2 Infection and Influenza ....19
2.4.1. Background ....19
2.4.2. Rationale for the Safe Use of iNKT Cells ....19
2.4.3. Built-in “Safety Switch” ....20
2.4.4. Corticosteroid Use and iNKT Cells ....20
2.5. Conclusions ....21
2.6. Starting Dose Rationale ....21
3. TRIAL OBJECTIVES AND ENDPOINTS ....22
4. INVESTIGATIONAL PLAN ....23
4.1. Overall Study Design ....23
4.2. Number of Patients ....24
4.3. Treatment Assignment ....24
4.4. Staggered Cohort Enrollment ....24
4.5. Dose Escalation Criteria ....25
4.5.1. Dose-Limiting Observation Period ....25
4.5.2. Maximum Tolerated Dose ....25
4.5.3. Safety Criteria for Adjustment or Stopping Doses ....25
4.5.4. Criteria for Study Termination ....26
4.5.5. Screening ....26
4.5.6. Treatment and Evaluation Period ....26

4.5.7. End-of-Hospitalization ....26
4.6. Study Procedures ....26
4.6.1. Administrative Procedures ....26
4.6.1.1. Informed Consent ....26
4.7. Study Criteria ....30
4.7.1. Patient Inclusion Criteria ....30
4.7.2. Patient Exclusion Criteria ....30
4.8. Patient Withdrawal from Study ....31
4.9. End-of-Hospitalization or Death ....31
4.10. 30-Day and Safety Follow-up ....31
4.11. Replacement of Patients ....31
4.12. Enrollment Stopping Rules ....32
4.13. Premature Stopping of Study ....32
4.14. Assignment of Patient Trial Number ....32
4.15. Trial Compliance ....32
4.16. Clinical Assessments and Procedures ....32
4.16.1. Medical History ....32
4.16.2. Review of Screening Procedures ....32
4.16.3. Review of Prior and Concomitant Medications ....33
4.16.3.1. Prior Medications ....33
4.16.3.2. Concomitant Medications ....33
4.16.4. Daily Assessments Throughout Hospitalization ....33
4.16.5. Review of Adverse Events ....33
4.16.5.1. Physical Examination, Vital Signs, Electrocardiograms, and Imaging ....33
4.16.6. Efficacy Assessments ....33
4.16.7. Pharmacokinetics, Immunogenicity, and Pharmacogenomics Assessments ....34
4.16.7.1. Laboratory Tests ....34
5. TREATMENT OF PATIENTS ....34
5.1. Description of Study Drug ....34
5.1.1. Manufacturing of agenT-797 ....34

5.1.2. Infusion of agenT-797 ....34
5.2. Supportive Care ....35
5.3. Prior and Concomitant Medication and Therapy ....35
5.3.1. Premedication with agenT-797 Infusion ....35
5.3.2. Recording of Concomitant Medication ....35
5.3.3. Prohibited Medications ....35
5.4. Risks Associated with agenT-797 Administration ....35
5.4.1. Possible Adverse Events Following Treatment with agenT-797 Infusion ....35
5.4.1.1. Cytokine Release Syndrome ....35
5.5. Treatment Compliance ....37
5.6. Randomization and Blinding ....37
6. STUDY DRUG MATERIALS AND MANAGEMENT ....37
6.1. Study Drug ....37
6.2. Study Drug Packaging and Labeling ....37
6.3. Study Drug Storage ....37
6.4. Study Drug Preparation ....37
6.4.1. Thawing of the Infusion Bag and Dilution ....37
6.5. Administration ....38
6.6. Study Drug Accountability and Disposal ....38
7. STUDY ASSESSMENTS ....38
8. ADVERSE AND SERIOUS ADVERSE EVENTS ....38
8.1. Definitions ....38
8.1.1. Adverse Event ....38
8.1.2. Serious Adverse Event ....38
8.1.3. AESIs ....39
8.1.4. Dose-Limiting Toxicity Criteria ....39
8.1.5. Recording and Reporting of Adverse Events ....40
8.1.6. Recording of Adverse Events/Serious Adverse Events ....40
8.1.7. Relationship ....40
8.1.8. Severity ....41

8.1.9. Pregnancy ....................................................................................................41
8.1.10. Reporting of Serious Adverse Events ....................................................................42
8.2. Safety Monitoring Committee ....................................................................................42
9. STATISTICS ..............................................................................................................43
9.1. Safety Analyses ..........................................................................................................43
9.2. Sample Size ................................................................................................................43
9.3. Criteria for the Termination of the Study ...................................................................43
9.4. Procedure for Accounting for Missing, Unused, and Spurious Data ..........................43
9.5. Deviations from the Original Planned Analysis .........................................................43
10. ETHICAL AND REGULATORY OBLIGATIONS .................................................44
10.1. Informed Consent .......................................................................................................44
10.2. Institutional Review Board/Independent Ethics Committee .......................................44
10.3. Study Documentation Requirements .........................................................................44
10.4. Patient Confidentiality ................................................................................................44
11. ADMINISTRATIVE AND LEGAL OBLIGATIONS ..............................................44
11.1. Protocol Amendments and Study Termination ..........................................................44
11.2. Data Handling and Recordkeeping .............................................................................45
11.3. Data Collection ...........................................................................................................45
11.3.1. Inspection of Records .............................................................................................45
11.3.2. Retention of Records ..............................................................................................45
11.3.3. Data Quality Assurance ..........................................................................................45
11.3.4. Study Monitoring ....................................................................................................45
11.3.5. Protocol Deviations ................................................................................................45
11.3.6. Audits and Inspections ...........................................................................................46
11.4. Publication Policy .......................................................................................................46
12. LIST OF REFERENCES .............................................................................................46
APPENDIX 1. PROTOCOL APPROVAL AND SIGNATURE PAGE .................................49
APPENDIX 2. ACUTE RESPIRATORY DISTRESS SYNDROME, BERLIN DEFINTION.50

# LIST OF TABLES

Table 1: Emergency Contact Information....3
Table 2: Cohorts for agenT-797....24
Table 3: Schedule of Assessments....27
Table 4: ASTCT CRS Consensus Grading....36

# LIST OF ABBREVIATIONS AND DEFINITIONS OF TERMS

The following abbreviations and specialist terms are used in this study protocol.

| Abbreviation or Specialist Term | Explanation |
|---|---|
| AE | adverse event |
| AESI | adverse events of special interest |
| ARDS | acute respiratory distress syndrome |
| ASTCT | American Society for Transplantation and Cellular Therapy |
| CAR-T | chimeric antigen receptor T cells |
| CDC | Centers for Disease Control and Prevention |
| COVID-19 | coronavirus disease of 2019 |
| CRF | case report form |
| CRS | cytokine release syndrome |
| CT | computed tomography |
| DLT | dose-limiting toxicity |
| EOH | End-of-Hospitalization |
| GCP | Good Clinical Practice |
| GMP | Good Manufacturing Practice |
| GVHD | graft versus host disease |
| ICF | informed consent form |
| ICH | International Council for Harmonisation |
| IEC | Independent Ethics Committee |
| IL | interleukin |
| iNKT cells | invariant natural killer T cells |
| IRB | Institutional Review Board |
| IV | intravenous |
| LIS | lung injury score |
| MDSC | myeloid-derived suppressor cells |
| MHC | major histocompatibility complex |
| MTD | maximum tolerated dose |
| NCI-CTCAE | National Cancer Institute Common Terminology Criteria for Adverse Events |
| NK | natural killer |
| PEEP | positive end-expiratory pressure |

| Abbreviation or Specialist Term | Explanation |
|---|---|
| SAE | serious adverse event |
| SAP | statistical analysis plan |
| SARS-CoV-2 | severe acute respiratory syndrome coronavirus 2 |
| SMC | Safety Monitoring Committee |

# 2. INTRODUCTION

## 2.1. SARS-CoV-2 Infection

In December 2019, a novel coronavirus, designated severe acute respiratory syndrome coronavirus 2 (SARS-CoV-2), caused an international outbreak of respiratory illness termed coronavirus disease of 2019 (COVID-19). The full spectrum of SARS-CoV-2 infection ranges from mild, self-limiting respiratory tract illness, to severe presentations of acute respiratory distress syndrome (ARDS), multiorgan failure, and death (Huang 2020, Liu 2020, Wang and Hu 2020).

Current treatment options for SARS-CoV-2 infections are limited. Signals of clinical benefit, including modest improvement in mortality or shorter recovery time, have been reported with some agents, such as high dose steroids or antiviral therapies such as remdesivir. In ARDS, these different treatments have been implemented as standard of care.

Most recently, a clinical study (the UK RECOVERY) demonstrated increased survival in a subset of COVID-19 patients treated with dexamethasone. In patients receiving respiratory support (mechanical ventilation or supplemental oxygen), the use of dexamethasone resulted in a decrease in 28-day mortality; however, a corresponding decrease in mortality was not observed in patients who did not receive respiratory support (RECOVERY 2020). An important observation from the RECOVERY trial was that dexamethasone provided benefit only to severely ill patients with COVID-19, suggesting that the immunomodulatory effects of glucocorticoids are beneficial in the hyperinflammatory phase of COVID-19, perhaps by breaking the inflammatory feedforward loop in some patients (Bowles 2002, Cain 2020).

Two recent clinical trials with remdesivir demonstrated a shorter time to recovery from COVID-19, when compared with placebo (Beigel 2020, Wang and Zhang 2020). In one of these studies conducted in China, remdesivir was not associated with significant clinical benefits, but was associated with a numerical reduction in time to clinical improvement (21 days) in patients with severe disease who were treated earlier in their disease course. However, treatment effect attribution to study results remains unclear in that the study protocol permitted concomitant use of lopinavir-ritonavir, interferons, and corticosteroids (Wang and Zhang 2020).

Preliminary data from another clinical study (i.e., global ACTT-1) demonstrated significant shortening of time to recovery for patients with moderate to severe COVID-19 when treated with remdesivir (median, 11 days, as compared with 15 days; rate ratio for recovery). In the case of patients with severe disease (88.2%; defined as requiring mechanical ventilation and/or supplementary oxygen) who were randomized to the remdesivir arm, 55.4% were no longer hospitalized by Day 15 post-treatment (versus 45.7% of patients treated with placebo) (Beigel 2020).

While preliminary findings support the use of remdesivir for patients who are hospitalized with COVID-19 and require supplemental oxygen therapy, the authors noted that given high mortality despite the use of remdesivir, it is clear that treatment with an antiviral drug alone is not likely to be sufficient to reduce mortality (Beigel 2020).

## 2.2. INFLUENZA

Influenza is an acute respiratory illness that occurs in outbreaks and epidemics worldwide, mainly during the winter season. It occurs in epidemics nearly every year and is remarkable for its high mutation rate, which compromises the ability of immune system to protect against new variants. Hence, new vaccines are being produced each year to match circulating variants. According to Centers for Disease Control and Prevention (CDC) vaccine effectiveness has ranged from 29% to 39% during recent years.

Influenza is a self-limited infection in the general population; however, it is associated with increased morbidity and mortality in certain high-risk populations. According to CDC groups, at high risk to develop serious complications are children < 5 years, but especially < 2 years, adults ≥ 65 years of age, residents of nursing homes and long-term care facilities, non-Hispanic black persons, Hispanic or Latino persons, American Indian or Alaska Native persons, and pregnant women.

The clinical presentation of influenza ranges between mild to severe depending on the age, comorbidities, vaccination status, and natural immunity to the virus (Boktor 2021). Influenza typically causes an acute infection of the upper respiratory tract. However, in severe cases, influenza can cause an infection of the lower respiratory tract, resulting in viral pneumonia and ARDS (Chowell 2009).

Although there are multiple risk factors for developing ARDS (bacteremia, sepsis, trauma, fractures, burns, massive transfusion, pneumonia, aspiration, etc.), pre–COVID-19, influenza A was the predominant viral etiology of ARDS and associated with high morbidity and mortality in adult population in the United States. According to World Health Organization, seasonal influenza may result in 290,000 to 650,000 deaths worldwide annually. No randomized trials have assessed mortality because all such trials have been conducted in healthy individuals in whom the mortality rate from influenza is very low.

Although death rates from influenza are usually disproportionately higher among older adult individuals and infants during influenza epidemics, a shift in the age distribution is seen during pandemics. During pandemics, higher rates of mortality occur not only at the extremes of age but also in young adults (Simonsen 1998, Taubenberger 2006).

Antiviral drugs, neuraminidase inhibitors (oseltamivir, peramivir, and zanamivir), and the cap-dependent endonuclease inhibitor, baloxavir marboxil (for treatment only), indicated for the treatment and chemoprophylaxis, can modify the severity of illness and reduce the duration by about 1.5 to 2.5 days and are being also used for prevention with efficacy range from 60% to 90% (Lehnert 2016). However, despite progress in pharmacology and vaccinology, influenza still affects morbidity and mortality in all age groups around the world.

Relative to the preceding, observational studies have identified an association between oseltamivir use and mortality reduction in patients with influenza (McGeer 2007, Bowles 2002, Muthuri 2014). No randomized trials have assessed mortality because all such trials have been conducted in healthy individuals in whom the mortality rate from influenza is very low.

In a cohort study conducted over 8 influenza seasons, 1330 critically ill patients were treated with oseltamivir for influenza infection, of whom 622 (47%) died in the intensive care unit. Among patients with influenza A H3N2, early treatment with oseltamivir (≤ 48 hours from symptom onset) was associated with lower mortalities (relative risk 0.69, 95% credible interval 0.49-0.94) compared with later initiation of treatment. No effect on mortality was observed among patients with influenza A H1N1 or influenza B infection.

## 2.3. AGENT-797

agenT-797 is composed of allogeneic human unmodified invariant natural killer T cells (iNKT cells) isolated from mononuclear cell apheresis units of healthy human donors. iNKT cells are a subclass of natural killer T lymphocytes found in the peripheral blood and organs. iNKT cells recognize lipid antigens (e.g., alpha-galactosylceramide) presented by CD1d molecules via invariant T cell receptors and respond by exerting their inherent immunomodulatory function. agenT-797 is intended for adoptive cell therapy and is initially being evaluated as a treatment for acute SARS-CoV-2 infection.

## 2.4. RATIONALE FOR AGENT-797 IN PATIENTS WITH ACTIVE SARS-COV-2 INFECTION AND INFLUENZA

### 2.4.1. Background

Severe complications of SARS-CoV-2 infection are characterized by lung hyperinflammation that can cause a life-threatening respiratory disorder associated with high viral load in epithelial cells, as well as other organ failures. The Sponsor is developing an allogeneic cell therapy product, agenT-797, that can promote antiviral activity and control of inflammation in this setting.

agenT-797 is an “off-the-shelf” readily injectable cell therapy product produced using donor apheresis material. A single manufacturing run from single donor apheresis can produce over 100 doses of agenT-797 in less than 30 days, with the number of doses being scalable with increased manufacturing of agenT-797.

### 2.4.2. Rationale for the Safe Use of iNKT Cells

iNKT cells are efficient orchestrators of the innate and adaptive immune responses and play an important role in the response to viral infections. Inherent immunomodulatory functions of iNKT cells may, therefore, help resolve mild to severe influenza infection by promoting viral clearance and by the dampening of exacerbated inflammation, thus preventing or controlling tissue damage. Elements associated with iNKT cell activity include, but are not limited to the following:

- Activation of iNKT cells enhances innate immune response(s) and improves the disease course in viral influenza virus. Influenza leads to the expansion of the myeloid-derived suppressor cells (MDSC) population; iNKT cells have demonstrated the ability to reduce

both the expansion of the MDSCs and the suppressive effect of MDSCs to improve influenza-specific responses (Juno 2012).

- iNKT cells can also reduce accumulation of inflammatory monocytes in the lungs and decrease immune-pathology during severe influenza virus infection. Lack of iNKT cells in influenza A infected mice have been shown to result in increased levels of inflammatory monocytes, which was correlated with increased lung injury and mortality. Activation of iNKT cells has also been shown correlate with reduced monocyte chemoattractant protein-1 levels and improved patient outcomes (Kok 2012).
- The Sponsor's iNKT data demonstrate that cells, upon infusion, exit the vasculature and accumulate within various tissues. In a healthy murine xenograft model, $1 \times 10^7$ agenT-797 cells were injected and were rapidly cleared from circulation, with cells having been shown to enter bone marrow and other tissues. Interesting iNKT cells remained detectable and present in bone marrow for > 35 days in this murine xenograft model.

### 2.4.3. Built-in "Safety Switch"

iNKT cell-based approaches have already been shown to have a reasonable safety profile (Kunii 2009, Motohashi 2006, Yamasaki 2011), even when iNKT cells are administered in the chronic viral infection setting (hepatitis B virus, hepatitis C virus trials; Field 2017).

### 2.4.4. Corticosteroid Use and iNKT Cells

Based on recently published data (RECOVERY 2020), high-dose steroid treatment (dexamethasone) is now considered standard of care for patients with the severe COVID-19 infection.

It is unknown whether use of steroids, or other agents with immunosuppressive activity, could impact the immunostimulatory effect of iNKT cells.

However, in patients with later-stage COVID-19, where immunopathology drives disease, the immunosuppressive benefits of dexamethasone on pro-inflammatory cytokines is complementary, not contraindicated for combination use with iNKT cell therapy. Glucocorticoids have been investigated in preclinical and clinical settings of coronaviruses and have most recently demonstrated reductions in the mortality of patients on ventilators. The underlying benefit attributed to the potent anti-inflammatory action of glucocorticoids is through inhibition of pro-inflammatory cytokines (interleukin [IL]-1, tumor necrosis factor, and IL-6), chemokines, and other soluble mediators (such as prostaglandin E2, leukotrienes and histamine) that act on the vasculature to promote vasodilation and leukocyte recruitment (Bowles 2002, Cain 2020). Treatment with agenT-797 (iNKT cells) may augment known anti-inflammatory effects of glucocorticoids by further reducing levels of IL-1 and IL-6 (Hu 2009).

While there are limited data related to the impact of glucocorticoids on immunologic mechanisms in the development of COVID-19 pathology(ies), data suggest that dexamethasone in combination with lenalidomide in patients with multiple myeloma may be used safely, in combination with immune therapies, and have no deleterious impact on iNKT cells. Importantly,

patients with multiple myeloma treated with lenalidomide and dexamethasone showed an increase in natural killer cell frequency (Lee and Lim 2016). In addition, steroids have been used in combination with chimeric antigen receptor T cells (CAR-T) therapy, with no differences observed relative to CAR-T proliferation/expansion in either high cumulative dose or low cumulative dose steroid administration settings (Topp 2019).

## 2.5. CONCLUSIONS

iNKT cells have natural antiviral roles and are known to traffic to the lungs. Their potential for improving both short-term and long-term immunity provide a compelling rationale for their exploitation in SARS-CoV-2 and influenza.

## 2.6. STARTING DOSE RATIONALE

In this study, an administered dose range of 100 to 1000 × $10^6$ iNKT cells will be explored. In an autologous iNKT cell clinical trial, patients received 3 doses every 2 weeks of iNKT cells (range of 15 to 220 million cells per dose) with no significant toxicities (Exley 2017). In other allogeneic cell therapy trials with related cell populations such as NK and cytokine-induced killer cells (McGeer 2007, Miller 2005, Laport 2011), study dosing began with modest initial doses of 100,000 cells/kg, which then escalated to 20 million cells/kg and then to 100 million cells/kg (McGeer 2007, Miller 2005, Laport 2011). These ranges are consistent with the proposed dose levels for agenT-797.

Using highly pure iNKT cells (> 95%) at the 3 doses proposed, prospective toxicities are further minimized by keeping levels of trace contaminating T cells to a minimum (Fuchs 2012). Indeed, iNKT cells actively suppress graft-versus-host-disease (GVHD) while maintaining antitumor activity providing further rationale for safety and potential clinical activity (Mavers 2017). Taken together these findings suggest that, even with cell therapies capable of expansion in vivo, half-log dose escalation schemes provide a safe and supported low initial dose providing for more rapid escalation to potentially more efficacious doses, within the study.

# 3. TRIAL OBJECTIVES AND ENDPOINTS

| Primary Objectives | Primary Endpoints |
|---|---|
| • To assess safety and tolerability of agenT-797 infusion | • Number and severity of adverse events (AEs) and dose-limiting toxicities (DLTs) at evaluated dose levels |
| **Secondary Objectives** | **Secondary Endpoints** |
| • To assess evidence of improvement and resolution of ARDS following infusion with agenT-797<br>• To assess avoidance of multiorgan dysfunction syndrome | • Assessment of ventilator-free days<br>• Time to extubation<br>• Time to intubation in patients with moderate ARDS<br>• Vital signs and physical examination findings<br>• Lung injury score (LIS): The LIS is a composite 4-point scoring system including the $PaO_2/FiO_2$, positive end-expiratory pressure (PEEP), quasi-static respiratory compliance, and the extent of infiltrates determined by imaging<br>• Intensive care unit–free days<br>• Mean daily sequential organ failure assessment score(s)<br>• Supportive interventions used (e.g., use of prone ventilation, paralytics, pulmonary vasodilators, and other interventions affecting oxygenation)<br>• All-cause mortality rates within 30 days and at 6 months |
| • To assess evolution of cytokine release syndrome (CRS) | • Change in clinical parameters and biomarkers relevant to CRS (D-dimers, ferritin, C-reactive protein, IL-1, and IL-6) |
| • To assess decay in quantitative (as feasible, or positive/negative) viral burden from upper and lower respiratory tract samples collected sequentially, as appropriate<br>• To assess prevention of viral reactivation | • Decay in quantitative (as feasible, or positive/negative) viral burden from upper and lower respiratory tract samples collected sequentially under treatment<br>• Time from dosing to viral clearance and determining if iNKT cells prevent re-activation of other viruses (cytomegalovirus, human papillomavirus, herpes simplex virus, Epstein-Barr virus) and fungal infections |
| **Exploratory Objectives** | **Exploratory Endpoints** |
| • To identify biomarkers that could predict clinical activity against severe acute | • Additional cytokine signatures and laboratory values, including, but not limited to, levels of |

| | |
|---|---|
| respiratory syndrome-coronavirus 2 (SARS-CoV-2) | interferon gamma, lactate dehydrogenase, tumor necrosis factor alpha, prothrombin time, partial thromboplastin time, and creatine phosphokinase, change with treatment, and if change is correlated with clinical improvement |
| • To evaluate persistence and longevity of allogeneic iNKT cells agenT-797 with respect to tissue localization in target organs | • Presence and number of iNKT cells and other immune cells in bronchoalveolar lavage and/or microlavage fluid<br>• Persistence of allogeneic iNKT cells in circulation through direct measurement in peripheral blood and through cell free DNA as surrogate of general persistence in tissue(s) |
| • To evaluate development of alloimmunity against administered donor cells | • Presence of alloantibodies to major histocompatibility (MHC) Class I and MHC Class II and rate of mismatch |
| • To evaluate whether iNKT cells prevent secondary infections | • Presence of iNKTs cells in peripheral blood and risk of secondary infections (e.g., *Pseudomonas aeruginosa, Klebsiella pneumoniae*, and fungal infections) |
| • To evaluate whether iNKT cells improve lung fitness through assessments of respiratory function | • Clinical and respiratory function assessments<br>• Change in pulmonary involvement based on X-ray and/or computed tomography (CT) scans, as available<br>• Health of lung epithelial cells (RNA analysis) |
| • To explore whether iNKT cells promote viral resolution without lung fibrosis in SARS-CoV-2 (COVID-19) patients | • Time from dosing to viral clearance<br>• Fibrosis signs based on X-ray and/or CT scans and respiratory function assessments |

# 4. INVESTIGATIONAL PLAN

## 4.1. Overall Study Design

This is a Phase 1/2 study to evaluate the safety and potential efficacy of agenT-797, an unmodified, allogeneic iNKT cell therapy, in patients with moderate to severe ARDS secondary to SARS-CoV-2 or influenza, either with intubation or at high risk to be intubated, as determined by the Berlin definition (ARDS 2012).

**Completed:** Part 1 will employ a standard 3+3 dose escalation design of agenT-797. All patients will receive a single infusion of agenT-797. Patients will also receive other treatments and supportive care per discretion of the Principal Investigator. Once the maximum tolerated dose (MTD) of agenT-797 has been cleared in Part 1, an Expansion Cohort will be opened. A Safety Monitoring Committee (SMC) will be established to assess safety and decide on escalation to

next cohort and expansion dose, as well as any protocol modification to include less severe cases.

**Part 2 (Expansion)** of the study will evaluate an additional cohort in up to 15 patients:

- Cohort 4 – patients with moderate to severe ARDS secondary to SARS-CoV-2 or influenza, according to Berlin definition, who are on mechanical intubation

The SMC will continue to review safety.

The number of patients in each cohort is presented in Table 2.

**Table 2: Cohorts for agenT-797**

| Cohort | Number of Patients | Escalation Type | agenT-797 level |
|---|---|---|---|
| 1 | 3-6 | 3+3 | $100 \times 10^6$ cells |
| 2 | 3-6 | 3+3 | $300 \times 10^6$ cells |
| 3 | 6+ | 3+3 | $1000 \times 10^6$ cells |
| 4 | Up to 15 | 3+12 | $1000 \times 10^6$ cells |

## 4.2. Number of Patients

It is estimated that up to approximately 43 patients, requiring or at risk of needing mechanical ventilation with moderate to severe ARDS, per the Berlin definition (ARDS 2012), will be enrolled into the study. Additional patients may be added at the discretion of the SMC based on the review of the totality of the data including safety and efficacy data.

## 4.3. Treatment Assignment

This is an open-label, single-arm study. Patients will receive a single dose of agenT-797. Each patient will stay on the dose level and schedule assigned at trial entry. No randomization and blinding procedures will be applied to treatment assignments.

## 4.4. Staggered Cohort Enrollment

Each cohort will have a minimum of 3 patients enrolled. agenT-797 (Cohort 1) will be administered in the first patient, and treatment will be administered to the next patient 7 days after the last patient was administered agenT-797. Cohort 2 will be administered 14 days after the last patient was dosed in Cohort 1 and will follow the staggered dosing in Cohort 1 (every 7 days/patient). If patients tolerate low dose (Cohort 1) and intermediate dose (Cohort 2) treatment with agenT-797, Cohort 3 may be enrolled after 14 days.

An additional cohort in up to 15 patients will be evaluated:

- Cohort 4 – patients with moderate to severe ARDS secondary to SARS-CoV-2 or influenza, according to Berlin definition, who are on mechanical intubation

## 4.5. Dose Escalation Criteria

A SMC will assess safety, decide on appropriateness of dose escalation, and define the expansion cohort dose.

### 4.5.1. Dose-Limiting Observation Period

A minimum of 3 patients will be enrolled in each cohort, and the DLT observation period will be 14 days for each patient. Patients will continue to be monitored post–DLT period throughout the study. The totality of the safety data will be factored in by the SMC in the decision-making to move to enrollment of Cohort 2.

If a DLT occurs in 1 of the 3 patients during the DLT period, the cohort will be expanded to a total of 6 patients; if a DLT occurs in ≥ 2 patients in the total cohort of 6 patients, the MTD will be deemed to be exceeded, and that dose will not be escalated. The prior dose level will be evaluated to then proceed into the Expansion Cohort. The interval between patients in each cohort is at least 7 days with a 14-day interval between each cohort (Section 4.4).

The dose of agenT-797 will be escalated if none of the first 3 evaluable patients enrolled has a DLT based on SMC evaluations of data. If a cohort was expanded to include a total of 6 evaluable patients and no DLT occurs in the additional 3 patients, then the dose will be escalated based on the SMC evaluation of data.

After Amendment 7, the SMC will monitor safety on an ongoing basis.

### 4.5.2. Maximum Tolerated Dose

If a DLT occurs in ≥ 2 patients in a cohort, the MTD will be deemed to be exceeded, and the prior dose level will be evaluated to determine the MTD by increasing enrollment to 6 patients. If the prior dose level was already deemed to be safe and enrolled 6 patients, then it will be defined as the MTD. Dose Escalation will continue until the MTD is reached or the maximum planned dose level is shown to be safe. If none of the first 3 evaluable patients enrolled at the maximum planned dose experience a DLT, an additional 3 patients may be enrolled for a total of 6 patients at the maximum planned dose. Additional patients may also be enrolled in other lower dose cohorts.

### 4.5.3. Safety Criteria for Adjustment or Stopping Doses

Enrollment and treatment will be temporarily stopped if any of the following occur:

- DLT(s) defined as follows: two Grade 4 DLTs or single Grade 4 neurological toxicity or single Grade 3 or higher GVHD as DLT
- Any toxicity that is unexpected, significant, and unacceptable (based on SMC review and discussion), e.g., any occurrence of GVHD Grade 3 or higher
- Any death that occurs within 30 days of agenT-797 administration, except cases reported as related to the primary disease (COVID-19 or influenza)

If any of the above stopping criteria are met, an SMC meeting will occur to review the information and to recommend how to proceed.

The SMC will review any cohort under evaluation as well as the safety of all cohorts. All toxicities, including those outside the 14-day DLT period, will be evaluated. The SMC will make recommendations regarding any changes in study conduct (Section 8.2).

### 4.5.4. Criteria for Study Termination

Study termination criteria are provided in Section 9.3. End-of-Hospitalization (EOH) details are provided in Section 4.5.7.

### 4.5.5. Screening

Written informed consent must be obtained prior to performing any protocol-specific Screening procedures to determine eligibility (Section 4.6.1.1 and Table 3). The Screening procedures may be conducted within 4 days prior to agenT-797 administration.

### 4.5.6. Treatment and Evaluation Period

Eligible patients will be treated with agenT-797 on Day 1. Assessments will be obtained through Day 32 or until discharge from hospital as outlined in the Schedule of Assessments in Table 3.

### 4.5.7. End-of-Hospitalization

A patient’s discharge from hospital or death will be considered EOH (whichever comes first). The patient will be contacted regarding any serious adverse event (SAEs) and to confirm survival up to 6 months after hospitalization (Section 4.9).

## 4.6. STUDY PROCEDURES

Study procedures and assessments are provided in Section 4.16.

### 4.6.1. Administrative Procedures

#### 4.6.1.1. Informed Consent

The Principal Investigator or qualified designee (a duly appointed health care proxy with authority to consent) must obtain documented informed consent from each patient, or legal representative, prior to participating in the clinical trial (Section 4.7.1). A signed and dated informed consent form (ICF) must be received and documented prior to initiation of any study procedures. The ICF will adhere to Institutional Review Board (IRB)/Independent Ethics Committee (IEC) requirements, applicable laws and regulations, and Sponsor requirements.

**Table 3: Schedule of Assessments**

| Assessment | Screening | Treatment Period | | | | | | | | | | EOH[a] | 30-Day F/U |
|---|---|---|---|---|---|---|---|---|---|---|---|---|---|
| **Study Day** | **± 4** | **1** | **2** | **4** | **6** | **7** | **10 ± 1** | **14 ± 1[b]** | **21 ± 2[b]** | **28 ± 2[b]** | **32 to EOH[b]** | **Day of discharge** | **30 ± 2** |
| Informed consent | X[c] | | | | | | | | | | | | |
| Inclusion/exclusion | X[c] | | | | | | | | | | | | |
| HLA serotype | X[c] | | | | | | | | | | | | |
| Medical history & demographics | X[c] | | | | | | | | | | | | |
| Physical examination[d] | X[c] | Daily monitoring/assessments | | | | | | | | | | X | X |
| Vital signs[e] | X[c] | Daily monitoring/assessments | | | | | | | | | | X | X |
| ECG[f] | X[c] | X | | | | | | | | X | | X | X |
| Radiological evaluation (X-ray/CT) | X[c] | X | | | | | | | | | | X | |
| Concomitant medications & procedures | X | Continuously collected | | | | | | | | | | | |
| Adverse events[g] | X | Continuously collected | | | | | | | | | | | |
| Hematology[h] | X[c] | Daily monitoring/assessments | | | | | | | | | | X | X |
| Coagulation[h] | X[c] | X | | X | | X | X | X | X | X | | X | X |
| Clinical chemistry[h] | X[c] | Daily monitoring/assessments | | | | | | | | | | X | X |
| Urinalysis[i] | X[c] | X | | X | | X | X | X | X | X | | X | X |
| Pregnancy test (WOCBP)[j] | X | | | | | | | | | | | | |
| Serum for cytokine analyses[k] | X[c] | X | | X | | X | X | X | X | X | | X | |

| **Assessment** | **Screening** | **Treatment Period** | | | | | | | | | | **EOH**[a] | **30-Day F/U** |
|---|---|---|---|---|---|---|---|---|---|---|---|---|---|
| **Study Day** | **± 4** | **1** | **2** | **4** | **6** | **7** | **10 ± 1** | **14 ± 1**[b] | **21 ± 2**[b] | **28 ± 2**[b] | **32 to EOH**[b] | **Day of discharge** | **30 ± 2** |
| Whole blood for persistence of agenT-797 (peripheral persistence) | X[c] | X[l] | X | X | X | X | X | X | X | X | | X | X |
| Whole blood for cfDNA (tissue persistence) | X[c] | X[m] | X | X | X | X | X | X | X | X | | X | X |
| Bronchoalveolar lavage (iNKT cell presence and biomarker)[k,n] | X[c] | X | | X | | X | X | X | X | X | | X | |
| Epithelial sampling by bronchial protected specimen brush (RNA analysis)[o] | X[c] | X | | X | | X | X | X | X | X | | X | |
| Serum for alloantibodies | | X[c] | | | | | | X | | | | X | |
| agenT-797 cell infusion | | X | | | | | | | | | | | |
| Arterial blood gas | X | Daily monitoring/assessments as needed | | | | | | | | | | X | X |
| Symptoms of ARDS[p] evaluation (Berlin definition)[q] | X | Daily monitoring/assessments as needed | | | | | | | | | | X | X |
| SOFA score[r] | X | Daily monitoring/assessments as needed | | | | | | | | | | X | |
| Viral burden assessment (swab), or endotracheal suction as feasible (RT-PCR) | X[s] | X | X | X | X | X | X | X | X | X | | X | |
| Viral shedding assessment as feasible | | X | | | | | | | | | | X | |
| D-dimer, CPK, cardiac troponins, and ferritin | | Every 12h, as feasible | | | | | | | | | | X | |

Abbreviations: AE: adverse events; AESI: adverse events of special interest; ARDS: acute respiratory distress syndrome; d: day(s); cfDNA: cell free DNA; CPK: creatine phosphokinase; CT: computed tomography; ECG: electrocardiogram; EOH: End-of-Hospitalization; F/U: Follow-up; HLA: human leukocyte antigen; h: hour (s); IL: interleukin; iNKT cells: invariant natural killer T cells; RT-PCR: reverse transcriptase polymerase chain reaction; SAE: serious adverse event; SOFA: sequential organ failure assessment; WOCBP: women of childbearing potential.

[a] End-of-Hospitalization is defined as patient discharge from hospital and/or death. If a patient is discharged at Day 30, 30-day Follow-up will be the same as EOH. After discharge, a patient will enter Safety Follow-up period and the patient will be contacted by telephone 3 and 6 months after discharge regarding any SAEs and to confirm survival, as feasible. See Sections 4.9 and 4.10.

[b] In case of earlier discharge from the hospital, no visit is expected until 30-Day Follow-up.

[c] Performed before infusion of agenT-797 on D1. These samples will be obtained at the time of the routine labs blood draws.

[d] A complete examination, as feasible, will be performed at Screening and symptom-directed and medically indicated physical examinations are done thereafter.

[e] Vital signs include body temperature, heart rate, respiratory rate, systolic and diastolic blood pressure and are assessed by physician or nurse. On the day of infusion of agenT-797 (D1), measurements will be 15 min prior to infusion, and 15 min, 30 min, 1h, 2h, 3h, and 4h postinfusion.

[f] ECG to be performed at Screening, Day 1 predose and EOH, 30-Day Follow-up, and at other times as clinically indicated.

[g] Only AEs associated with study procedures and/or leading to withdrawal and all SAEs will be collected prior to study drug infusion. Patients are monitored for toxicities following infusion. All other AEs, SAEs, and AESIs will be collected as per Section 8 and as defined in the Safety Follow-up period (Section 4.10).

[h] Hematology/coagulation/clinical chemistry: Complete blood count with automated and/or manual differential will be collected daily, as feasible. Coagulation: activated partial thromboplastin time and international normalized ratio. Clinical chemistry: includes but not limited to albumin, alkaline phosphatase, alanine aminotransferase, aspartate aminotransferase, blood urea nitrogen, urea, calcium, chloride, C-reactive protein, creatinine, glucose, lactate dehydrogenase, magnesium, phosphate/phosphorus, potassium, sodium, total bilirubin, direct and indirect bilirubin, total protein, and beta-2-microglobulin and will be collected daily, as feasible. All will be collected at EOH and at 30-Day Follow-up.

[i] Urinalysis: pH, ketones, specific gravity, bilirubin, protein, blood, and glucose. Will be done at the days indicated and at EOH and at 30-Day Follow-up.

[j] Pregnancy testing for women of child-bearing potential: serum test at Screening.

[k] Cytokines/biomarkers: D1 – preinfusion, end of infusion, and at 2, 4, and 8 hours postinfusion agenT-797, and as feasible thereafter. Cytokines for analysis may include but are not limited to: epidermal growth factor, eotaxin, fibroblast growth factor-basic, granulocyte-colony stimulating factor, granulocyte-macrophage colony stimulating factor, hepatocyte growth factor, interferon-alpha, interferon-gamma, IL-1 beta, IL-1 alpha, IL-1RA, IL-2, IL-2R, IL-3, IL-4, IL-5, IL-6, IL-7, IL-8, IL-9, IL-10, IL-12 (p40/p70) IL-13, IL-15, IL-17A, IL-17F, IL-22, IP-10, monocyte chemoattractant protein-1, MIG, MIP-1 alpha, MIP-1 beta, Regulated upon Activation, Normal T Cell Expressed and Presumably Secreted, tumor necrosis factor-alpha, vascular endothelial growth factor, and matrix metallopeptidase-9.

[l] To be obtained 5, 15, and 30 min, and 1, 2, and 4 hours postinfusion.

[m] To be obtained 2 hours postinfusion.

[n] To be obtained in intubated patients if clinically indicated using microlavage techniques when possible.

[o] To be obtained in intubated patients when possible.

[p] Evaluations including physical examination and procedures required for lung injury score evaluation (chest X-ray, $PaO_2/FiO_2$ ratio, positive end-expiratory pressure, compliance, mL/cm $H_2O$) as needed, including daily or more often until recovery (ARDS 2012).

[q] As needed.

[r] SOFA score will be calculated using results of the following tests: $PaO_2$, $FiO_2$, platelet count, need for mechanical ventilation, Glasgow Coma score, bilirubin, mean arterial pressure or use of vasoactive medications, creatinine, and diagnosis of COVID-19 (SOFA).

[s] Patients must be SARS-CoV-2 or influenza positive at diagnosis; refer to Section 4.7.1.

## 4.7. STUDY CRITERIA

### 4.7.1. Patient Inclusion Criteria

For inclusion in this trial, all the following inclusion criteria must be fulfilled. No waivers to inclusion criteria will be permitted.

1. Voluntarily agree to participate and can provide informed consent or have a duly appointed health care proxy established which/who has the authority to consent on behalf of the patient
2. ≥ 18 years of age
3. Inpatient hospitalization
4. Evidence of SARS-CoV-2 infection with the diagnosis of moderate to severe ARDS per Berlin definition (ARDS 2012), no more than 2 weeks prior to study enrollment
5. Patients at high risk of intubation or intubated with mechanical ventilation
6. Female patients of childbearing potential must be willing to use highly effective contraception measures starting with the Screening visit through 90 days after last dose of study treatment. Male patients with a female partner(s) of childbearing potential must agree to use highly effective contraceptive measures throughout the trial starting with the Screening visit through 90 days after the last dose of study treatment is received. Males with pregnant partners must agree to use a condom; no additional method of contraception is required for the pregnant partner

   Note: Abstinence is acceptable if this is the established and preferred contraception method for the patient
7. Patients, or study participant's duly appointed health care proxy with the authority to consent on behalf of the patient, must consent to placement of a central venous access line for the administration of agenT-797

### 4.7.2. Patient Exclusion Criteria

For inclusion in the trial, patients must have none of the following exclusionary criteria. No waivers to exclusion criteria will be permitted:

1. Currently participating and receiving study therapy of an investigational agent that is not registered for any other indication
2. Life expectancy of < 1 month
3. Clinically significant cardiomyopathy
4. Pre-existing respiratory disease, such as significant chronic obstructive pulmonary disease requiring home oxygen, hospitalization, or systemic steroid use during the past year.
5. "Significant" pulmonary hypertension as mean pulmonary artery pressure ≥ 20 mmHg and evidence of right ventricular dysfunction or enlargement

6. Receipt of vaccines containing live virus within 4 weeks prior to first dose of study treatment
7. Known hypersensitivity to donor-derived cell therapy or their preservation solution
8. Active systemic bacterial or fungal infection or viral co-infection with the exception of:
    a. Patients with rhinoviral infection (common cold) are permitted to participate
    b. Patients on treatment and/or ongoing improvement with minor common infections (e.g., urinary tract infection, minor skin infections) are permitted to participate
    c. Patients with both SARS-CoV-2 and influenza (co-infection) are permitted to participate
9. Pregnant or lactating women
10. **After implementation of Amendment 7:** Presence of multiorgan dysfunction syndrome; no organ failure should be seen other than the organ of interest, which is the lung

## 4.8. Patient Withdrawal from Study

Patients must be withdrawn from the study in the following situations:

- Severe noncompliance to this protocol as judged by the Investigator and/or Sponsor
- Pregnancy
- Withdrawal of consent
- Investigator discretion
- Sponsor decision

## 4.9. End-of-Hospitalization or Death

After the patient has received a single infusion of agenT-797, the EOH is defined as patient discharge from the hospital or death.

## 4.10. 30-Day and Safety Follow-up

The Safety Follow-up period will be 30 days, and the patient will be contacted by telephone 3 and 6 months after discharge regarding any SAEs and to confirm survival, as feasible. The first Safety Follow-up Visit will be at Day 30 (± 2), or at EOH. If a patient is discharged at Day 30, 30-day Follow-up will be the same as EOH. Please refer to Table 3.

## 4.11. Replacement of Patients

Patients are at any time free to withdraw from the study. Such patients will always be asked about the reason(s) and the presence of any AEs. AEs should be followed up as outlined in Section 8.1.5. The Investigator may also elect to discontinue patients from the study, for example, to pursue a different treatment option. Reason(s) for discontinuation will be documented.

Patients who are withdrawn from the study but have completed treatment (infusion of agenT-797), are to be considered evaluable for DLT, and will not be replaced. Any patient who is withdrawn and is not considered evaluable for safety during Dose Escalation will be replaced to ensure a minimum number of evaluable patients.

## 4.12. ENROLLMENT STOPPING RULES

Enrollment may be temporarily stopped if any of the following occur:

- A DLT (see Section 8.1.4)
- Any toxicity that is unexpected, significant, and unacceptable (based on SMC review; Section 8.2)
- Safety stopping rules (Section 4.5.3)
- Documented Sponsor decision

## 4.13. PREMATURE STOPPING OF STUDY

The study may be prematurely stopped due to the following reasons:

- The discovery of an unexpected, significant, or unacceptable risk to the patients enrolled in the study
- A decision on the part of the Sponsor to suspend or discontinue the study
- Any other reason as determined by the Sponsor or SMC

The Sponsor will be responsible for all decisions pertaining to prematurely discontinuing the study.

## 4.14. ASSIGNMENT OF PATIENT TRIAL NUMBER

After a patient signs an ICF, the patient will be assigned a unique, sequential patient number. Once a number is assigned, it cannot be reassigned if the original patient is found to be ineligible or withdraws consent.

## 4.15. TRIAL COMPLIANCE

Compliance is ensured, as patients will be hospitalized (Section 4.7.1). Patients who are discharged (EOH) will be followed per Section 4.5.7 and Section 4.10.

## 4.16. CLINICAL ASSESSMENTS AND PROCEDURES

### 4.16.1. Medical History

Medical history will be taken during the Screening period (Table 3).

### 4.16.2. Review of Screening Procedures

Screening procedures will be conducted as outlined in Table 3.

### 4.16.3. Review of Prior and Concomitant Medications

#### 4.16.3.1. Prior Medications

The following medications are not permitted:

- Receipt of vaccines containing live virus within 4 weeks prior to first dose of study treatment

#### 4.16.3.2. Concomitant Medications

All medications are recorded from the date of ICF. Also see Section 5.3.3 for prohibited concomitant medications.

### 4.16.4. Daily Assessments Throughout Hospitalization

Disease evaluations will be recorded daily while patient is hospitalized. Additionally, selected evaluations at baseline (D1), + 24 hours (D2), + 72 hours (D4), + 5 days (D6), + 7 days (D8), + 10 days (D11), + 14 days (D15), + 21 days (D22), + 28 days (D29), D32 to EOH, and at other days if significant event(s) occur. The patient will be contacted by telephone 3 and 6 months after discharge regarding any SAEs and to confirm survival, as feasible (Table 3).

### 4.16.5. Review of Adverse Events

The Investigator or qualified designee will assess each patient to evaluate for potential new or worsening AEs as specified in Table 3. AEs will be graded and recorded according to National Cancer Institute Common Terminology Criteria for Adverse Events (NCI-CTCAE) version 5.0. Toxicities will be characterized in terms that include seriousness, causality, toxicity grading, and action taken with regards to study drug (see Section 8.1.1)

Any AE/SAE while on study will be reported as outlined in Section 8.1.5. Certain events should also be reported to the Sponsor as adverse events of special interest (AESIs); the definition of AESI is provided in Section 8.1.3.

#### 4.16.5.1. Physical Examination, Vital Signs, Electrocardiograms, and Imaging

Please refer to Table 3.

### 4.16.6. Efficacy Assessments

Efficacy will be assessed with the following parameters:

- Intubation/continuation/discontinuation of intubation/mechanical ventilation
- Supportive oxygenation interventions
- Baseline and every 12 hours as feasible: C-reactive protein, D-dimers, ferritin, IL-1, IL-6 to assess CRS evolution
- Mortality

- Decrease in viral burden from upper respiratory tract samples collected sequentially under treatment
- Persistence of allogeneic iNKT cells
- Baseline and every 24 hours as feasible: complete blood count with differential, comprehensive metabolic panel, procalcitonin, B-type natriuretic peptide, fibrinogen, prothrombin time, partial thromboplastin time, magnesium, angiotensin II level
- Available image (X-ray, CT-scans) to evaluate respiratory involvement (ARDS)
- Change in lung fitness through assessment of respiratory function
- Time from dosing to viral clearance
- Prevention of secondary infections

### 4.16.7. Pharmacokinetics, Immunogenicity, and Pharmacogenomics Assessments

Peripheral blood mononuclear cell samples for agenT-797 persistence and immunogenicity assessments will be collected for all patients at the timepoints described in Table 3. The Sponsor may conduct research on DNA (blood and bronchial) specimens collected during this trial (pharmacogenomics).

#### 4.16.7.1. Laboratory Tests

In addition to standard laboratory testing per hospital guidelines, other study-specific tests are provided in Table 3. Full human leukocyte antigen-typing (blood) will also be performed.

# 5. TREATMENT OF PATIENTS

## 5.1. Description of Study Drug

### 5.1.1. Manufacturing of agenT-797

agenT-797 is a cell therapy consisting of ≥ 95% allogeneic human unmodified iNKT cells isolated from 1 healthy donor mononuclear cell apheresis unit and expanded ex vivo at a contractual development and manufacturing organization according to current Good Manufacturing Practice (GMP). At the end of the manufacturing process, cells are immediately formulated using CryoStor CS10 containing albumin (human) 25% before aseptic filling into freezing bags. The drug product is stored frozen in the vapor phase of liquid nitrogen (≤ -140°C).

### 5.1.2. Infusion of agenT-797

After thawing and dilution (see Section 6.4.1), agenT-797 will be administered as a single intravenous (IV) infusion of allogeneic iNKT cells over 10 to 30 minutes. Patients will be monitored in the clinic for at least 4 hours postinfusion.

In the event of reactions/toxicities during the infusion, the infusion rate may be modified, but the total infusion time should not exceed 60 minutes.

## 5.2. Supportive Care

Supportive care will be per the Investigator and institutional guidelines. Patients will continue standard of care treatment, as required.

## 5.3. Prior and Concomitant Medication and Therapy

Any medication that is considered necessary for the patient's welfare and will not interfere with agenT-797 (see below), may be given at the discretion of the treating Investigator. All concomitant therapies must be reported in the case report form (CRF) throughout the study.

### 5.3.1. Premedication with agenT-797 Infusion

Thirty minutes prior to the commencement of the agenT-797 infusion, patients will be given paracetamol (500 to 1000 mg orally) and diphenhydramine (50 mg orally), or IV equivalent, as premedication and/or as per local institutional guidelines.

### 5.3.2. Recording of Concomitant Medication

Concomitant medications for medically significant AEs should be recorded until the AE has resolved or is considered stable (Section 8.1.5).

### 5.3.3. Prohibited Medications

Prohibited medications include the following:

- Any experimental therapy that has not been registered for other indication(s) at the time of agenT-797 dosing
- QT prolonging drugs should be avoided or switched to non-QT altering alternatives, whenever possible

## 5.4. Risks Associated with agenT-797 Administration

### 5.4.1. Possible Adverse Events Following Treatment with agenT-797 Infusion

The most common anticipated potential toxicities resulting from treatment with agenT-797 are outlined below. Some of these possible AEs have also been further defined as AESIs in Section 8.1.3.

#### 5.4.1.1. Cytokine Release Syndrome

CRS is a systemic inflammatory response characterized by a spectrum of severity, ranging from flu-like symptoms (i.e., fever, myalgia, nausea/vomiting), to hypotension, respiratory and renal failure, and uncontrolled coagulopathy. The onset of CRS can typically occur between 24 hours

and 14 days after the completion of T cell administration. Therefore, after agenT-797 infusion, should CRS occur, it should be managed by the Investigator per institutional guidelines.

The American Society for Transplantation and Cellular Therapy (ASTCT) have published consensus guidelines for grading of CRS (Lee 2019). The ASTCT consensus grading guidelines are presented in Table 4 and align with management algorithms developed by Lee and colleagues (Riegler 2019), which are to be used as a guide for the Investigator in the assessment of CRS.

**Table 4: ASTCT CRS Consensus Grading**

| Parameter | Grade 1 | Grade 2 | Grade 3 | Grade 4 |
|---|---|---|---|---|
| **Fever*** | Temperature ≥ 38°C | Temperature ≥ 38°C | Temperature ≥ 38°C | Temperature ≥ 38°C |
| | | **With** | | |
| **Hypotension** | None | Not requiring vasopressors | Requiring a vasopressor with or without vasopressin | Requiring multiple vasopressors (excluding vasopressin) |
| | | **And/or**† | | |
| **Hypoxia** | None | Requiring low-flow nasal cannula‡ or blow-by | Requiring high-flow nasal cannula,‡ facemask, nonrebreather mask, or Venturi mask | Requiring positive pressure (e.g., CPAP, BiPAP, intubation and mechanical ventilation) |

From Lee 2019.

Abbreviations: ASTCT: American Society for Transplantation and Cellular Therapy; BiPAP: bilevel positive airway pressure; CPAP: continuous positive airway pressure; CRS: cytokine release syndrome; NCI-CTCAE: National Cancer Institute Common Terminology Criteria for Adverse Events.

Organ toxicities associated with CRS may be graded according to NCI-CTCAE v5.0 but they do not influence CRS grading.

* Fever is defined as temperature ≥ 38°C not attributable to any other cause. In patients who have CRS then receive antipyretic or anti-cytokine therapy such as tocilizumab or steroids, fever is no longer required to grade subsequent CRS severity. In this case, CRS grading is driven by hypotension and/or hypoxia.

† CRS grade is determined by the more severe event: hypotension or hypoxia not attributable to any other cause. For example, a patient with temperature of 39.5°C, hypotension requiring 1 vasopressor, and hypoxia requiring low-flow nasal cannula is classified as Grade 3 CRS.

‡ Low-flow nasal cannula is defined as oxygen delivered at ≤ 6 L/min. Low flow also includes blow-by oxygen delivery, sometimes used in pediatrics. High-flow nasal cannula is defined as oxygen delivered at > 6 L/min.

Further, ensure at least 3 vials of tocilizumab, or other anti–IL-6 agents, or current standard treatment regimens are available:

- Administration of tocilizumab 8 mg/kg IV over 1 hour (not to exceed 800 mg) or other anti–IL-6 agents or current standard of care treatment. Repeat tocilizumab or other anti-IL-6 agents or current standard of care treatment every 8 hours as needed if not responsive to IV fluids or increasing supplemental oxygen. Limit to a maximum of 3 doses in a 24-hour period with a maximum total of 4 doses.

### 5.5. Treatment Compliance

Patients who have enrolled in the study are hospitalized (Section 4.7.1). Patients who are administered agenT-797 will have treatment recorded in the CRF and Investigators will follow all guidance for study drug management (Section 6).

### 5.6. Randomization and Blinding

This is an open-label study without randomization or blinding of data.

## 6. STUDY DRUG MATERIALS AND MANAGEMENT

### 6.1. Study Drug

The investigational medicinal product, agenT-797, is provided formulated with albumin (human) 25% and CryoStor CS10 before aseptic filling into Saint Gobin freezing bags of 2 sizes (20F containing 15 mL, and 62F containing 50 mL) at a concentration of $2.5 \times 10^7$ cells/mL (Section 5.1.1).

### 6.2. Study Drug Packaging and Labeling

Packaging, labeling and release of the finished investigational medicinal product will be in accordance with applicable local regulatory requirements and applicable GMP guidelines.

All study drugs must be kept in a secure place under appropriate storage conditions. The Sponsor or its representatives must be granted access on reasonable request to check drug storage, dispensing procedures, and accountability records. Please see Pharmacy Manual for additional details.

### 6.3. Study Drug Storage

Bulk agenT-797 cells will be cryopreserved and stored in the vapor phase of a liquid nitrogen tank (≤ -140°C). agenT-797 cells for administration will be evaluated against preset release criteria. agenT-797 cells must remain frozen until the patient is ready for treatment to ensure viable allogeneic iNKT cells are administered to the patient. agenT-797 bags must be protected from light.

### 6.4. Study Drug Preparation

#### 6.4.1. Thawing of the Infusion Bag and Dilution

Do not thaw the investigational product until it is ready to be used. Once agenT-797 has been thawed, it will be diluted with Plasma-Lyte A in a 1 to 1 ratio. Once thawed and diluted, it should be infused within 30 to 60 minutes. Details on thawing of agenT-797 are provided in the Pharmacy Manual.

## 6.5. ADMINISTRATION

Details are provided in the Pharmacy Manual.

## 6.6. STUDY DRUG ACCOUNTABILITY AND DISPOSAL

The Investigator is responsible to maintain accurate records of study drug shipment receipts from the manufacturer including patient dispensing, product destruction, and documentation of adequate storage of study drug while at the site. Local biosafety guidelines should be followed for unused medicinal product or waste material disposal. Refer to the Pharmacy Manual.

# 7. STUDY ASSESSMENTS

Please see the Schedule of Assessments provided in Table 3 for details and the timing of each clinical assessment and/or procedure.

# 8. ADVERSE AND SERIOUS ADVERSE EVENTS

## 8.1. DEFINITIONS

### 8.1.1. Adverse Event

An AE is defined in the International Council for Harmonisation (ICH) Good Clinical Practice (GCP) guideline as “any untoward medical occurrence in a patient or clinical investigation patient administered a pharmacological product and that does not necessarily have a causal relationship with this treatment.”

This includes:

- An undesirable medical condition, which can be symptoms, signs, or the abnormal results of a laboratory investigation.
- An event that is part of infection with SARS-CoV-2 or influenza and is captured in the study as a clinical activity measure, which does not need to be recorded as an AE.
- Any deterioration of the disease under study and associated symptoms or findings, which should not be regarded as an AE as far as the deterioration can be anticipated.

### 8.1.2. Serious Adverse Event

The term AE is used generally to include any AE whether serious or non-serious.

An SAE is an AE that fulfills one or more of the following criteria:

- It results in death
- It is immediately life-threatening
- It prolongs existing hospitalization

- It results in persistent or significant disability or incapacity, or substantial disruption of the ability to conduct normal life function
- It is or results in a congenital abnormality or birth defect
- It is an important medical event that may jeopardize the patient or may require medical intervention to prevent one of the outcomes listed above

Re-admission after the patient has been discharged for reasons other than administrative, will be considered SAEs.

All SAEs that occur after any from the time of the ICF signature through 30 days postinfusion, and 6 months in the Safety Follow-up period, after following the cessation of treatment, whether or not they are related to the study, must be recorded on forms provided by the Sponsor.

### 8.1.3. AESIs

AESIs are defined as the following events:

- CRS Grade ≥ 2 (ASTCT consensus; Lee 2019). See Section 5.4.1.1
- Hypersensitivity or infusion-related reactions Grade ≥ 2 (as per NCI-CTCAE v5.0) during and up to 24 hours following administration of agenT-797
- Acute GVHD, any stage, as per national/local/institutional guidelines
- Immune effector cell-associated neurotoxicity syndrome Grade ≥ 2 (ASTCT consensus; Lee 2019)

The AESI should be entered on the AE CRF and reported using the procedures detailed in Recording and Reporting of AEs in Section 8.1.5. Even if the AESI does not meet the SAE criteria, it must still be reported using the SAE form and in an expedited manner but should be noted as non-serious on the SAE form and the AE CRF.

AESIs both non-serious and serious identified in this protocol will be followed from the onset through resolution/outcome, regardless of day of study when identified.

### 8.1.4. Dose-Limiting Toxicity Criteria

The DLT observation period is defined as 14 days after infusion of agenT-797. The nature, frequency, time to onset, severity of toxicities, medical history, and concomitant medication, as well as the success of standard medical management, will be analyzed to determine if a given toxicity should be considered a DLT by the SMC per charter.

A DLT is defined as any of the following at least possibly related AEs graded using NCI-CTCAE v5.0:

- General:
  - Any Grade 4 or 5 toxicity
- Hematologic:
  - Any Grade 3 hematologic toxicity that lasts for more than 7 days despite treatment

- Non-hematologic:
  - Any Grade 3 non-hematologic toxicity that lasts for more than 72 hours despite adequate treatment
  - Exceptions: Grade 3 events with a different definition of duration that are considered DLTs are the following:
    - Grade 3 renal or hepatic toxicity that lasts 7 days or longer
    - Any Grade 3 or higher toxicity that is cardiac, respiratory, or neurologic of any duration
- Immune:
  - Any Grade 2 acute GVHD that requires steroid therapy for > 7 days or that has progression of clinical signs and symptoms after 3 days of steroids or that has partial response after 14 days of treatment, or GVHD Grade 3 or higher
  - Any **new** Grade 3 CRS lasting more than 72 hours and/or **new** Grade 4 CRS of any duration at least possibly related to agenT-797. For patients presenting with Grade 2 or 3 CRS at baseline, worsening of baseline CRS grade, judged as at least possibly related to agenT-797, and occurring within 4 hours of infusion (Lee 2019)
  - Any clinically significant Grade 3 autoimmune disorder that lasts for more than 72 hours at Grade 3 despite adequate treatment or any Grade 4 autoimmune disorder of any duration

### 8.1.5. Recording and Reporting of Adverse Events

AE reporting and management will be performed according to GCP and relevant guidelines.

### 8.1.6. Recording of Adverse Events/Serious Adverse Events

The Principal Investigator is responsible for ensuring that all AEs are recorded on the CRF during the study. All SAEs must be reported on the AE form in the CRF. All AEs, non-serious and serious, related or unrelated, need to be recorded from the time of ICF signature through 30 days, and 6 months in the Safety Follow-up period, after infusion, and followed up until resolution, if feasible (Section 4.10).

AEs will be categorized as SAEs, AESIs, or DLTs, as applicable, and reported based on applicable guidelines (Section 8).

### 8.1.7. Relationship

The Investigator's assessment of the relationship of an AE to study medication is part of the documentation process. If there is any doubt as to whether a clinical observation is an AE, the event should be reported. The relationship or association of the study medication in causing or contributing to the AE will be characterized by the Investigator using the following classification and criteria:

- Possibly or probably related: A clinical event, including laboratory test abnormality, with a reasonable time sequence to study medication administration that might or might not be also explained by concurrent disease or other drugs or chemicals.
- Not related: A clinical event, including laboratory test abnormality, judged to be clearly and incontrovertibly due to extraneous causes (e.g., diseases, environment), or with a temporal relationship to study medication administration that makes a causal relationship.

The Investigator may determine that an AE requires removal from the study. A patient may also voluntarily withdraw from treatment due to an AE, or for any other reason. The AE from either eventuality should be followed until resolution or to the point where feasible.

When assessing the relationship of a DLT to study medication, Investigators must choose related if they think the event is at least possibly related to the study medication, for the purposes of expedited regulatory reporting (Section 8.1.4). Investigators will be asked to assess the relationship of all AEs to the investigational agent and to the underlying disease (SARS-CoV-2 infection or influenza).

### 8.1.8. Severity

Severity or intensity will be assessed according to the NCI-CTCAE v5.0 severity grading scale:

- Grade 1: Mild (awareness of sign or symptom, but easily tolerated)
- Grade 2: Moderate (discomfort sufficient to cause interference with normal activities)
- Grade 3: Severe (incapacitating, with inability to perform normal activities)
- Grade 4: Life-threatening (urgent intervention indicated)
- Grade 5: Death related to AE

### 8.1.9. Pregnancy

Should a pregnancy occur, it must be reported and recorded on the Sponsor's pregnancy form.

Pregnancy in itself is not regarded as an AE unless there is a suspicion that a study drug may have interfered with the effectiveness of a contraceptive medication. If a pregnancy occurs in a female patient 90 days after receiving the study drug, then Investigators will inform appropriate Sponsor representatives immediately, but no later than 24 hours of when she/he becomes aware of it.

The outcome of all pregnancies (spontaneous miscarriage, elective termination, normal birth, or congenital abnormality) must be followed up to birth, and where possible, 6 to 8 weeks after birth and documented even if the patient was discontinued from the study.

All reports of congenital abnormalities/birth defects are SAEs. Spontaneous miscarriages should also be reported and handled as SAEs. Elective abortions without complications should not be handled as AEs.

Pregnancy of a patient's partner is not considered to be an AE. However, any conception occurring from the date of dosing until 120 days after dosing should be reported to the Sponsor

and followed up for its outcome. The Investigators will inform the appropriate Sponsor representative immediately, but no later than 24 hours of when he or she becomes aware of it.

### 8.1.10. Reporting of Serious Adverse Events

All SAEs must be reported from the time of ICF signature through 6 months in the Safety Follow-up period after infusion with agenT-797, and followed-up until resolution, if feasible.

All SAEs must be reported to the Sponsor within 24 hours of the first awareness of the event.

Any death, SAE, or pregnancy (including pregnancy of a partner) experienced by a patient regardless of relationship to study drug, or any death that occurs within 6 months after receiving study drug, must be promptly reported (within 24 hours of the Investigator becoming aware of the event) in the CRF and/or via email.

AESIs, even if they do not meet the serious criteria, must be reported using the SAE form and in an expedited manner (within 24 hours of the Investigator becoming aware of the event), but should be noted as non-serious on the SAE form and the AE CRF (Section 8.1.3).

The Investigator must complete, sign and date the SAE pages, verify the accuracy of the information recorded on the SAE pages with the corresponding source documents.

For overdoses associated with a SAE, standard reporting timelines apply (Section 8.1.6).

**Reporting Serious Adverse Events**

By electronic media: Adverse.Events@Agenusbio.com

or

Paper (Fax: +1-781-674-4261)

Additional follow-up information, if required or available, should be completed within 1 business day of receipt and this should be completed on a follow-up SAE form and placed with the original SAE information and kept with the appropriate section of the CRF and/or study file. The Sponsor is responsible for notifying the relevant regulatory authorities of certain events. It is the Principal Investigator's responsibility to notify the IRB/IEC of all SAEs. Investigators will also be notified of all unexpected, serious, drug-related events (7/15 Day Safety Reports) that occur during the clinical trial. Each site is responsible for notifying its IRB/IEC of these additional SAEs.

## 8.2. SAFETY MONITORING COMMITTEE

The SMC is tasked to review the available safety and other clinical data from patients enrolled into the study. The decisions and decision-making of the SMC will be documented and provided to the Investigators. Details regarding the SMC will be provided in a separate charter.

# 9. STATISTICS

A statistical analysis plan (SAP) is being prepared as a separate document and will include a more technical and detailed description (including templates for tables, listings, and figures) of the planned statistical summaries. The SAP will be finalized before initiating any statistical analyses.

Unless otherwise stated, tabulation of summary statistics and data analysis will be performed using SAS Version 9.4 or later or R Version 6.0 or later.

This study is exploratory in nature; no formal statistical comparisons are planned.

## 9.1. Safety Analyses

All safety analyses will be performed on the Safety Analysis Set. Interim safety data will be examined on an ongoing basis to ensure patient safety and to comply with the clinical study Dose Escalation rules.

## 9.2. Sample Size

The total sample size is expected to be up to approximately 43 evaluable treated patients. Since the study uses 3+3 design with 3 dose levels, up to 18 patients may be needed in Dose Escalation part (Part 1). Additional patients will be enrolled to the Expansion Cohort. Please refer to the SAP for details. A matched control may be used to complete the comparative analysis. This will be defined in the SAP.

## 9.3. Criteria for the Termination of the Study

No statistical stopping rules will be formulated for this study.

## 9.4. Procedure for Accounting for Missing, Unused, and Spurious Data

Missing, unused, and spurious data will be dealt with as such. There is no intention to implement any procedure for replacing missing data.

## 9.5. Deviations from the Original Planned Analysis

Any deviations from the original statistical plan as described in this protocol will be agreed upon by the Sponsor, documented, and justified in a protocol amendment, the final SAP, or the clinical study report, as appropriate.

# 10. ETHICAL AND REGULATORY OBLIGATIONS

## 10.1. Informed Consent

The Principal Investigator(s) at each center will ensure that the patient, or the duly appointed health care proxy with authority for consent, is given full and adequate oral and written information about the nature, purpose, possible risk, and benefit of the study, see Section 4.6.1.1.

## 10.2. Institutional Review Board/Independent Ethics Committee

The Principal Investigator must obtain IRB/IEC approval for the investigation. Initial IRB/IEC approval and all materials approved by the IRB/IEC for this study, including the patient consent form and recruitment materials (if applicable), must be maintained by the Investigator and made available for inspection.

The study will be performed in accordance with ethical principles that have their origin in the Declaration of Helsinki and are consistent with ICH GCP, applicable regulatory requirements, and the Sponsor's policy on bioethics.

## 10.3. Study Documentation Requirements

The Investigator is responsible for providing study documentation, such as those items outlined in Section 8 of ICH E6, as required by the Sponsor or designee and in compliance with local and regional regulations.

## 10.4. Patient Confidentiality

The Investigator must ensure that the patient's confidentiality is maintained on all study-related documents and as per local regulations. In compliance with federal regulations and ICH GCP guidelines, it is required that the Investigator and institution permit authorized representatives of the company or the company-appointed external auditor, the regulatory agency(s) or inspectors, and the IRB/IEC direct access to review the patient's original medical records for verification of study-related procedures and data.

# 11. ADMINISTRATIVE AND LEGAL OBLIGATIONS

## 11.1. Protocol Amendments and Study Termination

Protocol amendments, except where necessary to eliminate an immediate hazard to patients, must be made only with the prior approval of the Sponsor. Agreement from the Investigator must be obtained for all protocol amendments and amendments to the ICF. The IRB/IEC must be informed of all amendments and give approval. The Investigator must send a copy of the approval letter from the IRB/IEC to the Sponsor, or the appointed contract research organization. Both the Sponsor and the Investigator reserve the right to terminate the study according to the study contract. The Investigator should notify the IRB/IEC in writing of the study's completion

or early termination and send a copy of the notification to The Sponsor, or the appointed contract research organization.

Although the Sponsor has every intention of completing the study, it reserves the right to discontinue the study at any time for clinical or administrative reasons (see Section 4.5.4).

## 11.2. Data Handling and Recordkeeping

## 11.3. Data Collection

The CRF is the primary source for collection of all patient data. Data will be collected per GCP ICH E6(R2) and all applicable regulatory requirements.

### 11.3.1. Inspection of Records

The Sponsor or its representative will be allowed to conduct site visits to the investigation facilities for the purpose of monitoring any aspect of the study.

### 11.3.2. Retention of Records

The Principal Investigator must maintain all documentation relating to the study for a period of 2 years following the discontinuance of the test article for investigation. If it becomes necessary for the Sponsor or its representative, or the Regulatory Authority to review any documentation relating to the study, the Investigator must permit access to such records.

### 11.3.3. Data Quality Assurance

To ensure compliance with GCP ICH E6(R2) and all applicable regulatory requirements, the Sponsor or its representatives may conduct a quality assurance audit. Please see Section 11.3.6 for more details regarding the audit process.

### 11.3.4. Study Monitoring

During the study, a monitor from the Sponsor or its representative will ensure compliance with GCP ICH E6(R2) and all applicable regulatory requirements.

### 11.3.5. Protocol Deviations

The Investigator or designee must document and explain in the patient's source documentation any deviation from the approved protocol. The Investigator may implement a deviation from the protocol to eliminate an immediate hazard to study patients without prior IRB/IEC approval. A deviation from the protocol is an unintended or unanticipated departure from the procedures or processes approved by the Sponsor and the IRB/IEC and agreed to by the Investigator. Protocol deviations will be documented, and the IRB/IEC should be notified of all significant deviations in a timely manner and as required by the specific IRB/IEC.

### 11.3.6. Audits and Inspections

As per ICH GCP and the Sponsor's audit plans, representatives of the Sponsor (or designees), a regulatory authority, or an IRB/IEC may visit the site to perform audits or inspections, including source data verification.

## 11.4. PUBLICATION POLICY

The Sponsor recognizes and supports the publication and dissemination of scientific information as a means of furthering knowledge. The general strategy regarding publication of the study will be mutually agreed upon by the Investigator and Sponsor. However, to protect its commercial interests, the Sponsor reserves the right to manage the publication of all study results. The Investigator agrees that oral and written communication to third parties of any procedures or results from the study is subject to prior written consent of the Sponsor. Data are the property of the Sponsor and cannot be published without prior authorization from the Sponsor, but data and publication thereof will not be unduly withheld.

# 12. LIST OF REFERENCES

ARDS Definition Task Force. Acute Respiratory Distress Syndrome: The Berlin Definition. *JAMA*. 2012;307(23):2526-2533.

Beigel JH, Tomashek KM, Dodd LE, et al. Remdesivir for the Treatment of Covid-19 – Preliminary Report. *N Engl J Med*. 2020;383(10):992-993.

Boktor SW, Hafner JW. Influenza. In: StatPearls. Treasure Island (FL): StatPearls Publishing; July 18, 2021.

Bowles SK, Lee W, Simor AE, et al. Use of oseltamivir during influenza outbreaks in Ontario nursing homes, 1999-2000. *J Am Geriatr Soc*. 2002;50(4):608-616.

Cain DW, Cidlowski JA. After 62 years of regulating immunity, dexamethasone meets COVID-19. *Nat Rev Immunol*. 2020;20(10):587-588.

Chowell G, Bertozzi SM, Colchero MA, et al. Severe respiratory disease concurrent with the circulation of H1N1 influenza. *N Engl J Med*. 2009;361(7):674-679.

Exley MA, Friedlander P, Alatrakchi N, et al. Adoptive Transfer of Invariant NKT Cells as Immunotherapy for Advanced Melanoma: A Phase I Clinical Trial. *Clin Cancer Res*. 2017;23(14):3510-3519.

Field JJ, Majerus E, Ataga KI, et al. NKTT120, an anti-iNKT cell monoclonal antibody, produces rapid and sustained iNKT cell depletion in adults with sickle cell disease. *PloS One*. 2017;12(2):e0171067.

Fuchs EJ. Haploidentical transplantation for hematologic malignancies: where do we stand? *Hematology Am Soc Hematol Educ Program*. 2012;2012:230-6.

Hu CK, Venet F, Heffernan DS, et al. The role of hepatic invariant NKT cells in systemic/local inflammation and mortality during polymicrobial septic shock. *J Immunol.* 2009;182(4):2467-2475.

Huang C, Wang Y, Li X, et al. Clinical features of patients infected with 2019 novel coronavirus in Wuhan, China. *Lancet*. 2020;395:497-506.

Juno JA, Keynan Y, Fowke KR. Invariant NKT Cells: Regulation and Function during Viral Infection. *PloS Pathog.* 2012;8(8):e1002838.

Kok WL, Denney L, Benam K, et al. Invariant NKT cells reduce accumulation of inflammatory monocytes in the lungs and decrease immune-pathology during severe influenza A virus infection. *J Leukoc Biol*. 2012;91(3):357-68.

Kunii N, Horiguchi S, Motohashi S, et al. Combination therapy of in vitro-expanded natural killer T cells and alpha-galactosylceramide-pulsed antigen-presenting cells in patients with recurrent head and neck carcinoma. *Cancer Sci*. 2009 Jun;100(6):1092-8.

Laport GG, Sheehan K, Baker J, et al. Adoptive immunotherapy with cytokine induced killer cells for patients with relapsed hematologic malignancies after allogeneic hematopoietic cell transplantation. *Biol Blood Marrow Transplant*. 2011;17(11):1679-87.

Lee DW, Santomasso BD, Locke FL, et al. ASTCT Consensus Grading for Cytokine Release Syndrome and Neurologic Toxicity Associated with Immune Effector Cells. *Biol Blood Marrow Transplant.* 2019;25(4):625-638.

Lee S, Lim J, Ryu D, et al. Circulating immune cell phenotype can predict the outcome of lenalidomide plus low-dose dexamethasone treatment in patients with refractory/relapsed multiple myeloma. *Cancer Immunol Immunother*. 65:983-994 (2016).

Lehnert R, Pletz M, Reuss A, Schaberg T. Antiviral Medications in Seasonal and Pandemic Influenza. *Dtsch Arztebl Int*. 2016;113(47):799-807.

Liu K, Fang YY, Deng Y, et al. Clinical characteristics of novel coronavirus cases in tertiary hospitals in Hubei Province. *Chin Med J (Engl)*. 2020;(Epub ahead of print).

Mavers M, Maas-Bauer K, Negrin RS. Invariant natural killer T cells as suppressors of graft-versus-host disease in allogeneic hematopoietic stem cell transplantation. *Front. Immunol*. 2017;8:900.

McGeer A, Green KA, Plevneshi A, et al. Antiviral therapy and outcomes of influenza requiring hospitalization in Ontario, Canada. *Clin Infect Dis*. 2007;45(12):1568-1575.

Miller JS, Soignier Y, Panoskaltsis-Mortari A, et al. Successful adoptive transfer and in vivo expansion of human haploidentical NK cells in patients with cancer. *Blood*. 2005;105(8):3051-7.

Motohashi S, Ishikawa A, Ishikawa E, et al. A phase I study of in vitro expanded natural killer T cells in patients with advanced and recurrent non-small cell lung cancer. *Clin Cancer Res*. 2006 Oct 15;12:6079-86.

Muthuri SG, Venkatesan S, Myles PR, et al. Effectiveness of neuraminidase inhibitors in reducing mortality in patients admitted to hospital with influenza A H1N1pdm09 virus infection: a meta-analysis of individual participant data. *Lancet Respir Med*. 2014;2(5):395-404.

RECOVERY Collaborative Group. Dexamethasone in Hospitalized Patients with Covid-19 – Preliminary Report. *NEJM*. 2020.

Riegler LL, Jones GP, Lee DW. Current approached in the grading and management of cytokine release syndrome after chimeric antigen receptor T-cell therapy. *Ther Clin Risk Manag.* 2019;15:323-335

Simonsen L, Clarke MJ, Schonberger LB, Arden NH, Cox NJ, Fukuda K. Pandemic versus epidemic influenza mortality: a pattern of changing age distribution. *J Infect Dis*. 1998;178(1):53-60.

SOFA score accessed on 11 Nov 2021 from https://www.mdcalc.com/sequential-organ-failure-assessment-sofa-score

Taubenberger JK, Morens DM. 1918 Influenza: the mother of all pandemics. *Emerg Infect Dis*. 2006;12(1):15-22.

Topp MS, van Meerten T, Houot R, et al. Earlier Steroid Use with Axicabtagene Ciloleucel (Axi-Cel) in Patients with Relapsed/Refractory Large B Cell Lymphoma. *Blood*. 2019;134(Supplement_1):243.

Wang D, Hu B, Hu C, et al. Clinical characteristics of 138 hospitalized patients with 2019 novel coronavirus-infected pneumonia in Wuhan, China. *JAMA.* 2020;(Epub ahead of print).

Wang Y, Zhang D, Du G, et al. Remdesivir in adults with severe COVID-19: a randomised, double-blind, placebo-controlled, multicentre trial [published correction appears in *Lancet*. 2020 May 30;395(10238):1694]. *Lancet*. 2020;395(10236):1569-1578.

Yamasaki K, Horiguchi S, Kurosaki M, et al. Induction of NKT cell-specific immune responses in cancer tissues after NKT cell-targeted adoptive immunotherapy. *Clin Immunol*. 2011 Mar;138(3):255-65.

# APPENDIX 1. Protocol Approval and Signature Page

| | |
|---|---|
| **Title** | **A Phase 1/2 Study of agenT-797 to Treat Moderate to Severe Acute Respiratory Distress Syndrome Secondary to SARS-COV-2 or Influenza** |
| **Clinical Trial Version / Date** | Version 9.0, 27 Apr 2022 incorporating Amendment 8 |

I approve the design of the clinical trial.

| Signature | Date of Signature |
|---|---|

| | |
|---|---|
| **Name, academic degree** | Waldo Ortuzar Feliu, MD |
| **Function** | Medical Monitor |
| **Institution** | Agenus, Inc. |
| **Address** | 3 Forbes Road, Lexington, MA 02421, USA |
| **Telephone** | +1 781-674-4455 |
| **E-mail address** | Waldo.Ortuzar@Agenusbio.com |

# APPENDIX 2. ACUTE RESPIRATORY DISTRESS SYNDROME, BERLIN DEFINTION

The ARDS was defined in 1994 by the American-European Consensus Conference; since then, issues regarding the reliability and validity of this definition have emerged. Using a consensus process, a panel of experts convened in 2011 (an initiative of the European Society of Intensive Care Medicine endorsed by the American Thoracic Society and the Society of Critical Care Medicine) and developed the Berlin Definition, focusing on feasibility, reliability, validity, and objective evaluation of its performance.

- **A draft definition** proposed 3 mutually exclusive categories of ARDS based on degree of hypoxemia on ventilator settings with PEEP of ≥ 5 cm $H_2O$:
  - Mild: The $PaO_2/FiO_2$ is > 200 mmHg, but ≤ 300 mmHg
  - Moderate: The $PaO_2/FiO_2$ is > 100 mmHg, but ≤ 200 mmHg
  - Severe: The $PaO_2/FiO_2$ is ≤ 100 mmHg

- **4 ancillary variables** for severe ARDS:
  - Radiographic severity (3 or 4 quadrants with opacities)
  - Respiratory system compliance (≤ 40 ml/cm $H_2O$) and/or corrected expired volume per minute (≥ 10 L/min)
  - Positive end-expiratory pressure (≥ 10 cm $H_2O$)

- **The Berlin Definition** of ARDS requires that all of the following criteria be present for diagnosis:
  - Respiratory symptoms must have begun within 1 week of a known clinical insult, or the patient must have new or worsening symptoms during the past week.
  - Bilateral opacities must be present on a chest radiograph or CT scan. These opacities must not be fully explained by pleural effusions, lobar collapse, lung collapse, or pulmonary nodules.
  - The patient's respiratory failure must not be fully explained by cardiac failure or fluid overload. An objective assessment (e.g., echocardiography) to exclude hydrostatic pulmonary edema is required if no risk factors for ARDS are present.
  - A moderate to severe impairment of oxygenation must be present, as defined by the ratio of arterial oxygen tension to fraction of inspired oxygen ($PaO_2/FiO_2$). The severity of the hypoxemia defines the severity of the ARDS.

**Supplementary protocol file 2.** Statistical analysis plan.

# MiNK Therapeutics, Inc.

## A PHASE 1/2 STUDY OF AGENT-797 To TREAT MODERATE TO SEVERE ACUTE RESPIRATORY DISTRESS SYNDROME SECONDARY TO SARS-COV-2 OR INFLUENZA

## Statistical Analysis Plan

Version: 1.0

Based on

Protocol Number: C-1300-01 Version 9.0 dated 27 Apr, 2022

# TABLE OF CONTENTS

## Contents

APPROVAL ……………………………………………………………………………... 2
1 INTRODUCTION ........................................................................................................... 7
2 STUDY OBJECTIVES ................................................................................................... 7
2.1 Primary Objectives ............................................................................................7
2.2 Secondary Objectives ........................................................................................7
2.3 Exploratory Objectives ......................................................................................7
3 INVESTIGATIONAL PLAN............................................................................................ 7
3.1 Overall Study Design and Plan..........................................................................7
3.2 Number of Patients ............................................................................................8
3.3 Treatment Assignment.......................................................................................8
3.4 Staggered Cohort Enrollment ............................................................................8
3.5 Dose Escalation Criteria ....................................................................................9
3.6 Dose-Limiting Observation Period.....................................................................9
3.7 Maximum Tolerated Dose ..................................................................................9
3.8 Safety Criteria for Adjustment or Stopping Doses ............................................9
3.9 Treatment and Evaluation Period ....................................................................10
4. Objectives, Endpoints and Estimators .................................................................... 11
4 STATISTICAL METHODS.......................................................................................... 13
4.1 Data Quality Assurance ...................................................................................13
4.2 General Presentation Considerations..............................................................13
4.3 Software14
4.4 Study Patients .................................................................................................14
4.4.1 Disposition of Patients ..........................................................................14
4.4.2 Protocol Deviations...............................................................................14
4.5 Analysis Sets....................................................................................................14
4.6 Demographics and Baseline Characteristics....................................................15
4.7 Diagnosis and Extent of Disease .....................................................................15
4.8 Medical History and Concomitant Illnesses.....................................................15
4.9 Prior and Concomitant Medications ................................................................15
4.10 Treatment Exposure / Compliance .................................................................16
4.10.1 Treatment Exposure.............................................................................16
4.10.2 Compliance ..........................................................................................16
4.11 Efficacy Evaluation ..........................................................................................16
4.11.1 Analysis and Data Conventions.............................................................16
4.11.2 Efficacy Variables.................................................................................16
4.11.3 Safety Evaluation..................................................................................18
4.11.4 Adverse Events .....................................................................................18
4.11.5 Deaths, Serious Adverse Events, and Other Significant Adverse Events ..................20
4.11.6 Dose-Limiting Toxicity .........................................................................22
4.11.7 Clinical Laboratory Evaluation.............................................................22
4.11.8 Vital Signs, Physical Findings and Other Observations Related to Safety ................23

5 REFERENCE .......... 23
6 Appendix .......... 25

## TABLE OF TABLES

Table 1. Cohorts for agenT-797 .......... 8
Table 2. Objectives, Endpoints, and Estimators .......... 11
Table 3. ASTCT CRS Consensus Grading .......... 21
Table 4. Schedule of Assessments .......... 25

## REVISION HISTORY

| Version No. | Effective Date | Summary of Change(s) |
|---|---|---|
| 1.0 | 7 Jun 2022 | New document based on Protocol v9 incorporating Amendment 8 |

# LIST OF ABBREVIATIONS

| Abbreviation / Acronym | Definition / Expansion |
|---|---|
| AE | Adverse Event |
| AESI | Adverse Event of Special Interest |
| ARDS | Acute Respiratory Distress Syndrome |
| ASTCT | American Society for Transplantation and Cellular Therapy |
| BMI | Body Mass Index |
| CI | Confidence Interval |
| CRS | Cytokine Release Syndrome |
| CS | Clinically Significant |
| CT | Computed Tomography |
| DLT | Dose-Limiting Toxicity |
| DSA | Donor Specific Antibodies |
| ECG | Electrocardiogram |
| eCRF | electronic Case Report Form |
| GVHD | Graft-Versus-Host Disease |
| HLA | Human Leukocyte Antigen |
| ICANS | Immune effector Cell-Associated Neurotoxicity Syndrome |
| ICH | International Conference on Harmonisation |
| ICI | Immune Checkpoint Inhibitor |
| IMP | Investigational Medicinal Product |
| iNKT | invariant Natural Killer T (cells) |
| LIS | Lung Injury Score |
| MedDRA | Medical Dictionary for Regulatory Activities |
| MTD | Maximum Tolerated Dose |
| NCI CTCAE | National Cancer Institute Common Terminology Criteria for Adverse Events |
| NCS | Not Clinically Significant |
| PEEP | Positive End-Expiratory Pressure |
| PRA | Panel Reactive Antibodies |
| PT | Preferred Term |
| RP2D | Recommended Phase 2 Dose |

| Abbreviation / Acronym | Definition / Expansion |
|---|---|
| SAE | Serious Adverse Event |
| SAP | Statistical Analysis Plan |
| SARS-CoV-2 | Severe Acute Respiratory Syndrome-CoronaVirus 2 |
| SD | Standard Deviation |
| SOC | System Organ Class |
| SOFA Score | Sequential Organs Failure Assessment Score |
| SMC | Safety Monitoring Committee |
| TEAE | Treatment-Emergent Adverse Event |
| WHO-DD | WHO Drug Dictionary |

# 1 INTRODUCTION

This statistical analysis plan (SAP) provides a detailed methodology for the summary and statistical analyses based on the study protocol C-1300-01 (version 8, amendment 7 dated 28 February 2022). Any major modifications to the endpoint definitions or analysis will jointly be reflected in a protocol amendment and a new version of the SAP. This SAP adheres to the principles specified in the E9 guideline "Statistical Principles for Clinical Trials" of the International Conference on Harmonisation (ICH).

# 2 STUDY OBJECTIVES

## 2.1 Primary Objectives

- To assess safety and tolerability of agenT-797 infusion

## 2.2 Secondary Objectives

- To assess evidence of improvement and resolution of Acute Respiratory Distress Syndrome (ARDS) following infusion with agent-979
- To assess avoidance of Multi Organ Dysfunction Syndrome.
- To assess evolution of cytokine release syndrome (CRS)
- To assess decay in quantitative (as feasible, or positive/negative) viral burden from upper and lower respiratory, as appropriate
- To assess prevention of viral reactivation

## 2.3 Exploratory Objectives

- To identify biomarkers that could predict clinical activity against severe acute respiratory syndrome-coronavirus 2 (SARS-CoV-2)
- To evaluate persistence and longevity of allogeneic invariant natural killer T-cells (iNKTs) agent-797 with respect to tissue localization in target organs
- To evaluate development of alloimmunity against administered donor cells
- To evaluate whether iNKT cells prevent secondary infections
- To evaluate whether iNKT cells improve lung fitness through assessments of respiratory function.
- To explore whether iNKTs promote viral resolution without lung fibrosis in SARS-CoV-2 (COVID-19) patients.

# 3 INVESTIGATIONAL PLAN

## 3.1 Overall Study Design and Plan

This is a Phase 1/2 study to evaluate the safety and potential efficacy of agenT-797, an unmodified, allogeneic iNKT cell therapy, in patients with mild to severe ARDS secondary to SARS-CoV-2 or influenza, either with intubation or at high risk to be intubated, as determined by the Berlin definition (ARDS 2012).

**Completed:** Part 1 will employ a standard 3+3 dose escalation design of agenT-797. All patients will receive a single infusion of agenT-797. Patients will also receive other treatments and supportive care per discretion of the Principal Investigator. Once the

maximum tolerated dose (MTD) of agenT-797 has been cleared in Part 1, an Expansion Cohort will be opened. A Safety Monitoring Committee (SMC) will be established to assess safety and decide on escalation to next cohort and expansion dose, as well as any protocol modification to include less severe cases.

**Part 2 (Expansion) (Amendment 7)** of the study will evaluate an additional cohorts of up to 15 patients:

- Cohort 4 – patients with moderate to severe ARDS secondary to SARS-CoV-2 or influenza, according to Berlin definition, who are on mechanical intubation

The SMC will continue to review safety.

The number of patients in each cohort is presented in Table 1.

**Table 1. Cohorts for agenT-797**

| Cohort | Number of Patients | Escalation Type | agenT-797 level |
|---|---|---|---|
| 1 | 3-6 | 3+3 | $100 \times 10^6$ cells |
| 2 | 3-6 | 3+3 | $300 \times 10^6$ cells |
| 3 | 6+ | 3+3 | $1000 \times 10^6$ cells |
| 4 | Up to 15 | 3+12 | $1000 \times 10^6$ cells |

### 3.2 Number of Patients

It is estimated that up to approximately 43 patients, requiring or at risk of needing mechanical ventilation with mild to severe ARDS, per the Berlin definition (ARDS 2012), will be enrolled into the study. Additional patients may be added at the discretion of the SMC based on the review of the totality of the data including safety and efficacy data.

No formal sample size calculations were performed since all analyses will be descriptive and exploratory of nature, without any formal testing of hypotheses.

### 3.3 Treatment Assignment

This is an open-label, single-arm study. Patients will receive a single dose of agenT-797. Each patient will stay on the dose level and schedule assigned at trial entry. No randomization and blinding procedures will be applied to treatment assignments.

### 3.4 Staggered Cohort Enrollment

Each cohort will have a minimum of 3 patients enrolled. agenT-797 (Cohort 1) will be administered in the first patient, and treatment will be administered to the next patient 7 days after the last patient was administered agenT-797. Cohort 2 will be administered 14 days after the last patient was dosed in Cohort 1 and will follow the staggered dosing in Cohort 1 (every 7 days/patient). If patients tolerate low dose (Cohort 1) and intermediate dose (Cohort 2) treatment with agenT-797, Cohort 3 may be enrolled after 14 days.

An additional cohort in up to 15 patients will be evaluated:

- Cohort 4 – patients with moderate to severe ARDS secondary to SARS-CoV-2 or influenza, according to Berlin definition, who are on mechanical intubation

## 3.5 Dose Escalation Criteria

A SMC will assess safety, decide on appropriateness of dose escalation, and define the expansion cohort dose.

## 3.6 Dose-Limiting Observation Period

A minimum of 3 patients will be enrolled in each cohort, and the DLT observation period will be 14 days for each patient. Patients will continue to be monitored post–DLT period throughout the study. The totality of the safety data will be factored in by the SMC in the decision-making to move to enrollment of Cohort 2.

If a DLT occurs in 1 of the 3 patients during the DLT period, the cohort will be expanded to a total of 6 patients; if a DLT occurs in ≥ 2 patients in the total cohort of 6 patients, the MTD will be deemed to be exceeded, and that dose will not be escalated. The prior dose level will be evaluated to then proceed into the Expansion Cohort. The interval between patients in each cohort is at least 7 days with a 14-day interval between each cohort (Section 1.3).

The dose of agenT-797 will be escalated if none of the first 3 evaluable patients enrolled has a DLT based on SMC evaluations of data. If a cohort was expanded to include a total of 6 evaluable patients and no DLT occurs in the additional 3 patients, then the dose will be escalated based on the SMC evaluation of data.

After Amendment 7, the SMC will monitor safety on an ongoing basis.

## 3.7 Maximum Tolerated Dose

If a DLT occurs in ≥ 2 patients in a cohort, the MTD will be deemed to be exceeded, and the prior dose level will be evaluated to determine the MTD by increasing enrollment to 6 patients. If the prior dose level was already deemed to be safe and enrolled 6 patients, then it will be defined as the MTD. Dose Escalation will continue until the MTD is reached or the maximum planned dose level is shown to be safe. If none of the first 3 evaluable patients enrolled at the maximum planned dose experience a DLT, an additional 3 patients may be enrolled for a total of 6 patients at the maximum planned dose. Additional patients may also be enrolled in other lower dose cohorts.

## 3.8 Safety Criteria for Adjustment or Stopping Doses

Enrollment and treatment will be temporarily stopped if any of the following occur:

- DLT(s) defined as follows: two Grade 4 DLTs or single Grade 4 neurological toxicity or single Grade 3 or higher Graft-versus-Host Disease (GVHD) as DLT
- Any toxicity that is unexpected, significant, and unacceptable (based on SMC review and discussion), e.g., any occurrence of GVHD Grade 3 or higher

- Any death that occurs within 30 days of agenT-797 administration, except cases reported as related to the primary disease (COVID-19 or influenza)

If any of the above stopping criteria are met, an SMC meeting will occur to review the information and to recommend how to proceed.

The SMC will review any cohort under evaluation as well as the safety of all cohorts. All toxicities, including those outside the 14-day DLT period, will be evaluated.

## 3.9 Treatment and Evaluation Period

Eligible patients will be treated with agenT-797 on Day 1. Assessments will be obtained through Day 32 or until discharge from hospital as outlined in the Schedule of Assessments in Table 3 of the study protocol.

After the patient has received a single infusion of agenT-797, the EOH is defined as patient discharge from the hospital or death.

The Safety Follow-up period will be 30 days, and the patient will be contacted by telephone 3 and 6 months after discharge regarding any SAEs and to confirm survival, as feasible. The first Safety Follow-up Visit will be at Day 30 (± 2), or at EOH. If a patient is discharged at Day 30, 30-day Follow-up will be the same as EOH.

Patients are at any time free to withdraw from the study. Such patients will always be asked about the reason(s) and the presence of any AEs. The Investigator may also elect to discontinue patients from the study, for example, to pursue a different treatment option. Reason(s) for discontinuation will be documented.

Patients who are withdrawn from the study but have completed treatment (infusion of agenT-797), are to be considered evaluable for DLT, and will not be replaced. Any patient who is withdrawn and is not considered evaluable for safety during Dose Escalation will be replaced to ensure a minimum number of evaluable patients.

# 4. OBJECTIVES, ENDPOINTS AND ESTIMATORS

The objectives, endpoints and the corresponding estimators are described in the following table:

**Table 2. Objectives, Endpoints, and Estimators**

| Primary Objectives | Primary Endpoints | Estimators |
|---|---|---|
| • To assess safety and tolerability of agenT-797 infusion | • Number and severity of adverse events (AEs) and dose-limiting toxicities (DLTs) at evaluated dose levels | • Frequency of AEs and affected patients at evaluated dose levels |
| **Secondary Objectives** | **Secondary Endpoints** | **Estimators** |
| • To assess evidence of improvement and resolution of ARDS following infusion with agenT-797<br>• To assess avoidance of multiorgan dysfunction syndrome | • Assessment of ventilator-free days<br>• Time to extubation<br>• Time to intubation in patients with mild ARDS<br>• Vital signs and physical examination findings<br>• Lung injury score (LIS): The LIS is a composite 4-point scoring system including the $PaO_2/FiO_2$, positive end-expiratory pressure (PEEP), quasi-static respiratory compliance, and the extent of infiltrates determined by imaging<br>• Intensive care unit–free days<br>• Mean daily sequential organ failure assessment score(s) (SOFA)<br>• Supportive interventions used (e.g., use of prone ventilation, paralytics, pulmonary vasodilators, and other interventions affecting oxygenation)<br>• All-cause mortality rates within 30 days and at 6 months | • Proportion of ventilator-free days and the corresponding 95% CI<br>• Median time to extubation from dosing and the corresponding 95% CI<br>• Median time to intubation from dosing in patients with mild ARDs and the corresponding 95% CI<br>• Median time of intubation and the corresponding 95% CI<br>• Proportion of intensive care unit-free days and the corresponding 95% CI<br>• Listings of vital signs and physical examination<br>• Proportion of all-cause mortality within 30 days and at 6 months and their corresponding 95% CIs<br>• Plots and listings of LIS over time<br>• Plots and listings of median daily SOFA score over time |
| • To assess evolution of cytokine release syndrome (CRS) | • Change in clinical parameters and biomarkers relevant to CRS (D-dimers, ferritin, C-reactive protein, IL-1, and IL-6) | • Listings of clinical parameters and biomarkers relevant to CRS |

| | | |
|---|---|---|
| • To assess decay in quantitative (as feasible, or positive/negative) viral burden from upper and lower respiratory tract samples collected sequentially, as appropriate<br>• To assess prevention of viral reactivation | • Decay in quantitative (as feasible, or positive/negative) viral burden from upper and lower respiratory tract samples collected sequentially under treatment<br>• Time from dosing to viral clearance and determining if iNKT cells prevent re-activation of other viruses (cytomegalovirus, human papillomavirus, herpes simplex virus, Epstein-Barr virus) and fungal infections | • Plots and listings of quantitative viral burden from upper and lower respiratory tract from samples<br>• Median time from dosing to viral clearance |
| **Exploratory Objectives** | **Exploratory Endpoints** | |
| • To identify biomarkers that could predict clinical activity against severe acute respiratory syndrome-coronavirus 2 (SARS-CoV-2) | • Additional cytokine signatures and laboratory values, including, but not limited to, levels of interferon gamma, lactate dehydrogenase, tumor necrosis factor alpha, prothrombin time, partial thromboplastin time, and creatine phosphokinase, change with treatment, and if change is correlated with clinical improvement | • Plots and listings of additional cytokine signatures and laboratory values. |
| • To evaluate persistence and longevity of allogeneic iNKT cells agenT-797 with respect to tissue localization in target organs | • Presence and number of iNKT cells and other immune cells in bronchoalveolar lavage and/or microlavage fluid<br>• Persistence of allogeneic iNKT cells in circulation through direct measurement in peripheral blood and through cell free DNA as surrogate of general persistence in tissue(s) | • Number of iNKT cells and other immune cells in bronchoalveolar lavage and/or microlavage fluid<br>• Number of allogeneic iNKT cells in peripheral blood and cfDNA |
| • To evaluate development of alloimmunity against administered donor cells | • Presence of alloantibodies to major histocompatibility (MHC) Class I and MHC Class II and rate of mismatch | • Listing of alloantibodies to MHC Class I and MHC class II and rate of mismatch |
| • To evaluate whether iNKT cells prevent secondary infections | • Presence of iNKTs cells in peripheral blood and risk of secondary infections (e.g., *Pseudomonas* | • Number of iNKT cells in peripheral blood<br>• Proportion of patients who developed secondary |

| | | |
|---|---|---|
| | *aeruginosa, Klebsiella pneumoniae,* and fungal infections) | infections during follow-up period |
| • To evaluate whether iNKT cells improve lung fitness through assessments of respiratory function | • Clinical and respiratory function assessments<br>• Change in pulmonary involvement based on X-ray and/or computed tomography (CT) scans, as available<br>• Health of lung epithelial cells (RNA analysis) | • Listing of clinical and respiratory function assessments<br>• Listing of change in pulmonary involvement<br>• Listing of health of lung epithelial cells |
| • To explore whether iNKT cells promote viral resolution without lung fibrosis in SARS-CoV-2 (COVID-19) patients | • Time from dosing to viral clearance<br>• Fibrosis signs based on X-ray and/or CT scans and respiratory function assessments | • Median time from dosing to viral clearance<br>• Listing of fibrosis signs<br>• Listing of respiratory function assessments |

# 4 STATISTICAL METHODS

## 4.1 Data Quality Assurance

All tables, figures and data listings to be included in the report will be independently checked for consistency, integrity and in accordance with standard procedures.

## 4.2 General Presentation Considerations

Baseline is defined as the last available pre-treatment assessment. The end of the study is defined as the date on which the last subject completes the final follow-up visit, which may be up to 6 months after the last subject's screening date. Treatment day will be calculated relative to the date of the first treatment day such that Treatment Day = Assessment Date – First Infusion Date + 1. 6

Continuous data will be summarized in terms of the mean, standard deviation (SD), median, upper quartile, lower quartile, minimum, maximum and number of observations, unless otherwise stated. The minimum and maximum will be reported to the same number of decimal places as the raw data recorded in the database. The mean, median, lower quartile and upper quartile will be reported to one more decimal place than the raw data recorded in the database. The SD will be reported to two more decimal places than the raw data recorded in the database. In general, the maximum number of decimal places reported shall be four for any summary statistic.

Categorical data will be summarized in terms of the number of patients providing data at the relevant time point (n), frequency counts and percentages.

Percentages will be presented to one decimal place. Percentages will not be presented for zero counts.

Changes from baseline in categorical data will be summarized using shift tables where appropriate.

P-values greater than or equal to 0.001, in general, will be presented to three decimal places. P-values less than 0.001 will be presented as "<0.001".

Wilson confidence intervals (CIs) will be presented to one more decimal place than the raw data.

## 4.3 Software

All report outputs will be produced using SAS version 9.4 or later or R version 4.1.1 or later.

## 4.4 Study Patients

### 4.4.1 Disposition of Patients

A clear accounting of the disposition of all patients who enter the study will be provided, from screening to study completion based on all subject analysis set, as defined in Section 4.5.

The disposition summaries will include the following:

- Number and percentage of patients screened for entry into the study.
- Number and percentage of patients excluded by major reason.
- Number and percentage of patients treated (with at least one dose of study medication).
- Number and percentage of patients entering; withdrawing from study treatment; withdrawing from the study and completing each phase of the study by dose level and overall. Withdrawals from the study and from study treatment will also be summarized by major reason.

A by-subject listing of eligibility details, visit dates and withdrawal/study completion details (including reason for discontinuation and duration) will be provided.

### 4.4.2 Protocol Deviations

Major protocol deviations are defined as those deviations from the protocol likely to have an impact on the efficacy and/or safety of study treatments. The impact of the major protocol deviations on the efficacy and/or safety results will be investigated by assessing the robustness of the study results and conclusions to the choice of analysis set, both including and excluding data potentially affected by major protocol deviations.

Major protocol deviations and any action to be taken regarding the exclusion of patients or affected data from specific analyses are defined in the project-specific protocol deviation specification.

All protocol deviations will be listed by subject.

## 4.5 Analysis Sets

Three subject analysis sets are defined for the study analyses, as follows:

- **The All Subject Analysis Set** will include all patients who have completed informed consent, irrespective of the study treatment administered. This analysis set will be used for the disposition summary and the listing of study populations.
- **The Safety Analysis Set** will include all patients who received agenT-797. This analysis dataset will be used for safety analysis and all listings other than listings of subject disposition and study populations.
- **The Dose-Limiting Toxicity Analysis Set** will include all patients who either experienced a DLT within 28-day observation or completed study treatment and 28-

day observation for DLT without experiencing DLT. The DLT analysis set will be used for the analysis of DLT rates and for determining MTD.

## 4.6 Demographics and Baseline Characteristics

Demographic and baseline characteristics will include age, sex, race, ethnicity, height, weight, body mass index (BMI) and serum pregnancy test results for all women of childbearing potential based on the safety set, by dose group and overall.

Age will be derived from patients' date of birth as the number of complete years between a subject's birth date and the date of informed consent.

Summary tables will be presented for both continuous variables (age, height, weight and BMI) and categorical variables (sex, race, ethnicity and serum pregnancy test results) using frequency and percentages.

A by-subject listing of the demographics and baseline characteristics will be provided.

## 4.7 Diagnosis and Extent of Disease

Summary statistics will be tabulated for the diagnosis and extent of the disease based on the safety set, by dose group, and overall. This analysis will include the following:

- Time since initial diagnosis
- Peripheral blood sample tests at study entry
- Urine sample tests at study entry

A by-subject listing of the diagnosis and extent of the disease will be provided.

## 4.8 Medical History and Concomitant Illnesses

All medical history, concomitant conditions and symptoms entered on the electronic case report form (eCRF) will be coded by system organ class (SOC) and preferred term (PT) using the Medical Dictionary for Regulatory Activities (MedDRA) version 22.0 or higher based on the safety analysis set.

All medical history and concomitant illnesses will be listed by subject.

## 4.9 Prior and Concomitant Medications

All prior and concomitant medications will be based on the safety set.

Medication start and stop dates will be compared to the date of first dose of study medication to allow medications to be classified as either prior only, both prior and concomitant, or concomitant only. Medications starting after the completion/withdrawal date will be listed but will not be classified or summarized.

Medications that start and stop prior to the date of first dose of study medication will be classified as prior only. If a medication starts before the date of first dose of study medication and stops on or after the date of first dose of study medication, then the medication will be classified as both prior and concomitant. Medications will be classified as concomitant only if they have a start date on or after the date of first dose of study medication.

If medication start and/or stop dates are missing or partial, the dates will be compared as far as possible with the date of first dose of study medication. Medications will be assumed to be concomitant only, unless there is clear evidence (through comparison of partial dates) to suggest that the medication started prior to the first dose of study medication. If there is clear

evidence to suggest that the medication started prior to the first dose of study medication, the medication will be assumed to be both prior and concomitant, unless there is clear evidence to suggest that the medication stopped prior to the first dose of study medication. If there is clear evidence to suggest that the medication stopped prior to the first dose of study medication, the medication will be assumed to be prior only.

A by-subject listing will be listed for each patient.

## 4.10 Treatment Exposure / Compliance

### 4.10.1 Treatment Exposure

AgenT-797 will be administered as a single intravenous infusion of allogeneic iNKT cells over 10 to 30 minutes on Day 1. The extent of the exposure to agenT-797 will be summarized and presented based on the safety analysis set, by dose level and overall. The summary will include:

- Duration of agenT-797 administration, defined as the number of minutes between the administration start time and the end time
- Calculated dose level
- Actual dose level

A by-subject listing of exposure data will be provided.

### 4.10.2 Compliance

AgenT-797 should only be used as directed in this protocol and as detailed in the Pharmacy Manual. Details of treatment with agenT-797 for each subject will be recorded in the eCRF.

## 4.11 Efficacy Evaluation

### 4.11.1 Analysis and Data Conventions

No formal testing of hypotheses has been planned in this study. All analyses will be based on descriptive statistics.

#### 4.11.1.1 Handling of Dropouts or Missing Data

No imputation will be made for missing data resulting from an early withdrawal, missed evaluations, or any other unforeseen reason. All missing or partial data will be presented in the patient data listings as they are recorded on the eCRF.

#### 4.11.1.2 Interim Analyses

Interim analyses may be performed as appropriate and will be informal, descriptive and exploratory in nature, without any formal statistical testing.

### 4.11.2 Efficacy Variables

The analysis of clinical activity will be performed on the safety analysis set.

The following clinical responses will be investigated.

#### 4.11.2.1 Intubation/continuation/discontinuation of intubation/mechanical ventilation

The time between dose and intubation, time of continuation and then time between dose and extubation can be used to measure the efficacy of the therapy.

The following parameters will be computed together with their 95% CI:

- Median time between dose and intubation
- Median time between dose and extubation
- Medium intubation continuation time

#### 4.11.2.2 Supportive oxygenation intervention

The length of time of oxygenation intervention is a good indication of efficacy. The median time for oxygenation intervention will be computed.

#### 4.11.2.3 Baseline and every 12 hours as feasible: C-reactive protein, D-dimers, ferritin, IL-1, IL-6 to assess CRS evolution

A longitudinal analysis of all these biomarkers will be done to assess CRS evolution. Longitudinal plots for these variables for each patient will be provided.

#### 4.11.2.4 All-case mortality

Proportion of death from all causes within 30 days and at 6 months and its 95% score CI will be calculated. It is a direct measurement of efficacy of the therapy.

#### 4.11.2.5 Decrease in viral burden from upper respiratory track samples collected sequentially under treatment

A longitudinal analysis and plot of the viral burden from upper respiratory track samples collected of each patient will be provided.

#### 4.11.2.6 Persistence of allogeneic iNKT cells

A longitudinal analysis and plot of the amount of allogeneic iNKT cells will be provided.

#### 4.11.2.7 Baseline and every 24 hours: complete blood count with differential, comprehensive metabolic panel, procalcitonin, B-type natriuretic peptide, fibrinogen, prothrombin time, partial thromboplastin time, magnesium, angiotensin II level

Longitudinal analyses and plots of all these biomarkers will be provided.

#### 4.11.2.8 Available image (X-ray, CT-scans) to evaluate ARDS

Longitudinal analyses and plots for every measurement based on available image to evaluate ARDS will be provided.

#### 4.11.2.9 Change in lung fitness through assessment of respiratory function

A longitudinal analysis and plots of a respiratory functional score (LIS score) will be provided.

#### 4.11.2.10 Time from dosing to viral clearance

Median time from dosing to viral clearance will be estimated.

##### 4.11.2.11 Prevention of secondary infections

Proportion of patients with developed secondary infections will be computed together with its 95% score CI.

##### 4.11.2.12 SOFA score

The SOFA score(s) will be determined for each patient at specific timepoint as specified in Table 4, Schedule of Assessment, and will be used to assess the presence, number, and severity of organ dysfunction in six organ systems.

##### 4.11.2.13 Measurement of allo antibodies against HLA class 1 and class 2 molecules

The presence of panel reactive antibodies (PRA) will be determined for HLA class I and HLA class II by flow cytometry. The ID of the PRA detected in patient serum samples will be correlated with the known donor tissue type by virtual crossmatch, to determine the presence of Donor Specific Antibodies (DSA) in patient serum. The presence of PRA and DSA in patient serum will be determined pre infusion of agent-797, and at specific timepoints post infusion, as defined in Table 4, the Schedule of Assessment.

##### 4.11.2.14 RNA analysis of epithelial cells

The health of epithelial cells will be evaluated by an RNA analysis at specific timepoints as defined in Table 4, the Schedule of Assessment.

#### 4.11.3 Safety Evaluation

The DLT analysis set will be used for the DLT rate and MTD of agenT-797. The safety analysis set will be used for all other safety analyses.

#### 4.11.4 Adverse Events

An AE is any untoward medical occurrence in a study subject administered an investigational medicinal product (IMP) which does not necessarily have a causal relationship with this treatment. An AE can therefore be any unfavorable and unintended sign (including a clinically significant abnormal laboratory finding), symptom, or disease temporally associated with the use of an IMP, whether or not considered related to the IMP. Adverse events may include the onset of new illness and the exacerbation of pre-existing conditions.

Other untoward events occurring in the framework of a clinical study will be recorded as AEs, e.g. those occurring during treatment-free periods (including screening or post-treatment follow-up periods), in association with study-related procedures and assessments.

Concomitant illnesses, which existed before entry into the clinical study, will not be considered AEs unless they worsen during the treatment period. Pre-existing conditions will be recorded as part of the subject's medical history.

A treatment-emergent adverse event (TEAE) is defined as an AE that begins or that worsens in severity after at least one dose of the study drug has been administered.

Any AEs with incomplete start and end dates/times will be treated as follows:

Adverse events with unknown start and/or end times (but where the date is known) will be imputed with a time of 00:00 h for the tabulations but will be shown as NK: NK in the listings (where NK = Not Known).

Adverse events with completely unknown start dates will be considered as treatment-emergent for the tabulations and will be shown as NK in the listings. Partially complete AE dates will not be imputed in the listings and the available day, month and year components will be used to determine if an adverse event is treatment-emergent taking the first dosing date and end of study date as references.

Adverse events will be coded using the MedDRA version 22.0 or higher.

The relationship or association of the study medication in causing or contributing to the AE will be characterized by the Investigator using the following classification and criteria:

- Possibly or probably related: A clinical event, including laboratory test abnormality, with a reasonable time sequence to study medication administration that might or might not be also explained by concurrent disease or other drugs or chemicals.
- Not related: A clinical event, including laboratory test abnormality, judged to be clearly and incontrovertibly due to extraneous causes (e.g., diseases, environment), or with a temporal relationship to study medication administration that makes a causal relationship improbable, and/or for which other drugs, chemicals, or underlying diseases provide a much more plausible explanation.

Severity or intensity will be assessed according to the NCI CTCAE version 5.0 severity grading scale. Grade refers to the severity of the AE. The NCI CTCAE version 5.0 displays Grades 1 through 5 with unique clinical descriptions of severity for each AE based on this general guideline:

- Grade 1: Mild (awareness of sign or symptom, but easily tolerated)
- Grade 2: Moderate (discomfort sufficient to cause interference with normal activities)
- Grade 3: Severe (incapacitating, with inability to perform normal activities)
- Grade 4: Life-threatening (urgent intervention indicated)
- Grade 5: Death related to AE.

The following summaries will be generated based on safety analysis set by dose level and overall:

- A summary of the number and percentage of patients reporting an AE by SOC and PT.
- A summary of the number and percentage of patients reporting a TEAE by SOC and PT.
- A summary of the number and percentage of patients reporting a related TEAE by SOC and PT.
- A summary of the number and percentage of patients reporting a TEAE by severity, SOC and PT.
- A summary of the number and percentage of patients reporting a TEAE by causality, SOC and PT.

AE summaries will be ordered in terms of decreasing frequency for SOC, and PT within SOC, in the overall group, and then alphabetically for SOC, and PT within SOC.

For each subject and each adverse event, the worst severity recorded will be attributed and used in the by-severity summaries. Similarly, the worst causality (most related to treatment) will be attributed and used in the by-causality summaries. If severity or causality is missing, a conservative approach for AE assessment (taking into account the worst case) will be followed.

A by-subject listing of all AEs (including non-treatment-emergent events) will be provided. This listing will be presented by dose level and will include center, subject identifier, age, sex, race, adverse event (SOC, PT, and verbatim term), date of onset, date of resolution, duration, severity, seriousness, action taken, outcome and causality.

### 4.11.5 Deaths, Serious Adverse Events, and Other Significant Adverse Events

A serious AE (SAE) is an AE that fulfils one or more of the following criteria:

- Results in death.
- It is immediately life-threatening.
- It requires inpatient hospitalization or prolongation of existing hospitalization.
- It results in persistent or significant disability or incapacity, or substantial disruption of the ability to conduct normal life functions.
- Is or results in a congenital abnormality or birth defect.

Other significant AEs are those reported as leading to an intervention e.g. withdrawal from study, discontinuation of study treatment, introduction of concomitant medication, dose reduction.

CRS is a systemic inflammatory response characterized by a spectrum of severity, ranging from flu-like symptoms (i.e., fever, myalgia, nausea/vomiting), to hypotension, respiratory and renal failure, and uncontrolled coagulopathy. The onset of CRS can typically occur between 24 hours and 14 days after the completion of T cell administration. Therefore, after agenT-797 infusion, should CRS occur, it should be managed by the Investigator per institutional guidelines.

The American Society for Transplantation and Cellular Therapy (ASTCT) have published consensus guidelines for grading of CRS (Lee 2019). The ASTCT consensus grading guidelines are presented in

Table 3 and align with management algorithms developed by Lee and colleagues (Riegler 2019), which are to be used as a guide for the Investigator in the assessment of CRS.

**Table 3. ASTCT CRS Consensus Grading**

| Parameter | Grade 1 | Grade 2 | Grade 3 | Grade 4 |
|---|---|---|---|---|
| **Fever*** | Temperature ≥ 38°C | Temperature ≥ 38°C | Temperature ≥ 38°C | Temperature ≥ 38°C |
| | | **With** | | |
| **Hypotension** | None | Not requiring vasopressors | Requiring a vasopressor with or without vasopressin | Requiring multiple vasopressors (excluding vasopressin) |
| | | **And/or**† | | |
| **Hypoxia** | None | Requiring low-flow nasal cannula‡ or blow-by | Requiring high-flow nasal cannula,‡ facemask, nonrebreather mask, or Venturi mask | Requiring positive pressure (e.g., CPAP, BiPAP, intubation and mechanical ventilation) |

From Lee 2019.

Abbreviations: ASTCT: American Society for Transplantation and Cellular Therapy; BiPAP: bilevel positive airway pressure; CPAP: continuous positive airway pressure; CRS: cytokine release syndrome; NCI-CTCAE: National Cancer Institute Common Terminology Criteria for Adverse Events.

Organ toxicities associated with CRS may be graded according to NCI-CTCAE v5.0 but they do not influence CRS grading.

* Fever is defined as temperature ≥ 38°C not attributable to any other cause. In patients who have CRS then receive antipyretic or anti-cytokine therapy such as tocilizumab or steroids, fever is no longer required to grade subsequent CRS severity. In this case, CRS grading is driven by hypotension and/or hypoxia.

† CRS grade is determined by the more severe event: hypotension or hypoxia not attributable to any other cause. For example, a patient with temperature of 39.5°C, hypotension requiring 1 vasopressor, and hypoxia requiring low-flow nasal cannula is classified as Grade 3 CRS.

‡ Low-flow nasal cannula is defined as oxygen delivered at ≤ 6 L/min. Low flow also includes blow-by oxygen delivery, sometimes used in pediatrics. High-flow nasal cannula is defined as oxygen delivered at > 6 L/min.

The most common anticipated potential toxicities resulting from treatment with agenT-797 are outlined in Section 7.4.1 of the protocol. Some of these possible AEs have also been further defined as AEs of special interest (AESI):

- Cytokine release syndrome (CRS) Grade ⩾ 2 (as per the American Society for Transplantation and Cellular Therapy [ASTCT] Consensus, in Table 3).
- Hypersensitivity or infusion-related reactions Grade ⩾ 2 (as per NCI CTCAE version 5.0) directly post-administration of agenT-797.
- Acute GVHD any stage as per the national/local/institutional guidelines.
- Immune effector cell-associated neurotoxicity syndrome (ICANS) Grade ⩾ 2 (as per the ASTCT Consensus).

The following summaries will be generated based on safety analysis set by dose level and overall:

- A summary of the number and percentage of deaths during the study.
- A summary of the number and percentage of patients reporting a serious TEAE by SOC and PT.
- A summary of the number and percentage of patients with AEs leading to discontinuation of study treatment by SOC and PT.
- A summary of the number and percentage of patients with AESIs leading to discontinuation of study treatment by SOC and PT.

A by-subject listing will be provided for the following events:

- Deaths that occurred during the study.
- Serious TEAE.
- AEs leading to discontinuation of study treatment.
- AESIs leading to discontinuation of study treatment.

### 4.11.6 Dose-Limiting Toxicity

The following summaries will be generated based on the DLT analysis set by dose level and overall:

- A summary of the number and proportion of patients who experience a DLT during the DLT evaluation period.
- A summary of the number and proportion of TEAEs experienced by patients during the DLT evaluation period.

### 4.11.7 Clinical Laboratory Evaluation

All lab parameters listed will be collected and analyzed at the time points detailed in the schedule of assessments in Appendix 1.

The following laboratory assessments will be analyzed:

- Hematology: hemoglobin, hematocrit, platelet count, red blood cell count, white blood cell count, mean corpuscular volume, mean corpuscular hemoglobin, absolute neutrophils count, absolute lymphocytes count, absolute monocytes count, absolute eosinophils count, absolute basophils count, absolute bands count, percentage of neutrophils, percentage of lymphocytes, percentage of monocytes, percentage of eosinophils, percentage of basophils and percentage of bands.
- Coagulation: prothrombin time, activated partial thromboplastin time and international normalized ratio.
- Clinical chemistry: albumin, alkaline phosphatase, alanine aminotransferase, aspartate aminotransferase, blood urea nitrogen, urea, calcium, carbon dioxide, chloride, C-reactive protein, creatinine, creatinine clearance, ferritin, glucose, lactate dehydrogenase, magnesium, phosphorus, potassium, sodium, total bilirubin, direct bilirubin, indirect bilirubin and total protein.
- Urinalysis: pH, ketones, specific gravity, bilirubin, protein, blood, glucose.
- Human herpes virus 6 and 7 tests.
- Human immunodeficiency virus 1 and 2, hepatitis B and C, and human T-lymphotropic virus serologies, hepatitis B virus DNA and hepatitis C virus RNA.
- Pregnancy tests.
- Circulating cytokines.

The following summaries will be generated:

- A summary of each laboratory parameter by time point.
- A summary of the change from baseline in each laboratory parameter by time point.
- A summary of the number and percentage of patients experiencing abnormal values, by laboratory parameter and time point.
- A summary of the number and percentage of patients experiencing low, normal or high values at baseline and at selected post-baseline time points, by laboratory parameter (shift table).

- A summary of the number and percentage of patients experiencing treatment-emergent laboratory abnormalities, by laboratory parameter.
- A summary of the number and percentage of patients experiencing treatment-emergent clinically significant laboratory abnormalities, by laboratory parameter.

The observed values and change from baseline of the safety laboratory assessments will be summarized using descriptive statistics showing the number of observations (n), mean, median, SD, minimum, and maximum value. Categorical results will be summarized based on the number and percentage in each category, presented by time point.

Laboratory results outside the normal range will be flagged. The abnormal values will be flagged with ‘L’ (low) for values below the lower limit of the laboratory’s normal range or ‘H’ (high) for values above the upper limit of the laboratory’s normal range. Abnormal values will be graded as not clinically significant (NCS) or clinically significant (CS). Clinically significant laboratory values will be recorded by the investigator as AEs.

A by-subject listing of all laboratory data will be provided with abnormal values highlighted.

### 4.11.8 Vital Signs, Physical Findings and Other Observations Related to Safety

Physical examination, vital signs, and electrocardiogram (ECG) will be collected at the time points detailed in the schedules of assessments in Appendix 1.

#### 4.11.8.1 Physical examination

A complete physical examination will be performed at screening and will include assessment of height (during screening only) and weight and vital signs, examination of head, eyes, ears, nose, throat, neck, cardiovascular, chest/lungs, abdomen (including liver and spleen size), extremities, neurological, skin, and lymph nodes, as well as a brief neurologic examination.

Clinically significant physical exam findings will be summarized and listed.

#### 4.11.8.2 Vital signs

Vital signs include body temperature, heart rate, respiratory rate, systolic and diastolic blood pressure. Vital signs are measured by the physician or the nurse and are part of the complete physical examination until Day 29 and measured each time it is clinically indicated.

A summary of each vital sign parameter and its change from baseline will be generated.

A by-subject listing will be provided with the reference ranges.

#### 4.11.8.3 ECG

A single, 12-lead ECG will be performed. The ECG will be evaluated by the Investigator as ‘Normal’, ‘Abnormal, NCS’ or ‘Abnormal, CS’.

A summary of each ECG parameter will be generated.

A by-subject listing will be provided.

## 5 REFERENCE

[1] ARDS Definition Task Force. Acute Respiratory Distress Syndrome: The Berlin Definition. *JAMA*. 2012;307(23):2526-2533.

[2] Exley MA, Friedlander P, Alatrakchi N, et al. Adoptive transfer of invariant NKT cells as immunotherapy for advanced melanoma: A Phase I clinical trial. Clin Cancer Res. 2017;23(14):3510-9.

[3] SOFA score accessed on 11 Nov 2021 from https://www.mdcalc.com/sequential-organ-failure-assessment-sofa-score

[4] Lee DW, Santomasso BD, Locke FL, et al. ASTCT Consensus Grading for Cytokine Release Syndrome and Neurologic Toxicity Associated with Immune Effector Cells. *Biol Blood Marrow Transplant.* 2019;25(4):625-638.

[5] Riegler LL, Jones GP, Lee DW. Current approached in the grading and management of cytokine release syndrome after chimeric antigen receptor T-cell therapy. *Ther Clin Risk Manag.* 2019;15:323-335

# 6 Appendix

## APPENDIX 1

### Table 4. Schedule of Assessments

| **Assessment** | **Screening** | **Treatment Period** | | | | | | | | | | **EOH[a]** | **30-Day F/U** |
|---|---|---|---|---|---|---|---|---|---|---|---|---|---|
| **Study Day** | **± 4** | **1** | **2** | **4** | **6** | **7** | **10 ± 1** | **14 ± 1[b]** | **21 ± 2[b]** | **28 ± 2[b]** | **32 to EOH[b]** | **Day of discharge** | **30 ± 2** |
| Informed consent | X[c] | | | | | | | | | | | | |
| Inclusion/exclusion | X[c] | | | | | | | | | | | | |
| HLA serotype | X[c] | | | | | | | | | | | | |
| Medical history & demographics | X[c] | | | | | | | | | | | | |
| Physical examination[d] | X[c] | Daily monitoring/assessments | | | | | | | | | | X | X |
| Vital signs[e] | X[c] | Daily monitoring/assessments | | | | | | | | | | X | X |
| ECG[f] | X[c] | X | | | | | | | | X | | X | X |
| Radiological evaluation (X-ray/CT) | X[c] | X | | | | | | | | | | X | |
| Concomitant medications & procedures | X | Continuously collected | | | | | | | | | | | |
| Adverse events[g] | X | Continuously collected | | | | | | | | | | | |
| Hematology[h] | X[c] | Daily monitoring/assessments | | | | | | | | | | X | X |
| Coagulation[h] | X[c] | X | | X | | X | X | X | X | X | | X | X |
| Clinical chemistry[h] | X[c] | Daily monitoring/assessments | | | | | | | | | | X | X |
| Urinalysis[i] | X[c] | X | | X | | X | X | X | X | X | | X | X |
| Pregnancy test (WOCBP)[j] | X | | | | | | | | | | | | |
| Serum for cytokine analyses[k] | X[c] | X | | X | | X | X | X | X | X | | X | |
| Whole blood for persistence of agenT-797 (peripheral persistence) | X[c] | X[l] | X | X | X | X | X | X | X | X | | X | X |
| Whole blood for cfDNA (tissue persistence) | X[c] | X[m] | X | X | X | X | X | X | X | X | | X | X |
| Bronchoalveolar lavage (iNKT cell | X[c] | X | | X | | X | X | X | X | X | | X | |

| Assessment | Screening | Treatment Period | | | | | | | | | | EOH[a] | 30-Day F/U |
|---|---|---|---|---|---|---|---|---|---|---|---|---|---|
| **Study Day** | **± 4** | **1** | **2** | **4** | **6** | **7** | **10 ± 1** | **14 ± 1[b]** | **21 ± 2[b]** | **28 ± 2[b]** | **32 to EOH[b]** | **Day of discharge** | **30 ± 2** |
| presence and biomarker)[k,n] | | | | | | | | | | | | | |
| Epithelial sampling by bronchial protected specimen brush (RNA analysis)[o] | X[c] | X | | X | | X | X | X | X | X | | X | |
| Serum for alloantibodies | | X[c] | | | | | | X | | | | X | |
| agenT-797 cell infusion | | X | | | | | | | | | | | |
| Arterial blood gas | X | Daily monitoring/assessments as needed | | | | | | | | | | X | X |
| Symptoms of ARDS[p] evaluation (Berlin definition)[q] | X | Daily monitoring/assessments as needed | | | | | | | | | | X | X |
| SOFA score[r] | X | Daily monitoring/assessments as needed | | | | | | | | | | X | |
| Viral burden assessment (swab), or endotracheal suction as feasible (RT-PCR) | X[s] | X | X | X | X | X | X | X | X | X | | X | |
| Viral shedding assessment as feasible | | X | | | | | | | | | | X | |
| D-dimer, CPK, cardiac troponins, and ferritin | | Every 12h, as feasible | | | | | | | | | | X | |

Abbreviations: AE: adverse events; AESI: adverse events of special interest; ARDS: acute respiratory distress syndrome; d: day(s); cfDNA: cell free DNA; CPK: creatine phosphokinase; CT: computed tomography; ECG: electrocardiogram; EOH: End-of-Hospitalization; F/U: Follow-up; HLA: human leukocyte antigen; h: hour (s); IL: interleukin; iNKT cells: invariant natural killer T cells; RT-PCR: reverse transcriptase polymerase chain reaction; SAE: serious adverse event; SOFA: sequential organ failure assessment; WOCBP: women of childbearing potential.

[a] End-of-Hospitalization is defined as patient discharge from hospital and/or death. If a patient is discharged at Day 30, 30-day Follow-up will be the same as EOH. After discharge, a patient will enter Safety Follow-up period and the patient will be contacted by telephone 3 and 6 months after discharge regarding any SAEs and to confirm survival, as feasible..

[b] In case of earlier discharge from the hospital, no visit is expected until 30-Day Follow-up.

[c] Performed before infusion of agenT-797 on D1. These samples will be obtained at the time of the routine labs blood draws.

[d] A complete examination, as feasible, will be performed at Screening and symptom-directed and medically indicated physical examinations are done thereafter.

[e] Vital signs include body temperature, heart rate, respiratory rate, systolic and diastolic blood pressure and are assessed by physician or nurse. On the day of infusion of agenT-797 (D1), measurements will be 15 min prior to infusion, and 15 min, 30 min, 1h, 2h, 3h, and 4h postinfusion.

[f] ECG to be performed at Screening, Day 1 predose and EOH, 30-Day Follow-up, and at other times as clinically indicated.

[g] Only AEs associated with study procedures and/or leading to withdrawal and all SAEs will be collected prior to study drug infusion. Patients are monitored for toxicities following infusion. All other AEs, SAEs, and AESIs will be collected in the Safety Follow-up period.

[h] Hematology/coagulation/clinical chemistry: Complete blood count with automated and/or manual differential will be collected daily, as feasible. Coagulation: activated partial thromboplastin time and international normalized ratio. Clinical chemistry: includes but not limited to albumin, alkaline phosphatase, alanine aminotransferase, aspartate aminotransferase, blood urea nitrogen, urea, calcium, chloride, C-reactive protein, creatinine, glucose, lactate dehydrogenase, magnesium, phosphate/phosphorus, potassium, sodium, total bilirubin, direct and indirect bilirubin, total protein, and beta-2-microglobulin and will be collected daily, as feasible. All will be collected at EOH and at 30-Day Follow-up.

[i] Urinalysis: pH, ketones, specific gravity, bilirubin, protein, blood, and glucose. Will be done at the days indicated and at EOH and at 30-Day Follow-up.

[j] Pregnancy testing for women of child-bearing potential: serum test at Screening.

[k] Cytokines/biomarkers: D1 – preinfusion, end of infusion, and at 2, 4, and 8 hours postinfusion agenT-797, and as feasible thereafter. Cytokines for analysis may include but are not limited to: epidermal growth factor, eotaxin, fibroblast growth factor-basic, granulocyte-colony stimulating factor, granulocyte-macrophage colony stimulating factor, hepatocyte growth factor, interferon-alpha, interferon-gamma, IL-1 beta, IL-1 alpha, IL-1RA, IL-2, IL-2R, IL-3, IL-4, IL-5, IL-6, IL-7, IL-8, IL-9, IL-10, IL-12 (p40/p70) IL-13, IL-15, IL-17A, IL-17F, IL-22, IP-10, monocyte chemoattractant protein-1, MIG, MIP-1 alpha, MIP-1 beta, Regulated upon Activation, Normal T Cell Expressed and Presumably Secreted, tumor necrosis factor-alpha, vascular endothelial growth factor, and matrix metallopeptidase-9.

[l] To be obtained 5, 15, and 30 min, and 1, 2, and 4 hours postinfusion.

[m] To be obtained 2 hours postinfusion.

[n] To be obtained in intubated patients if clinically indicated using microlavage techniques when possible.

[o] To be obtained in intubated patients when possible.

[p] Evaluations including physical examination and procedures required for lung injury score evaluation (chest X-ray, $PaO_2/FiO_2$ ratio, positive end-expiratory pressure, compliance, mL/cm $H_2O$) as needed, including daily or more often until recovery (ARDS 2012).

[q] As needed.

[r] SOFA score will be calculated using results of the following tests: $PaO_2$, $FiO_2$, platelet count, need for mechanical ventilation, Glasgow Coma score, bilirubin, mean arterial pressure or use of vasoactive medications, creatinine, and diagnosis of COVID-19 (SOFA).

[s] Patients must be SARS-CoV-2 or influenza positive at diagnosis; refer to Section 4.7.1 of the study protocol
