# Supplementary material for: A phase 1/2 clinical trial of invariant natural killer T cell therapy in moderate-severe acute respiratory distress syndrome
Source: Nat Commun. 2024 Feb 6;15:974. doi: 10.1038/s41467-024-44905-z (PMC10847411; doi:10.1038/s41467-024-44905-z)
Supplement: Supplementary file 3 — Reporting Summary [file 41467_2024_44905_MOESM3_ESM.pdf]

nature portfolio

Corresponding author(s): Justin Stebbing, e mail: justin.stebbing@aru.ac.uk

Last updated by author(s): Dec 29, 2023

Reporting Summary

Nature Portfolio wishes to improve the reproducibility of the work that we publish. This form provides structure for consistency and transparency in reporting. For further information on Nature Portfolio policies, see our Editorial Policies and the Editorial Policy Checklist.

Statistics

For all statistical analyses, confirm that the following items are present in the figure legend, table legend, main text, or Methods section.

n/a	Confirmed	
☐	☒	The exact sample size (*n*) for each experimental group/condition, given as a discrete number and unit of measurement
☐	☒	A statement on whether measurements were taken from distinct samples or whether the same sample was measured repeatedly
☐	☒	The statistical test(s) used AND whether they are one- or two-sided<br>*Only common tests should be described solely by name; describe more complex techniques in the Methods section.*
☐	☒	A description of all covariates tested
☐	☒	A description of any assumptions or corrections, such as tests of normality and adjustment for multiple comparisons
☐	☒	A full description of the statistical parameters including central tendency (e.g. means) or other basic estimates (e.g. regression coefficient) AND variation (e.g. standard deviation) or associated estimates of uncertainty (e.g. confidence intervals)
☐	☒	For null hypothesis testing, the test statistic (e.g. *F*, *t*, *r*) with confidence intervals, effect sizes, degrees of freedom and *P* value noted<br>*Give P values as exact values whenever suitable.*
☒	☐	For Bayesian analysis, information on the choice of priors and Markov chain Monte Carlo settings
☒	☐	For hierarchical and complex designs, identification of the appropriate level for tests and full reporting of outcomes
☒	☐	Estimates of effect sizes (e.g. Cohen's *d*, Pearson's *r*), indicating how they were calculated

Our web collection on statistics for biologists contains articles on many of the points above.

Software and code

Policy information about availability of computer code

Data collection	No software or code was used
Data analysis	No software was used (see flow cytometry section), apart from standard desktop analytics (Microsoft Excel)

For manuscripts utilizing custom algorithms or software that are central to the research but not yet described in published literature, software must be made available to editors and reviewers. We strongly encourage code deposition in a community repository (e.g. GitHub). See the Nature Portfolio guidelines for submitting code & software for further information.

Data

Policy information about availability of data

All manuscripts must include a data availability statement. This statement should provide the following information, where applicable:

- Accession codes, unique identifiers, or web links for publicly available datasets
- A description of any restrictions on data availability
- For clinical datasets or third party data, please ensure that the statement adheres to our policy

All data are available in the main text, the supplementary materials and published posters referenced in the paper.

Human research participants

Policy information about studies involving human research participants and Sex and Gender in Research.

Reporting on sex and gender	This information has been collected and presented. Findings apply to all sex and genders.<br><br>Here, agenT-797 (3 patients at 100 million, 4 at 300 million, 14 at 109 iNKT cells, as a single infusion) was administered to 21 mechanically ventilated patients (20 on the main trial, Table 1, plus a further critically unwell individual who received an Emergency Use Authorisation (EUA), IND number 29183; see CONSORT flow diagram, Figure S2) without any dose-limiting toxicities, including 5 individuals receiving VV-ECMO (Table S1 for VV-ECMO set). Dosing was rapidly escalated as no dose-limiting treatment-emergent adverse events (TEAE) were observed, with evidence of CRS absent. Most reported TEAEs were grade 1, 2 and consistent with severe coronavirus disease-2019 (COVID-19)/ARDS (Table 2).<br><br>The median age of the main trial cohort measured 66.5 years (range 26-77 years), the average BMI at 31.8 kg/m2, the male:female sex distribution 1:1 (Table 1); all patients were intubated, on mechanical ventilation. Of the 20 treated patients, 14 (70%) survived (Figure S3A), compared to 10% in a comparative control (n=20) evaluated at the same institution. Though increased BMI is a risk factor for adverse outcomes after infection with SARS-CoV-2, no significant difference in BMI between patients who survived (average 32.1; range 16.6-51.7) and those who died due to COVID-19 (average 31.2; range 19.4-40.2) was observed.
Population characteristics	As above, a total of 20 individuals with severe COVID-19 and receiving intensive care, were dosed in 3 cohorts between July 2020 and June 2021 (Table 1) and one additional 21-year-old patient was treated under an EUA (EUA patient) for carbapenem-resistant Pseudomonas aeruginosa pneumonia requiring VV-ECMO salvage after clearing SARS-CoV-2 infection (therapeutic schematic at Figure S4).<br><br>As treatments were being developed in such sick individuals with this 'new disease' in real time, it is difficult to ascribe adverse events to treatment(s) or the disease itself. In our clinical experience, agenT-797 appeared well tolerated, with the majority of adverse events (AEs) consistent with underlying disease. Most reported TEAEs were grade 1, 2 and consistent with severe COVID-19/ARDS including anemia (n=8), fever (n=7), and acute kidney injury (n=6). One patient experienced a possible grade 4 TEAE of dyspnea (Table 2), again difficult to ascribe this to treatment during a pandemic.<br><br>There were 2 urinary tract infections noted on the day of infusion which resolved within 3 and 16 days respectively. There was one pulmonary infection (Methicillin Resistant Staphyloccocus Aureus, MRSA) noted on the day of infusion, which resolved within 5 days of infusion. All other infections secondary infections occurred post infusion of agenT-797. There was a potential dose-dependent reduction in secondary infections observed, including an over 80% reduction in pneumonia at dose cohort 3 (15%) compared to combined numbers of cohorts 1 and 2 (71%) (Table S2). Although highly intriguing and consistent with reported anti-infectious properties of iNKT cells (refs 1-4), numbers are small with respect to these conclusions. We rapidly escalated to the highest dose cohort, as we again we considered there to be a lack of toxicity related to agenT-797. Indeed, the EUA patient was treated at the highest dose level and their carbapenem-resistant Pseudomonas pneumonia rapidly cleared post agenT-797 infusion, supporting our view that agenT-797 might be able to assist in the control of secondary infections.<br><br>Of the 21 agenT-797 treated patients (including the EUA patient), 5 were given agenT-797 during VV-ECMO, traditionally regarded as 'the most aggressive salvage therapy' for critically ill individuals with COVID-19 (with one systematic review showing a 41% mortality in VV-ECMO treated patients, as per ref 22). Survival of the VV-ECMO cohort was 80% (4/5) at 30 and 90 days and 60% (3/5) at 120 days. This compares favorably to overall survival of 51% (18/35) for patients with COVID-19 treated with VV-ECMO in the same institution during the same timeframe (January 2021 to January 2022, Figure S3B, Table S1). In patients treated with agenT-797, we did not observe cell therapy-associated oxygenator failure due to clotting in filters, as reported routinely with mesenchymal stem cell therapy in ARDS patients on VV-ECMO23. To our knowledge agenT-797 represents the first use of immune cell therapy in patients receiving VV-ECMO.
Recruitment	This information has been collected and presented. This was a phase 1/2 study to evaluate the safety and potential efficacy of agenT-797, an unmodified, allogeneic iNKT cell therapy, in participants with moderate to severe acute respiratory distress syndrome (ARDS) secondary to SARS-CoV-2 or influenza, either with intubation or at high risk to be intubated, as determined using Berlin definition (as per ref 44);trial registration NCT04582201. This was a standard 3+3 dose escalation, and all participants received a single infusion of agenT-797 in doses of 100x, 300x, 1000× 106 cells. The protocol is included (supplementary file 1) and appropriate informed consent was obtained from the patient or their duly informed representatives. The study protocol, including the use of patient material, was approved by the institutional review boards (IRB) at each clinical site.<br><br>The primary outcome measures assessed safety (adverse events and dose-limiting toxicities) and secondary measures included: change from baseline in CRP and the number of participants experiencing viral reactivation and fungal infections and the evolution of CRS. Patients were recruited from Weill Cornell Medical College, Norton Cancer Center, and Providence Saint John's Health Center. Patients were enrolled between October 2020 through December 2021. The last trial patient's last visit was June 2022. The EUA patient (IND 29183) was discharged home 1/2023. Last follow up was 7/2023.
Ethics oversight	This is presented alongside the full study protocol. Each institution provided full ethics approval. Overall approval was provided by the Saint John's Cancer Institute Clinical Trials Review Committee as part of the Providence Human Research Protection Program (HRPP).

Note that full information on the approval of the study protocol must also be provided in the manuscript.

Field-specific reporting

Please select the one below that is the best fit for your research. If you are not sure, read the appropriate sections before making your selection.

☒ Life sciences ☐ Behavioural & social sciences ☐ Ecological, evolutionary & environmental sciences

For a reference copy of the document with all sections, see nature.com/documents/nr-reporting-summary-flat.pdf

Life sciences study design

All studies must disclose on these points even when the disclosure is negative.

Sample size

No sample size calculation was performed and the sample size was standard for an early phase clinical trial in accordance with the protocol. We include a statistical analysis plan alongside the trial protocol. All experiments were successfully replicated and all assays performed in triplicate as a minimum.

Descriptive statistics are used in displaying the results of the primary endpoint: adverse events and dose-limiting toxicities of agent-797 and secondary endpoints related to improvement and resolution of ARDS, evolution of CRS, avoidance of multiorgan dysfunction syndrome, as well as persistence of agenT-797. No statistical method was used to predetermine sample size, and no blinding or randomization was performed.

A treatment-emergent adverse event (TEAE) is defined as an AE that begins or that worsens in severity after the first dose of the study drug. They are coded using MedDRA version 24.1 with severity assessed according to NCI CTCAE version 5.0. Incidence rates of TEAE are summarized by preferred term (PT) including those attributed to the secondary infections. In addition, Incidence rates of TEAE of grade 3 and above, treatment related TEAE (TRAE), TRAE leading to treatment discontinuation, TRAE leading to dose interruption and TRAE leading death are summarized by cohort. For each patient and PT, the worst grade and causality (related to treatment) will be used in the summaries. Overall survival is defined as the first dose to date of death due to any cause. Kaplan Meier plot of survival is provided to show survival rates at pre-defined timepoints of 30 days and 6 months after the start of therapy. Serum cytokines are grouped into CRS markers and proinflammatory and anti-inflammatory markers and their values are plotted at pre-dose, days 1-7 and days 10-28 post dose. Descriptive p-values from ANOVA/mixed models of pre-dose and post-dose serum cytokine level comparisons are provided to show the magnitude of difference in these exploratory analyses. Plots of WBC, iNKT cells and other pharmacodynamic markers are generated to evaluate the treatment effect on these markers. No data were excluded from analysis.

Due to the small sample size in this exploratory study, results from the statistical tests are not type I error controlled or adequately powered to make any inferential statement, but rather to provide proof of concept for future studies. The statistical analysis plan is included (supplementary file 2).

Data exclusions

No data were excluded.

Replication

The experimental findings were repeated in vitro and in vivo as described in the paper. Attempts to replicate were successful.

Randomization

There was no randomisation. Patients were recruited as per the entry criteria for the trial, in each of the recruiting organisations.

Blinding

There was no blinding. This was an open label study.

Behavioural & social sciences study design

All studies must disclose on these points even when the disclosure is negative.

Study description

Briefly describe the study type including whether data are quantitative, qualitative, or mixed-methods (e.g. qualitative cross-sectional, quantitative experimental, mixed-methods case study).

Research sample

State the research sample (e.g. Harvard university undergraduates, villagers in rural India) and provide relevant demographic information (e.g. age, sex) and indicate whether the sample is representative. Provide a rationale for the study sample chosen. For studies involving existing datasets, please describe the dataset and source.

Sampling strategy

Describe the sampling procedure (e.g. random, snowball, stratified, convenience). Describe the statistical methods that were used to predetermine sample size OR if no sample-size calculation was performed, describe how sample sizes were chosen and provide a rationale for why these sample sizes are sufficient. For qualitative data, please indicate whether data saturation was considered, and what criteria were used to decide that no further sampling was needed.

Data collection

Provide details about the data collection procedure, including the instruments or devices used to record the data (e.g. pen and paper, computer, eye tracker, video or audio equipment) whether anyone was present besides the participant(s) and the researcher, and whether the researcher was blind to experimental condition and/or the study hypothesis during data collection.

Timing	*Indicate the start and stop dates of data collection. If there is a gap between collection periods, state the dates for each sample cohort.*
Data exclusions	*If no data were excluded from the analyses, state so OR if data were excluded, provide the exact number of exclusions and the rationale behind them, indicating whether exclusion criteria were pre-established.*
Non-participation	*State how many participants dropped out/declined participation and the reason(s) given OR provide response rate OR state that no participants dropped out/declined participation.*
Randomization	*If participants were not allocated into experimental groups, state so OR describe how participants were allocated to groups, and if allocation was not random, describe how covariates were controlled.*

Ecological, evolutionary & environmental sciences study design

All studies must disclose on these points even when the disclosure is negative.

Study description	*Briefly describe the study. For quantitative data include treatment factors and interactions, design structure (e.g. factorial, nested, hierarchical), nature and number of experimental units and replicates.*
Research sample	*Describe the research sample (e.g. a group of tagged Passer domesticus, all Stenocereus thurberi within Organ Pipe Cactus National Monument), and provide a rationale for the sample choice. When relevant, describe the organism taxa, source, sex, age range and any manipulations. State what population the sample is meant to represent when applicable. For studies involving existing datasets, describe the data and its source.*
Sampling strategy	*Note the sampling procedure. Describe the statistical methods that were used to predetermine sample size OR if no sample-size calculation was performed, describe how sample sizes were chosen and provide a rationale for why these sample sizes are sufficient.*
Data collection	*Describe the data collection procedure, including who recorded the data and how.*
Timing and spatial scale	*Indicate the start and stop dates of data collection, noting the frequency and periodicity of sampling and providing a rationale for these choices. If there is a gap between collection periods, state the dates for each sample cohort. Specify the spatial scale from which the data are taken*
Data exclusions	*If no data were excluded from the analyses, state so OR if data were excluded, describe the exclusions and the rationale behind them, indicating whether exclusion criteria were pre-established.*
Reproducibility	*Describe the measures taken to verify the reproducibility of experimental findings. For each experiment, note whether any attempts to repeat the experiment failed OR state that all attempts to repeat the experiment were successful.*
Randomization	*Describe how samples/organisms/participants were allocated into groups. If allocation was not random, describe how covariates were controlled. If this is not relevant to your study, explain why.*
Blinding	*Describe the extent of blinding used during data acquisition and analysis. If blinding was not possible, describe why OR explain why blinding was not relevant to your study.*

Did the study involve field work? ☐ Yes ☐ No

Field work, collection and transport

Field conditions	*Describe the study conditions for field work, providing relevant parameters (e.g. temperature, rainfall).*
Location	*State the location of the sampling or experiment, providing relevant parameters (e.g. latitude and longitude, elevation, water depth).*
Access & import/export	*Describe the efforts you have made to access habitats and to collect and import/export your samples in a responsible manner and in compliance with local, national and international laws, noting any permits that were obtained (give the name of the issuing authority, the date of issue, and any identifying information).*
Disturbance	*Describe any disturbance caused by the study and how it was minimized.*

Reporting for specific materials, systems and methods

We require information from authors about some types of materials, experimental systems and methods used in many studies. Here, indicate whether each material, system or method listed is relevant to your study. If you are not sure if a list item applies to your research, read the appropriate section before selecting a response.

Materials & experimental systems

n/a	Involved in the study
☐	☒ Antibodies
☐	☒ Eukaryotic cell lines
☒	☐ Palaeontology and archaeology
☒	☐ Animals and other organisms
☐	☒ Clinical data
☒	☐ Dual use research of concern

Methods

n/a	Involved in the study
☒	☐ ChIP-seq
☐	☒ Flow cytometry
☒	☐ MRI-based neuroimaging

Antibodies

Antibodies used

These are described including manufacturers. Below are the antibodies or tetramers used, followed by the clone, cataolog number, isotype, lot number, the figure this corresponds to in the paper and dilution used:

CD3 FITC Biolegend UCHT1 300406 Mouse IgG2a, kappa B241015 1b 1/100
CD8a PE/Cy7 Biolegend RPA-T8 301012 κ Mouse IgG1, kappa B291544 1b 1/100
CD4 APC Biolegend OKT4 317416 Mouse IgG2b, kappa B267985 1b 1/100
CD1d-Neg PE Proimmune N A D002 N A SC 0146-06neg 1b 1/100
CD1d α-Gal PE Proimmune N A D001 N A SC 0146-07agc 1b 1/100
IL-4 PE BD FastImmune 340451 Mouse IgG1, k 9315507 1D 1/100
IFNg BUV737 BD Horizon 612845 Mouse IgG1, k 0.199326 1D 1/100
CD279 (PD-1) BV605 Biolegend EH12.2H 7 329924 Mouse IgG1, κ B225643 1E 1/100
CD357 (GITR) PE/Cy7 e Bioscience EBioAITR 25-5875-42 Mouse IgG1, κ 4280320 1E
1/100
CD134/OX40 FITC Biolegend BerACT-35 350006 Mouse IgG1, κ B239452 1E 1/100
CD3 BUV 737 BD Horizon UCHT 1 564307 Mouse IgG1, κ 9136543 1E 1/100
CD4 BUV563 BD Horizon SK3 612912 Mouse IgG1, κ 9136558 1E 1/100
CD8 BUV395 BD Horizon RPA-T 8 563795 Mouse IgG1, κ 8220831 1E 1/100
TIGIT PE eBioscience MBSA43 12-9500-42 Mouse IgG1, κ E13456-108 1E
1/100
CD223/LAG-3 APC eBioscience 3DS2234 17-2239-42 Mouse IgG1, κ 4307377 1E
1/100
CD366/TIM-3 FITC eBioscience F38-2E2 11-3109-41 Mouse IgG1, κ B241987 1E
1/100
CD137/4-1BB PE/Cy7 Biolegend 4B4-1 309818 Mouse IgG1, κ B258325 1E 1/100
CD25 BV510 Biolegend BC96 302640 Mouse IgG1, k B312869 6B 1/100
CD69 PerCP-Cy5.5 Biolegend FN50 310925 Mouse IgG1, k B340058 6B, 6D 1/100
CD25 BV785 Biolegend BC96 302638 Mouse IgG1, k B337500 6D 1/100
Live/Dead APC-Cy7 Thermofisher a L34975 n a 6E 1/100
CD4 BUV563 Biolegend OKT4 750979 Mouse IgG2b, κ 1034509 7i, 8C 1/100
CD8a BUV395 Biolegend RPA-T8 563795 Mouse IgG1, κ 9259666 7i, 8C 1/100
CD25 BV510 Biolegend BC 96 302640 Mouse IgG1, κ B312869 7i, 8C 1/100
CD69 PECy7 Biolegend FN 50 310912 Mouse IgG1, κ B329063 7i, 8C 1/100
PD-1 BV785 Biolegend EH12.2H7 329930 Mouse IgG1, κ B351259 7i, 8C Bs

Validation

As described.

Eukaryotic cell lines

Policy information about cell lines and Sex and Gender in Research

Cell line source(s)

Parent cell lines purchased from ATCC. The source of the cell lines used and the sex of the primary cell lines is now provided.

A375-GFP and A375-GFP-CD1d were derived in-house from A375 melanoma cells (Homo sapiens malignant melanoma derived female cell line, ATCC: CRL-1619). The cells were cultured in DMEM,1X (Corning, NY) supplemented with 10% FBS, 1% Penicillin-Streptomycin (Gemini, Sacramento, CA). For maintenance of CD1d expression in A375-GFP-CD1d cells, puromycin 1ug/ml (Gibco) was added during sub-culture. The A375 cell line is HLA-A2 positive and was confirmed by qPCR to express the cancer testes antigen NY-ESO 1.

Authentication

Cell line identity and species were validated by short tandem repeat (STR) profiling.
A375 cell line profile by STR:
TH01: 8
D5S818: 12
D13S317: 11, 14
D7S820: 9
D16S539: 9

	CSF1PO: 11, 12<br>Amelogenin: X<br>vWA: 16, 17<br>TPOX: 8, 10
Mycoplasma contamination	All cell lines repeatedly tested negative for Mycoplasma.
Commonly misidentified lines<br>(See ICLAC register)	none

Palaeontology and Archaeology

Specimen provenance	*Provide provenance information for specimens and describe permits that were obtained for the work (including the name of the issuing authority, the date of issue, and any identifying information). Permits should encompass collection and, where applicable, export.*
Specimen deposition	*Indicate where the specimens have been deposited to permit free access by other researchers.*
Dating methods	*If new dates are provided, describe how they were obtained (e.g. collection, storage, sample pretreatment and measurement), where they were obtained (i.e. lab name), the calibration program and the protocol for quality assurance OR state that no new dates are provided.*

☐ Tick this box to confirm that the raw and calibrated dates are available in the paper or in Supplementary Information.

Ethics oversight	*Identify the organization(s) that approved or provided guidance on the study protocol, OR state that no ethical approval or guidance was required and explain why not.*

Note that full information on the approval of the study protocol must also be provided in the manuscript.

Animals and other research organisms

Policy information about studies involving animals; ARRIVE guidelines recommended for reporting animal research, and Sex and Gender in Research

Laboratory animals	*For laboratory animals, report species, strain and age OR state that the study did not involve laboratory animals.*
Wild animals	*Provide details on animals observed in or captured in the field; report species and age where possible. Describe how animals were caught and transported and what happened to captive animals after the study (if killed, explain why and describe method; if released, say where and when) OR state that the study did not involve wild animals.*
Reporting on sex	*Indicate if findings apply to only one sex; describe whether sex was considered in study design, methods used for assigning sex. Provide data disaggregated for sex where this information has been collected in the source data as appropriate; provide overall numbers in this Reporting Summary. Please state if this information has not been collected. Report sex-based analyses where performed, justify reasons for lack of sex-based analysis.*
Field-collected samples	*For laboratory work with field-collected samples, describe all relevant parameters such as housing, maintenance, temperature, photoperiod and end-of-experiment protocol OR state that the study did not involve samples collected from the field.*
Ethics oversight	*Identify the organization(s) that approved or provided guidance on the study protocol, OR state that no ethical approval or guidance was required and explain why not.*

Note that full information on the approval of the study protocol must also be provided in the manuscript.

Clinical data

Policy information about clinical studies
All manuscripts should comply with the ICMJE guidelines for publication of clinical research and a completed CONSORT checklist must be included with all submissions.

Clinical trial registration	NCT04582201
Study protocol	The full protocol is provided, alongside a statistical analysis plan.
Data collection	The timelines of recruitment/ treatment are provided in the manuscript.<br><br>a total of 20 individuals with severe COVID-19 and receiving intensive care, were dosed in 3 cohorts between July 2020 and June 2021 (Table 1) and one additional 21-year-old patient was treated under an EUA (EUA patient) for carbapenem-resistant Pseudomonas aeruginosa pneumonia requiring VV-ECMO salvage after clearing SARS-CoV-2 infection (therapeutic schematic at Figure S4).<br><br>We rapidly escalated to the highest dose cohort, as we again we considered there to be a lack of toxicity related to agenT-797. Indeed, the EUA patient was treated at the highest dose level and their carbapenem-resistant Pseudomonas pneumonia rapidly

	cleared post agenT-797 infusion, supporting our view that agenT-797 might be able to assist in the control of secondary infections. Of the 21 agenT-797 treated patients (including the EUA patient), 5 were given agenT-797 during VV-ECMO, traditionally regarded as 'the most aggressive salvage therapy' for critically ill individuals with COVID-19.
Outcomes	The pre-defined primary and secondary outcome measures were reported according to the trial protocol and some of these are descriptive, as per the statistical analysis plan.<br><br>Descriptive statistics are used in displaying the results of the primary endpoint: adverse events and dose-limiting toxicities of agent-797 and secondary endpoints related to improvement and resolution of ARDS, evolution of CRS, avoidance of multiorgan dysfunction syndrome, as well as persistence of agenT-797.<br><br>A treatment-emergent adverse event (TEAE) is defined as an AE that begins or that worsens in severity after the first dose of the study drug. They are coded using MedDRA version 24.1 with severity assessed according to NCI CTCAE version 5.0. Incidence rates of TEAE are summarized by preferred term (PT) including those attributed to the secondary infections. In addition, Incidence rates of TEAE of grade 3 and above, treatment related TEAE (TRAE), TRAE leading to treatment discontinuation, TRAE leading to dose interruption and TRAE leading death are summarized by cohort. For each patient and PT, the worst grade and causality (related to treatment) will be used in the summaries.<br><br>Overall survival is defined as the first dose to date of death due to any cause. A Kaplan Meier plot of survival is provided to show survival rates at pre-defined timepoints of 30 days and 6 months after the start of therapy. Serum cytokines are grouped into CRS markers and proinflammatory and anti-inflammatory markers and their values are plotted at pre-dose, days 1-7 and days 10-28 post dose. Descriptive p-values from an ANOVA model of pre-dose and post-dose serum cytokine level comparisons are provided to show the magnitude of difference in these exploratory analyses. Plots of WBC, iNKT cells and other pharmacodynamic markers are generated to evaluate the treatment effect on these markers.<br><br>Due to the small sample size in this exploratory study, results from the statistical tests are not type I error controlled or adequately powered to make any inferential statement, but rather to provide proof of concept for future studies.

Dual use research of concern

Policy information about dual use research of concern

Hazards

Could the accidental, deliberate or reckless misuse of agents or technologies generated in the work, or the application of information presented in the manuscript, pose a threat to:

No	Yes	
☒	☐	Public health
☒	☐	National security
☒	☐	Crops and/or livestock
☒	☐	Ecosystems
☒	☐	Any other significant area

Experiments of concern

Does the work involve any of these experiments of concern:

No	Yes	
☒	☐	Demonstrate how to render a vaccine ineffective
☒	☐	Confer resistance to therapeutically useful antibiotics or antiviral agents
☒	☐	Enhance the virulence of a pathogen or render a nonpathogen virulent
☒	☐	Increase transmissibility of a pathogen
☒	☐	Alter the host range of a pathogen
☒	☐	Enable evasion of diagnostic/detection modalities
☒	☐	Enable the weaponization of a biological agent or toxin
☒	☐	Any other potentially harmful combination of experiments and agents

ChIP-seq

Data deposition

☐ Confirm that both raw and final processed data have been deposited in a public database such as GEO.

☐ Confirm that you have deposited or provided access to graph files (e.g. BED files) for the called peaks.

Data access links<br>*May remain private before publication.*	*For "Initial submission" or "Revised version" documents, provide reviewer access links. For your "Final submission" document, provide a link to the deposited data.*
Files in database submission	*Provide a list of all files available in the database submission.*
Genome browser session<br>(e.g. UCSC)	*Provide a link to an anonymized genome browser session for "Initial submission" and "Revised version" documents only, to enable peer review. Write "no longer applicable" for "Final submission" documents.*

Methodology

Replicates	*Describe the experimental replicates, specifying number, type and replicate agreement.*
Sequencing depth	*Describe the sequencing depth for each experiment, providing the total number of reads, uniquely mapped reads, length of reads and whether they were paired- or single-end.*
Antibodies	*Describe the antibodies used for the ChIP-seq experiments; as applicable, provide supplier name, catalog number, clone name, and lot number.*
Peak calling parameters	*Specify the command line program and parameters used for read mapping and peak calling, including the ChIP, control and index files used.*
Data quality	*Describe the methods used to ensure data quality in full detail, including how many peaks are at FDR 5% and above 5-fold enrichment.*
Software	*Describe the software used to collect and analyze the ChIP-seq data. For custom code that has been deposited into a community repository, provide accession details.*

Flow Cytometry

Plots

Confirm that:

☒ The axis labels state the marker and fluorochrome used (e.g. CD4-FITC).

☒ The axis scales are clearly visible. Include numbers along axes only for bottom left plot of group (a 'group' is an analysis of identical markers).

☒ All plots are contour plots with outliers or pseudocolor plots.

☒ A numerical value for number of cells or percentage (with statistics) is provided.

Methodology

Sample preparation	iNKT cells and PBMCs were isolated from human donor leukopaks by positive enrichment of iNKT cells using the CliniMACS Plus Instrument (Miltenyi Biotec). PBMC samples were isolated from the negative fraction. iNKT cells were enriched and expanded using proprietary methods, with purity >95%. T cells were isolated from PBMC samples using the EasySep T cell isolation kit (StemCell Technologies, Cat# 17951). DCs and macrophages were generated from CD14+ monocytes isolated from human PBMC samples by polarization culturing with various cytokines as reported in the figures.
Instrument	Flow cytometric samples were collected using either the BD FACSymphony™ A5 (Model # 660937; Becton, Dickinson, and Company) or the BD LSRFortessa™ instrument (Model # 647177; Becton, Dickinson, and Company).
Software	Samples were collected using the BD FACSDiva™ software, with subsequent analyses performed in FlowJo™ Software for Windows, Version 10.7.2/10.8/10.8.1 (Ashland / Becton, Dickinson, and Company).
Cell population abundance	T cells were isolated from PBMC samples using the EasySep T cell isolation kit (StemCell Technologies, Cat# 17951) with post-sort purity of 71.6%, as determined by flow cytometry. T cells were activated and transduced by lentivirus to express TCR of interest. Cells were subsequently expanded using cytokines and transduction efficiency (87%) was determined after expansion by flow cytometry. iNKT cells were isolated from healthy donor leukopaks and enriched. Purity of iNKT cell samples were >95%, as determined by flow cytometry. Enriched DCs had a post-enrichment purity of 77.2%.
Gating strategy	The gating strategy is as follows: lymphocytes were gated using an expanded FSC-A/SSC-A gate, followed by gating on live cells (Live-Dead/SSC-A), and singlets using FSC-A/FSC-H, with subsequent phenotyping of the cells based on markers of interest. Positive and negative gates on cell populations were defined using an appropriate isotype control.<br><br>For experiments involving myeloid populations: cells were gated using an expanded FSC-A/SSC-A gate, followed by gating on singlets using FSC-A/FSC-H, and live cells (Live-Dead/FSC-A), with subsequent phenotyping of the cells based on markers of interest. Positive and negative gates on cell populations were defined using an appropriate isotype control.

☒ Tick this box to confirm that a figure exemplifying the gating strategy is provided in the Supplementary Information.

Magnetic resonance imaging

Experimental design

Design type | *Indicate task or resting state; event-related or block design.*

Design specifications | *Specify the number of blocks, trials or experimental units per session and/or subject, and specify the length of each trial or block (if trials are blocked) and interval between trials.*

Behavioral performance measures | *State number and/or type of variables recorded (e.g. correct button press, response time) and what statistics were used to establish that the subjects were performing the task as expected (e.g. mean, range, and/or standard deviation across subjects).*

Acquisition

Imaging type(s) | *Specify: functional, structural, diffusion, perfusion.*

Field strength | *Specify in Tesla*

Sequence & imaging parameters | *Specify the pulse sequence type (gradient echo, spin echo, etc.), imaging type (EPI, spiral, etc.), field of view, matrix size, slice thickness, orientation and TE/TR/flip angle.*

Area of acquisition | *State whether a whole brain scan was used OR define the area of acquisition, describing how the region was determined.*

Diffusion MRI ☐ Used ☐ Not used

Preprocessing

Preprocessing software | *Provide detail on software version and revision number and on specific parameters (model/functions, brain extraction, segmentation, smoothing kernel size, etc.).*

Normalization | *If data were normalized/standardized, describe the approach(es): specify linear or non-linear and define image types used for transformation OR indicate that data were not normalized and explain rationale for lack of normalization.*

Normalization template | *Describe the template used for normalization/transformation, specifying subject space or group standardized space (e.g. original Talairach, MNI305, ICBM152) OR indicate that the data were not normalized.*

Noise and artifact removal | *Describe your procedure(s) for artifact and structured noise removal, specifying motion parameters, tissue signals and physiological signals (heart rate, respiration).*

Volume censoring | *Define your software and/or method and criteria for volume censoring, and state the extent of such censoring.*

Statistical modeling & inference

Model type and settings | *Specify type (mass univariate, multivariate, RSA, predictive, etc.) and describe essential details of the model at the first and second levels (e.g. fixed, random or mixed effects; drift or auto-correlation).*

Effect(s) tested | *Define precise effect in terms of the task or stimulus conditions instead of psychological concepts and indicate whether ANOVA or factorial designs were used.*

Specify type of analysis: ☐ Whole brain ☐ ROI-based ☐ Both

Statistic type for inference
(See Eklund et al. 2016) | *Specify voxel-wise or cluster-wise and report all relevant parameters for cluster-wise methods.*

Correction | *Describe the type of correction and how it is obtained for multiple comparisons (e.g. FWE, FDR, permutation or Monte Carlo).*

Models & analysis

n/a	Involved in the study
☐	☐ Functional and/or effective connectivity
☐	☐ Graph analysis
☐	☐ Multivariate modeling or predictive analysis

Functional and/or effective connectivity | *Report the measures of dependence used and the model details (e.g. Pearson correlation, partial correlation, mutual information).*

Graph analysis | *Report the dependent variable and connectivity measure, specifying weighted graph or binarized graph, subject- or group-level, and the global and/or node summaries used (e.g. clustering coefficient, efficiency, etc.).*

Multivariate modeling and predictive analysis

Specify independent variables, features extraction and dimension reduction, model, training and evaluation metrics.
